# Genome-Wide Identification, Characterization and Expression Pattern Analysis of the γ-Gliadin Gene Family in the Durum Wheat (*Triticum durum* Desf.) Cultivar Svevo

**DOI:** 10.3390/genes12111743

**Published:** 2021-10-29

**Authors:** Roberta Paris, Giuseppe Petruzzino, Michele Savino, Vanessa De Simone, Donatella B. M. Ficco, Daniela Trono

**Affiliations:** 1 Consiglio per la Ricerca in Agricoltura e l’Analisi dell’Economia Agraria, Centro di Ricerca Cerealicoltura e Colture Industriali, Via di Corticella 133, 40128 Bologna, Italy; roberta.paris@crea.gov.it; 2 Consiglio per la Ricerca in Agricoltura e l’Analisi dell’Economia Agraria, Centro di Ricerca Cerealicoltura e Colture Industriali, S.S. 673, Km 25,200, 71122 Foggia, Italy; giuseppe.petruzzino@crea.gov.it (G.P.); michele.savino@crea.gov.it (M.S.); vanessa.desimone@crea.gov.it (V.D.S.); donatellabm.ficco@crea.gov.it (D.B.M.F.)

**Keywords:** celiac disease, celiac epitopes, durum wheat, gamma-gliadins, gene family, protein expression, qRT-PCR

## Abstract

Very recently, the genome of the modern durum wheat cv. Svevo was fully sequenced, and its assembly is publicly available. So, we exploited the opportunity to carry out an in-depth study for the systematic characterization of the γ-gliadin gene family in the cv. Svevo by combining a bioinformatic approach with transcript and protein analysis. We found that the γ-gliadin family consists of nine genes that include seven functional genes and two pseudogenes. Three genes, *Gli-γ1a*, *Gli-γ3a* and *Gli-γ4a*, and the pseudogene Gli-γ2a* mapped on the A genome, whereas the remaining four genes, *Gli-γ1b*, *Gli-γ2b*, *Gli-γ3b* and *Gli-γ5b*, and the pseudogene Gli-γ4b* mapped on the B genome. The functional γ-gliadins presented all six domains and eight-cysteine residues typical of γ-gliadins. The Gli-γ1b also presented an additional cysteine that could possibly have a role in the formation of the gluten network through binding to HMW glutenins. The γ-gliadins from the A and B genome differed in their celiac disease (CD) epitope content and composition, with the γ-gliadins from the B genome showing the highest frequency of CD epitopes. In all the cases, almost all the CD epitopes clustered in the central region of the γ-gliadin proteins. Transcript analysis during seed development revealed that all the functional γ-gliadin genes were expressed with a similar pattern, although significant differences in the transcript levels were observed among individual genes that were sometimes more than 60-fold. A progressive accumulation of the γ-gliadin fraction was observed in the ripening seeds that reached 34% of the total gliadin fraction at harvest maturity. We believe that the insights generated in the present study could aid further studies on gliadin protein functions and future breeding programs aimed at the selection of new healthier durum wheat genotypes.

## 1. Introduction

Durum wheat (*Triticum durum* Desf.) is one of the most widely consumed staple cereals and is the preferred choice for pasta making. Together with bread wheat, it represents an important source of proteins in the human diet, 80% of which is represented by gluten proteins [1]. Gluten is the water insoluble fraction of wheat flour proteins responsible for the unique biochemical properties of dough. It consists of a mixture of monomeric gliadins (α/β-, γ-, δ- and ω-gliadins), which confer viscosity and extensibility to dough, and high- and low-molecular-weight glutenins (HMW-GS and LMW-GS), which contribute to elasticity and dough strength [1]. Unlike the other dietary proteins, gluten proteins have a high content of proline and glutamine amino acid residues that make them largely inaccessible to gastrointestinal proteases; this results in the accumulation of partially hydrolyzed gluten peptides in the small intestine [2]. These peptides have core consensus sequences of at least nine amino acids, often referred to as celiac epitopes, which activate the immune response after the conversion of specific glutamine residues to glutamate by the intestinal tissue transglutaminase [3]. This imparts negative charges to the gluten peptides that, in genetically predisposed individuals, allow their binding to the positively charged pockets of the HLA-DQ2 and HLA-DQ8 receptors of the antigen presenting cells (APC) [4]. The epitopes are subsequently processed inside the HLA-DQ2 and HLA-DQ8 receptors of the APC and then recognized by the gluten peptide-specific T cells of the intestinal lamina propria, which induce the biological events responsible for the damages to the intestinal mucosa typical of celiac disease (CD) [5]. So far, thirty-one epitopes have been identified in wheat and related species as recognized by the CD4^+^ T cells and, therefore, toxic for celiac patients based on in vitro and in vivo studies [6]. Recently, a role for B cells as the main APC for gluten-specific T cells was postulated [7]. Dørum et al. [8] demonstrated that the antigen receptors of the B cells of CD patients recognize a 7-mer motif (QPQQPFP) that is repeated multiple times in the long peptides generated by gastrointestinal digestive enzymes.

The gliadin fraction of gluten is the main player in the onset of CD because the majority of immunogenic CD epitopes are present in the amino acid sequence of the gliadin proteins. In bread wheat, gliadins are encoded by large multigene families mainly contained in six loci, *Gli-A1*, *Gli-B1* and *Gli-D1* located on the short arms of group 1 chromosomes, and *Gli-A2*, *Gli-B2* and *Gli-D2* on the short arms of group 6 chromosomes. The genes coding for α-gliadins are carried in *Gli-2* loci, whereas those for γ-, δ- and ω-gliadins are in *Gli-1* loci [9]. The most celiac-toxic epitopes were found in most of the α-gliadins encoded by the D genome [10]. Several repeats of some of these epitopes were mapped in a single indigestible 33-mer peptide encompassing the 57–89 N-terminal region of these proteins [10]. The 33-mer was found to be a potent T cell stimulator as it is directly presented for T cell recognition without processing within the APC receptors [11]. Many different CD epitopes were also identified in the γ-gliadins. Like the 33-mer of the α-gliadins, most of the epitopes were found to cluster in the repetitive domain of γ-gliadins [10]. The analysis of 170 γ-gliadin genes isolated from bread wheat and its closely related species revealed that the number of toxic epitopes increased as the length of the repetitive domain increased [12]. Several γ-gliadin-derived peptides were found to elicit the intestinal T cell response in CD patients [8,13,14] and evidence has been reported that the antibody response of CD patients to gluten was directed towards a few immunodominant epitopes, typically displayed in repeats in peptide fragments that mainly derived from γ-gliadins [8].

Durum wheat is a tetraploid species and compared to the hexaploid bread wheat it lacks the D genome; thus, fragments identical or equivalent to the immunodominant 33-mer fragment are absent in gluten of durum wheat. Consistently, evidence exists that durum wheat and the other tetraploid species exhibit lower immunoreactivity compared to bread wheat [15], and that, in these species, the overall response of T cells is directed to γ-epitopes [16]. Very recently, the genome of the modern durum wheat cv. Svevo was fully sequenced [17] and its assembly is publicly available [18]. With the genome sequence availability of durum wheat, we here identified the γ-gliadin gene family in the durum wheat cv. Svevo, investigated the phylogenetic relationship of the γ-gliadin genes and carried out molecular characterization of the γ-gliadin genes and their encoded proteins, with particular attention addressed to the CD epitope content and distribution. Furthermore, we investigated the expression pattern of the γ-gliadins at different stages of seed development at both transcriptional and translational level. Overall, the results obtained in the present study will provide an important reference for future research aimed at developing new durum wheat lines with a reduced content of immunogenic epitopes and, therefore, less harmful for CD patients.

## 2. Materials and Methods

### 2.1. Plant Material and Growth Conditions 

The durum wheat cv. Svevo was chosen for this study. After vernalization, ten seeds per pot were sown in 2.5 L pots containing soil, sand and peat (6:3:1) and the pots were transferred to a growth chamber. Plants were grown under the following conditions until the third leaf stage: 10/8 °C day/night, 12/12 h light/darkness, 60% relative humidity and 500 μmol m^−2^ s^−1^ light intensity. From the third leaf stage onwards, the conditions were gradually modified to reach 28/23 °C day/night, 16/8 h light/darkness, 55% relative humidity and 500 μmol m^−2^ s^−1^ light intensity at harvest maturity. Twenty grams of ammonium nitrate fertilizer were distributed in each pot at sowing and a mix containing 1.2 g mineral superphosphate, 2.0 g ammonium nitrate and 0.1 g potassium sulfate was applied per pot at tillering. Under these growth conditions, plants reached harvest maturity within about four months.

### 2.2. Sequence Analysis of the Genomic Region Harboring the γ-Gliadin Genes in the Durum Wheat cv. Svevo

The assembled genomic regions in the A and B genome of the durum wheat cv. Svevo that carry the γ-gliadin genes were downloaded from the Intranet of Durum Wheat Genome Data [18] and analyzed with the gene prediction program FGENESH [19] to verify the automatically annotated genes and identify any missed genes and pseudogenes. The open reading frames (ORF) identified through FGENESH were verified manually using BLASTN, BLASTP and BLASTX search against the non-redundant NCBI databases. The verified genes were also compared with the gene content from the γ-gliadin regions of the bread wheat cv. Chinese Spring [20].

For the isolation and sequencing of the incomplete TRITD1Av1G002120 gene (hereafter referred to as *Gli-γ1a* gene), the genomic DNA was extracted from 50 mg leaf tissue using the phenol/chloroform, precipitated in 70% ethanol, and dissolved in water. The quality of DNA was evaluated by visualization on agarose gel and its concentration was determined using a QuBit fluorimeter (Thermo Fisher Scientific, Waltham, MA, USA). The ORF of the *Gli-γ1a* gene was amplified using the primer pair 5′-ATGAAGACCTTACTCATCCTGAC-3′ (forward) and 5′-TCATTGGCCACCAATGTCGG-3′ (reverse) and PCR was carried out using the high fidelity Taq DNA polymerase Platinum (Thermo Fisher Scientific, Waltham, MA, USA) under the following conditions: 30 s initial denaturation at 94 °C, then 35 cycles of 15 s at 94 °C, 30 s at 66 °C, and 30 s at 72 °C, followed by 5 min final extension at 72 °C. The product of the amplification was visualized on agarose gel, cloned into pGEM-T Easy Vector (Promega, Madison, WI, USA), and sequenced on both strands using ABI Prism BigDye Terminator Cycle Sequencing kit on an ABI PRISM 3130xl Genetic Analyser (Thermo Fisher Scientific, Waltham, MA, USA). 

### 2.3. Sequence Alignment and Phylogenetic Analysis 

The coding sequences were translated into amino acid sequences and analyzed using Expasy Translate and ProtParam tools [21]. Multiple sequence alignments and phylogenetic tree were constructed by Vector NTI AlignX software (Ver. 9.0, Thermo Fisher Scientific, Waltham, MA, USA). The phylogenetic tree was built using the neighbor joining method. The amino acid sequences were also screened manually for known CD epitopes [3]. Only exact matches were considered.

### 2.4. qRT-PCR 

Total RNA was extracted using Trizol reagent (Thermo Fisher Scientific, Waltham, MA, USA) from durum wheat seeds at the following stages of development: milk stage, M (15 days after anthesis, DAA); early dough stage, ED (25 DAA); hard dough stage, HD (30 DAA); physiological maturity stage, PM (40 DAA); and harvest maturity stage, HM (47 DAA). First-strand cDNA was synthesized from 500 ng total RNA using the SuperScript II RNase H- reverse transcriptase (Thermo Fisher Scientific, Waltham, MA, USA) and random primers. The resulting cDNA was then diluted 1:6 and analyzed by quantitative real-time PCR (qRT-PCR) to evaluate the transcript levels of the full-length γ-gliadin genes, *Gli-γ1a*, *Gli-γ3a*+*Gli-γ4a*, *Gli-γ1b*, *Gli-γ2b*, *Gli-γ3b* and *Gli-γ5b*, and three reference genes, *CDC* (cell division control AAA superfamily of ATPases), *RLI* (RNase L inhibitor-like protein) and *ADP-RF* (ADP-ribosylation factor) [22,23]. The qRT-PCR reactions were performed using the Rotor-Gene 6000 (Qiagen, Hilden, Germany) and the SYBR Green chemistry following the MIQE guideline [24]. Each amplification reaction was prepared in a final volume of 10 μL containing 3 μL of diluted cDNA, 5 μL of Power Up SYBR master mix (Thermo fisher Scientific, Waltham, MA, USA) and specific primers used at the optimized conditions set up in this work and listed in Table S1. The amplification reactions consisted of an initial denaturation at 95 °C for 2 min followed by 40 cycles of denaturation at 95 °C for 15 s and annealing/extension at 60−62 °C for 60 s. All the reactions were carried out in duplicate and three biological replicates were performed for each gene. The transcripts levels of target genes were normalized to the geometric mean of the transcript levels of the three reference genes (*CDC*, *RLI*, *ADP-RF*) and reported as relative amount of transcript levels, expressed as arbitrary units (A.U.).

### 2.5. Gliadin Protein Extraction 

Gliadin proteins were extracted from durum wheat seeds at different stages of development (M, ED, HD, PM, HM) using the procedure reported by Lafiandra and Kasarda [25] with minor modifications. Briefly, seeds were ground under liquid nitrogen by using mortar and pestle. Twenty milligrams of ground powder were extracted twice with 200 μL 1.5 M dimethylformamide by vortexing for 20 min at room temperature. After centrifugation at 10,000× *g* for 10 min at 4 °C the supernatants of the two extractions were collected, mixed and used daily for acid-polyacrylamide gel electrophoresis (A-PAGE) and reversed-phase high-performance liquid chromatography (RP-HPLC) analyses. 

### 2.6. A-PAGE

A-PAGE at pH 3.1 was performed according to the method of Lafiandra and Kasarda [25] with 7% (*w*/*v*) running gel and 5% (*w*/*v*) stacking gel on Hoefer SE 600 Ruby vertical electrophoretic apparatus (GE Healthcare Bio-Sciences Corp, Piscataway, NJ, USA). For each stage of seed development, 10 μL of gliadin extracts were diluted in a 1:1 ratio with a solution containing 60% (*v*/*v*) glycerol and 0.05% (*w*/*v*) pyronin and loaded on gel. Electrophoresis was carried out at 40 mA for 2 h at 15 °C. After electrophoresis, the gel was stained with a mixture of 5 mL 10% (*w*/*v*) Coomassie Blue R-250 dissolved in ethanol and 500 mL 10% (*w*/*v*) trichloroacetic acid, and destained with distilled water for 12 h. The A-PAGE gel was scanned using a Gel Doc 2000 gel imager and analyzed using the Quantity One analysis software scanner (Bio-Rad, Hercules, CA, USA).

### 2.7. RP-HPLC Analysis

Gliadin protein extracts were analyzed by RP-HPLC following a procedure similar to that reported by Mejias et al. [26]. A 1100 Series Quaternary HPLC-System (Agilent Technologies, Palo Alto, CA, USA) was used together with a C8 reversed-phase analytical column (150 × 4.6 mm i.d. Nucleosil 300 A 5 μm particle size) and a diode array UV-Vis detector. The column temperature was set at 60 °C. Two mobile phases were used for linear gradient separation: the polar solvent A consisting of 0.1% trifluoroacetic acid (*v*/*v*) in water and the less polar solvent B containing 0.1% trifluoroacetic acid (*v*/*v*) and acetonitrile. The flow rate was set at 1.0 mL min^−1^. The absorbance was detected at 210 nm and 280 nm. The elution gradient conditions were set as follows: from 0 to 55 min eluent B was increased from 15 to 55%; from 56 to 57 min eluent B was increased from 55 to 90% and then it was maintained at 90% for 3 min. After each run, the column was equilibrated with the starting solvent B concentration for 5 min. The injection volume was 30 μL.

### 2.8. Statistical Analysis

One-way analysis of variance (ANOVA) was carried out on data obtained from qRT-PCR and RP-HPLC analyses. The results were representative of three independent experiments and values were expressed in mean ± S.D. Tukey’s multiple range test was applied to evaluate significant differences (*p* ≤ 0.05) among means.

## 3. Results

### 3.1. Identification and Characterization of the γ-Gliadin Genes in the Genome of the Durum Wheat cv. Svevo

A search was carried out in the genome browser of the durum wheat cv. Svevo to identify genes annotated as γ-gliadins. Six automatically annotated genes were identified of which four, the TRITD1Av1G002070, TRITD1Av1G002120, TRITD1Av1G002200 and TRITD1Av1G002230, were on chromosome 1A, and two, TRITD1Bv1G001870 and TRITD1Bv1G001950, were on chromosome 1B. To verify these genes and identify any missing genes, a manual annotation was performed as described in Materials and Methods. The results of manual annotation and the automatically annotated genes retrieved from the Svevo genome browser are reported in Table 1. To facilitate more detailed analysis, the identified genes were named according to their chromosome location (Table 1). In the regions of the A and B genomes corresponding to the six genes automatically annotated as γ-gliadins, the manual annotation retrieved a total number of sixteen genes (Table 1). Comparison of these gene sequences with those automatically annotated revealed that some gene IDs associated with γ-gliadins in the genome browser covered more than one gene and that some other genes were not detected by automated annotation (Table 1). Nine of the sixteen genes identified by manual annotation encoded typical γ-gliadins; four, *Gli-γ1a*–*Gli-γ4a*, were on chromosome 1A, and five, *Gli-γ1b*–*Gli-γ5b*, on chromosome 1B (Table 1). The other three genes encoded prolamins were initially defined as a novel form of γ-gliadins but subsequently classified as δ-gliadins [27,28]. Two of these genes, Gli-δ1a* and Gli-δ2a*, were located on chromosome 1A, and one, Gli-δ1b*, was on chromosome 1B (Table 1). Finally, four genes encoded avenin-like proteins, two on chromosome 1A (Av-1a* and *Av-2a*) and two on chromosome 1B (Av-1b* and *Av-2b*). One of the nine γ-gliadin gene sequences, TRITD1Av1G002120 (hereafter referred to as *Gli-γ1a*), was found to be incomplete in the genome browser. Therefore, the ORF of this gene was amplified, cloned and sequenced, and the obtained sequence was deposited at the NCBI GenBank database under the accession number MZ399711. 

Sequence analysis revealed that all the identified sequences did not contain introns (Figure S1). The nine γ-gliadin genes included seven full-length genes (*Gli-γ1a*, *Gli-γ3a*, *Gli-γ4a*, *Gli-γ1b*, *Gli-γ2b*, *Gli-γ3b and Gli-γ5b*) with an intact ORF that ranged from 858 to 996 bp, and two pseudogenes (Gli-γ2a* and Gli-γ4b*) (Table 1 and Figure S1). All the δ-gliadin genes (Gli-δ1a*, Gli-δ2a* and Gli-δ1b*) were pseudogenes, whereas the four avenin-like genes included two pseudogenes (Av-1a* and Av-1b*) and two full-length genes (*Av-2a* and *Av-2b*) (Table 1 and Figure S1). All the pseudogenes presented a premature stop codon in their sequence, and the Av-1b* pseudogene was also truncated at bp 465 (Table 1 and Figure S1).

### 3.2. Synteny Comparison of the Homeologous Regions in the A and B Genome of the Durum Wheat cv. Svevo Harboring the γ-Gliadin Genes

**Figure 1:**
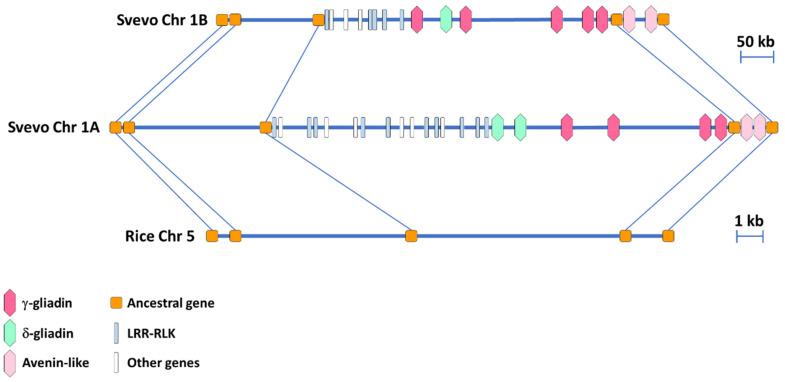
Synteny comparison between the two homoeologous regions of the A and B genomes of the durum wheat cv. Svevo harboring the γ-gliadin genes and the rice orthologous region. The five ancestral genes shared by rice and the durum wheat genomes are represented by orange squares and connected by lines. Hexagons with different colors represent different types of prolamins, leucine-rich repeat receptor-like protein kinases (LRR-RLK) are represented by light blue bars, whereas white bars represent all the other genes.

A synteny analysis was performed between the homeologous regions in the A and B genome of the durum wheat cv. Svevo harboring the γ-gliadin genes. The ancestral genes previously identified in the orthologous region of rice [29] were used to facilitate the analysis. The two homeologous regions in the A and B genome were 1.2 Mb and 0.8 Mb long, respectively, with 29 genes in the A genome and 21 in the B genome (Figure 1 and Table S2). Comparison of the gene content revealed that all the ancestral genes identified in rice were conserved in the homeologous A and B regions. The γ-gliadin genes clustered together with the δ-gliadin genes and these clustering regions were not interrupted by non-prolamin genes (Figure 1 and Table S2). The genomic organization of the regions flanking the γ-/δ-gliadin cluster was quite similar in the A and B genomes, with multiple copies of LRR-RLK genes clustering in the region to the left and the two genes encoding the putative avenin-like proteins to the right (Figure 1 and Table S2).

### 3.3. Phylogenetic Analysis of the γ-Gliadin Genes

**Figure 2:**
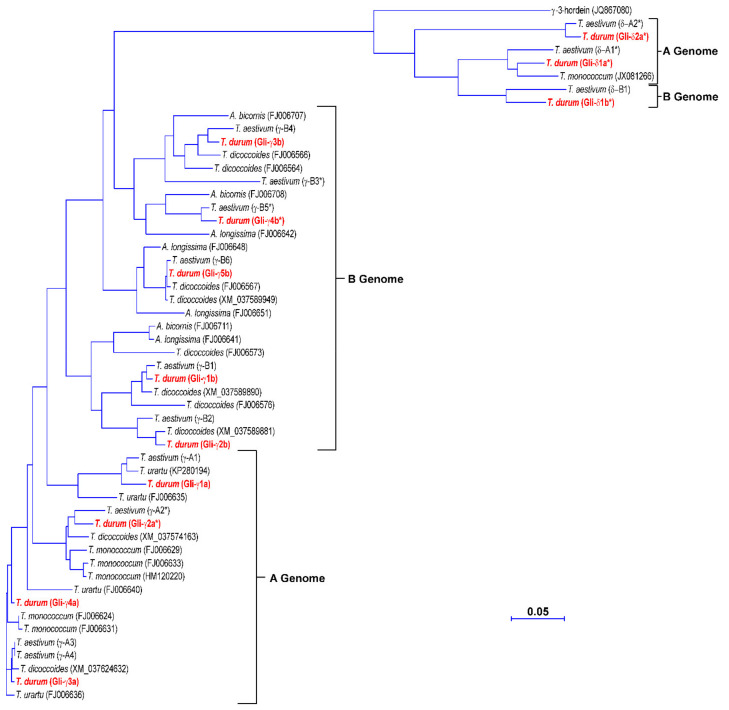
Phylogenetic tree of the amino acid sequences deduced from the γ- and δ-gliadin genes identified in the durum wheat cv. Svevo and other Triticeae species. For *Triticum aestivum*, the amino acid sequences are those reported by Huo et al. [30] and deposited as assembled sequences at the NCBI GenBank database under the accession numbers MG560140 and MG560141 for the A and B genomes, respectively. For pseudogenes, the deduced amino acids downstream of the internal stop codon are also included. Vector NTI Suite software (version 9.0; Thermo Fisher Scientific, Waltham, MA, USA) was used to produce the phylogenetic tree.

The amino acid sequences deduced from the γ-gliadin genes identified in the durum wheat cv. Svevo were compared with those from *Triticum urartu*, *Triticum monococcum*, *Aegilops* species belonging to the *Sitopsis* section and *Triticum dicoccoides*, which cover the diploid and tetraploid species carrying the ancestral genomes of durum wheat, and the A and B genomes of the bread wheat cv. Chinese Spring [30] (Figure 2). Since δ-gliadins were initially classified as a novel type of γ-gliadins, their sequences were also included in the phylogenetic analysis (Figure 2). For the pseudogenes, the deduced amino acids downstream the internal stop codon were also included. The phylogenetic tree distinguished two groups (Figure 2). The main group included all the durum wheat γ-gliadin genes that clustered with γ-gliadins from diploid, tetraploid and hexaploidy species according to their genomic origin. In particular, Gli-γ1a–Gli-γ4a gliadins clustered together with γ-gliadins from the progenitor of the A genome *T. urartu* and its related species *T. monococcum*, and with γ-gliadins located on chromosome 1A of *T. dicoccoides* and the bread wheat cv. Chinese Spring, whereas Gli-γ1b–Gli-γ5b gliadins clustered together with γ-gliadins from *Aegilops* species, which are the progenitors of the B genome, and with γ-gliadins located on chromosome 1B of Chinese Spring and *T. dicoccoides* (Figure 2). 

The δ-gliadins grouped separately together with the γ-3-hordein and other δ-gliadins from *T. monococcum* and the bread wheat cv. Chinese Spring. This result is in line with the orthologous relationship previously observed between this new type of gliadin proteins and γ-3-hordein [27,28]. Even within this small group, a separation based on genome location was observed, with Gli-δ1a* and Gli-δ2a* that clustered with a δ-gliadin from *T. monococcum* and a δ-gliadin assigned to the A genome of the bread wheat cv. Chinese Spring and Gli-δ1b* that clustered with a δ-gliadin assigned to the B genome of Chinese Spring (Figure 2). Consistent with the phylogenetic clustering, the seven full-length genes and the two pseudogenes encoding γ-gliadins shared high identity with each other at both nucleotide (from 73.6 to 99.3%) and amino acid level (67.9% and 98.9%) (Table 2). Conversely, the three δ-gliadin pseudogenes were more closely related to each other (78.5–84.7% identity) than to the γ-gliadin genes and pseudogenes (57.1–62.2% identity); their degree of identity with γ-gliadins was even lower at amino acid level (39.2–47.0%) (Table 2).

### 3.4. Analysis of Deduced Amino Acid Sequences of γ-Gliadins

**Figure 3:**
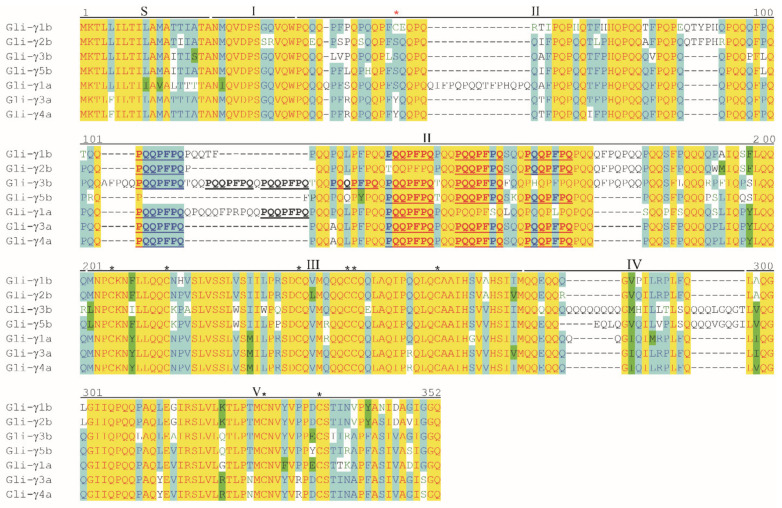
Alignment of the amino acid sequences deduced from the seven full-length γ-gliadin genes identified in the durum wheat cv. Svevo. Roman numerals indicate conserved domains as assigned by Anderson et al. [31]. Black asterisks indicate the eight conserved cysteine residues, whereas red asterisk indicates the extra cysteine residue in the Gli-γ1b. The heptapeptide motif (PQQPFPQ) is highlighted in bold and underlined. Vector NTI Suite software (version 9.0; Thermo Fisher Scientific, Waltham, MA, USA) was used to perform the alignments.

The length of γ-gliadin proteins encoded by the seven full-length genes ranged between 285 and 328 amino acids, whereas the molecular weight ranged between 32,666 and 37,446 Da (Table 3). As already described by Anderson et al. [31], the primary structure of γ-gliadins was composed of a signal peptide (S), a unique N-terminal region (domain I) followed by an alternation of two repetitive regions (domains II and IV) and two non-repetitive regions (domains III and V) (Figure 3). Domain II was rich in proline and glutamine and, in this region, the heptapeptide repeat motif PQQPFPQ typical of γ-gliadins [32] occurred 3–6 times. Domain IV contained tandem glutamine residues encoded by a series of glutamine CAA codons sometimes interrupted by codons such as CAG, GAA or CTA that derived from a single base mutation of the CAA codon (Figure S2a). The longest of this poly-Q sequence was in the Gli-γ3b (Figure 3).

All the γ-gliadins contained eight cysteine residues (Table 3) that followed a conserved pattern [31]: six cysteines were in domain III, with the fourth and the sixth cysteine in consecutive positions, and two cysteines were in domain V (Figure 3). The Gli-γ1b contained an extra cysteine residue in domain II (Table 3 and Figure 3). Comparison between the gene sequences showed that this additional cysteine in the Gli-γ1b protein was due to a point mutation at bp 134 that changed TCC or TAC codon to TGC codon (Figure S2a). 

As expected, the analysis of the amino acid composition revealed that glutamine and proline were the most abundant amino acids, with glutamine content that ranged between 29.1 (in the Gli-γ1b) and 34.1% (in the Gli-γ3b) and proline content that ranged between 15.1 (in the Gli-γ5b) and 17.1% (in the Gli-γ1a) (Table S3). Among the essential amino acids, the highest percentage was observed for leucine (from 6.7 to 8.2%) and the lowest for tryptophan (from 0.3 to 0.9%) (Table S3). Overall, the percentage of total essential amino acids was on average 32%, with the highest percentage observed for Gli-γ5b (33.8%) (Table S3). 

The amino acid sequences deduced from the Gli-γ2a* and Gli-γ4b* pseudogenes also presented the typical primary structure of γ-gliadins with the six conserved domains, the eight cysteines and the heptapeptide repetitive motif (Figure S3a). Regarding the δ-gliadin pseudogenes, their deduced amino acid sequences presented the six domains and the eight conserved cysteines typical of γ-gliadins but lacked the heptapeptide repetitive motif (Figure S3b). The premature stop codon in both γ- and δ-gliadin pseudogenes resulted from the substitution from C to T that changed the CAG or CAA codons for glutamine residue to TAG or TAA stop codons (Figure S2a,b).

Comparison between the amino acid sequences deduced from the γ-gliadin genes of cv. Svevo and those deduced from γ-gliadin genes of other durum wheat cvs. clearly showed that these proteins shared a high level of identity with all the domains and the repeats of the heptapeptide motif strongly conserved, thus revealing a very low variability of this class of proteins within the durum wheat species (Figure S4).

### 3.5. Differences in CD Epitopes among γ-Gliadins 

**Figure 4:**
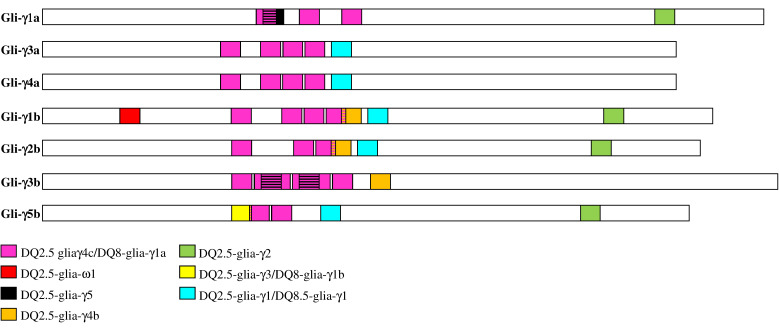
Distribution of CD epitopes across the seven functional γ-gliadins identified in the durum wheat cv. Svevo. The colored boxes indicate the different CD epitopes already identified in γ-gliadins from wheat [6]. Overlapping epitopes are represented by two-color horizontal stripes.

Due to the increasing evidence on the role of γ-gliadin epitopes in the development of CD, a bioinformatic analysis of the amino acid sequences was carried out to identify the type and distribution of CD epitopes in the γ-gliadins encoded by the seven full-length genes of the cv. Svevo. The results obtained are reported in Table 4 and schematically presented in Figure 4. 

The seven γ-gliadins were found to have diverse and numerous CD epitopes, which differed for their distribution across the different proteins. The DQ2.5-glia-γ4c/DQ8-glia-γ1a epitopes were detected in all the γ-gliadins, the DQ2.5-glia-γ1/DQ8.5-glia-γ1 epitopes were found in all the γ-gliadins except the Gli-γ1a and the Gli-γ3b, which instead presented the DQ2.5-glia-γ5 epitope that was absent in the other γ-gliadins (Table 4 and Figure 4). Interestingly, the DQ2.5-glia-γ3/DQ8-glia-γ1b, DQ2.5-glia-γ4b and DQ2.5-glia-ω1 epitopes were detected exclusively in the γ-gliadins of the B genome. In particular, the DQ2.5-glia-ω1 epitope was found only in the Gli-γ1b, whereas the DQ2.5-glia-γ3/DQ8-glia-γ1b epitopes were found exclusively in the Gli-γ5b (Table 4 and Figure 4). All the epitopes were present as a single copy except for the DQ2.5-glia-γ5 epitope that was present in double copy in the Gli-γ3b, and the DQ2.5-glia-γ4c/DQ8-glia-γ1a epitopes that were repeated in multiple copies (ranging from 2 to 6) in all the γ-gliadins (Table 4 and Figure 4). Except for the DQ2.5-glia-ω1 and the DQ2.5-glia-γ2 epitopes that mapped at the ends of proteins, all the other CD epitopes clustered in the middle region of the protein sequences (Figure 4) that corresponded to domain II (Figure 2).

### 3.6. Transcriptional Profile of γ-Gliadins during Grain Development

**Figure 5:**
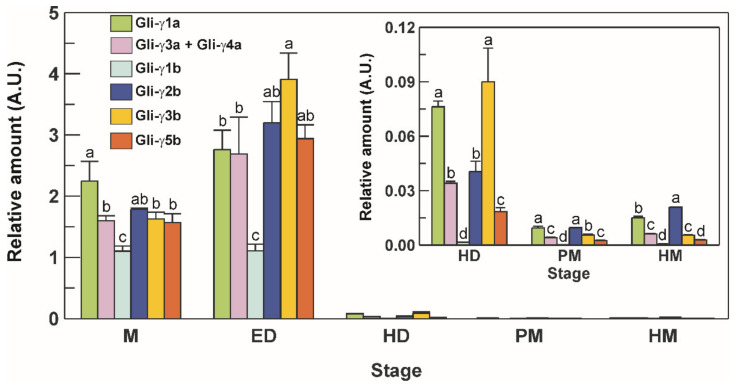
Transcriptional levels of the seven full-length γ-gliadin genes in seeds from the durum wheat cv. Svevo at different stages of development. M, milk stage; ED, early dough stage; HD, hard dough stage; PM, physiological maturity stage; HM, harvest maturity stage. A.U., arbitrary units. Vertical bars represent the S.D. of the mean value of three independent experiments. For each stage, statistical differences are shown by different letters above columns (Tukey’s test, *p* ≤ 0.05).

An experiment was carried out to assess the transcript profile of the full-length γ-gliadin genes in durum wheat grains at different stages of development. To do this, a set of highly specific primer pairs was developed to differentiate the level of transcription of each single member of the γ-gliadin gene family, except for *Gli-γ3a* and *Gli-γ4a* genes that, due to their high identity level (99.3%, see Table 2), were evaluated as a whole by using the same primer pair. The transcriptional profile of these genes showed the same pattern in general, although significant differences among genes emerged from the analysis (Figure 5). 

At milk (M) stage, the highest expression levels were observed for *Gli-γ1a*, the lowest for *Gli-γ1b*, whereas the other genes were expressed at comparable levels (Figure 5). Except for *Gli-γ1b*, which maintained stable levels, an increase was observed for all the other genes at early dough (ED) stage, with *Gli-γ3b* showing the highest levels (Figure 5). A sharp decrease in the transcriptional levels of all the genes was observed from ED up to hard dough (HD) stage, and a further decline until harvest maturity (HM) stage, with *Gli-γ1a* and *Gli-γ2b* that maintained the highest levels until maturity, and *Gli-γ1b* that dropped to very low, but still detectable, levels (Figure 5). 

### 3.7. Accumulation Pattern of Gliadin Proteins during Grain Development

**Figure 6:**
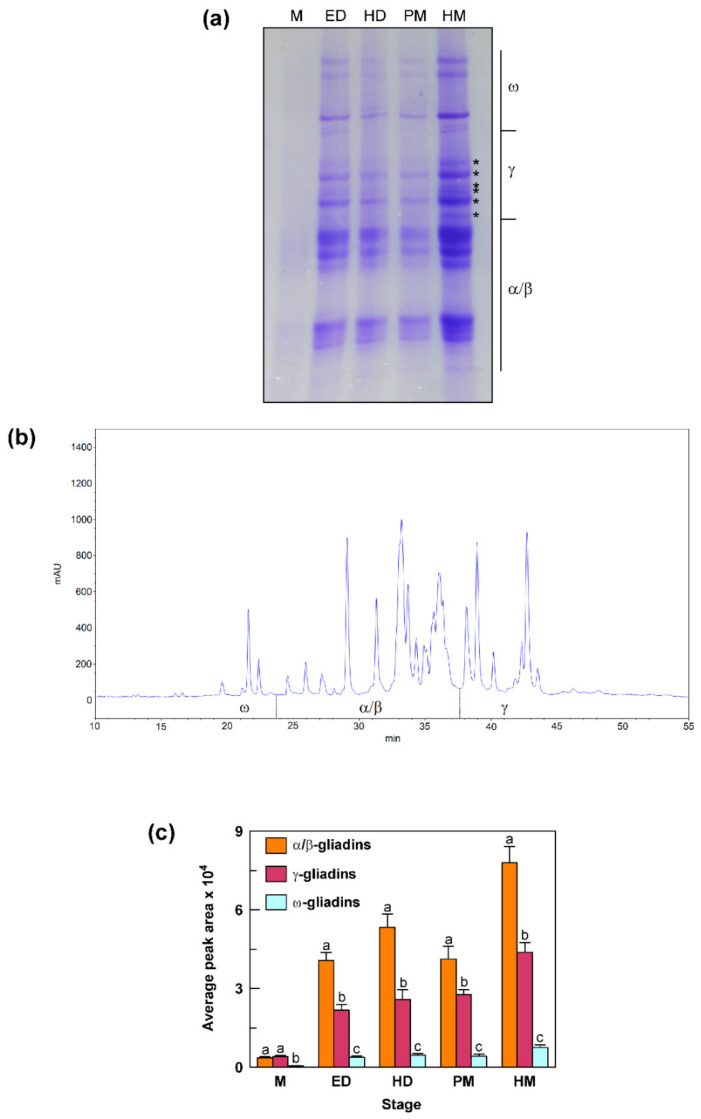
Accumulation pattern of gliadin proteins in seeds from the durum wheat cv. Svevo at different stages of development obtained by A-PAGE (**a**) and RP-HPLC (**b**,**c**). M, milk stage; ED, early dough stage; HD, hard dough stage; PM, physiological maturity stage; HM, harvest maturity stage. In (**a**) the bands corresponding to γ-gliadins are indicated with an asterisk. In (**b**) a typical RP-HPLC chromatogram obtained at harvest maturity is reported. In (**c**) the gliadin levels calculated using the area covered by specific gliadin groups on the RP-HPLC chromatogram are reported. Vertical bars represent the S.D. of the mean value of three independent experiments. For each stage, statistical differences are shown by different letters above columns (Tukey’s test, *p* ≤ 0.05).

To assess the contribution of γ-gliadin proteins to the total gliadin content, seeds at different stages of development (the same used for the transcriptional analysis) were examined by A-PAGE and RP-HPLC for the accumulation of gliadin proteins in their endosperm (Figure 6). Overall, the A-PAGE revealed that the accumulation of the different gliadin fractions (α/β, γ and ω) increased as the seed ripening progressed (Figure 6a). A strong increase was observed from M stage to ED and HD stages; after a decrease at PM stage, gliadin content reached its maximum at HM stage. For the γ-gliadin fraction, the A-PAGE separation allowed distinguishing of at least six bands indicated with an asterisk (Figure 6a). The RP-HPLC analysis allowed a good separation of the peaks in the γ- and ω-gliadin zones, whereas the peaks corresponding to the α/β gliadins were difficult to separate (Figure 6b). This difficulty has already been reported for bread wheat and can be explained by the grand average of the hydropathicity index (GRAVY) that was found to be much more similar among α-gliadins than γ- and ω-gliadins [33]. Furthermore, in this case, at least six peaks were distinguishable in the chromatographic zone corresponding to the γ-gliadin fraction (Figure 6b). The gliadin levels calculated using the area covered by specific gliadin fractions on the RP-HPLC chromatograms confirmed the trend observed in the A-PAGE, with a progressive increase in the protein levels from M to the HM stage, except for a decrease (mainly for α/β gliadins) at PM stage (Figure 6c). At M stage, γ-gliadins accounted for 51% of the total gliadin content, whereas 43% and 6% of total gliadins was represented by α/β-gliadins and ω-gliadins, respectively (Figure 6c). From the M stage onwards, γ-gliadins decreased to 31–38%, α/β-gliadins increased to 56–63%, whereas ω-gliadins remained unchanged (6%) (Figure 6c).

## 4. Discussion

Most of the wheat gliadin classes have been deeply investigated. Studies on γ-gliadins have focused on a comparative analysis of γ-gliadin genes from diploid, tetraploid and hexaploidy wheats and related wild grasses [12,34] and have yielded important information on the origin and evolution of this multigene family. However, to date, in-depth studies for the systematic characterization of the γ-gliadin gene family in a single species have been reported only for the bread wheat [10,35]. The recent availability of the durum wheat genome has opened new possibilities for gene discovery and breeding efforts in this important crop, and we have exploited this opportunity to investigate the γ-gliadin family in the durum wheat cv. Svevo by combining a bioinformatic approach with transcript and protein analysis. The results obtained have shed light on questions that, up to date, have not been exhaustively addressed in durum wheat, that is (i) the exact number and chromosomal position of the γ-gliadin genes, (ii) the distribution of CD epitopes within the encoded protein sequences and (iii) their expression levels during grain filling. These findings and their usefulness in future studies are discussed below.

### 4.1. The γ-Gliadin Gene Family in the Durum Wheat cv. Svevo

Throughout a genome-wide search, the complete γ-gliadin gene family was identified in the tetraploid wheat cv. Svevo. It included seven full-length actively transcribed genes, three on the A genome and four on the B genome, and two pseudogenes, one for each genome. The phylogenetic analysis showed that the γ-gliadin genes from the A and B genomes clustered together separately with the γ-gliadins from the diploid *Aegilops* and *Triticum* species that are close relatives of the diploid ancestor of durum wheat. This indicates that there are two distinct groups of γ-gliadins, one for each of the two genomes of the tetraploid wheat and this is in line with the notion that the duplication and expansion of γ-gliadin gene family in the A and B genomes occurred independently [36]. Comparison between γ-gliadins from the cv. Svevo and the diploid species carrying the A and B genomes suggested that the evolution and divergence of these genes occurred both before and after polyploidization. Indeed, most of the γ-gliadins identified in the cv. Svevo have highly similar (between 90 and 99%) orthologous counterparts in the diploid species. *Gli-γ3a* and *Gli-γ4a* genes instead have only one copy in the diploid species, thus suggesting a relatively recent duplication event. Furthermore, pseudogenization of the Gli-γ2a* and Gli-γ4b* genes occurred after polyploidization since their orthologous counterparts in the diploid species are full-length genes. In particular, the pseudogenization of the Gli-γ4b* gene seems a rather recent event as this gene is also present in its functional form in some durum wheat cvs. (see Figure S4).

The number of active γ-gliadin genes identified in the present study in the Svevo genome is in line with that previously reported for bread wheat. Previous studies on different hexaploidy wheat cvs. have indeed reported the existence of eleven full-length γ-gliadin genes [10,30,35]. Of these, three were assigned to the A genome and four to both the B and D genome [10,30]. The seven intact ORFs identified in the present study are also consistent with the seven γ-gliadin spots identified on 2D-PAGE of soluble proteins from durum wheat seed by De Santis et al. [37]. In the present study, we observed at least six γ-gliadin bands in the A-PAGE of the soluble protein grain fraction of the cv. Svevo and as many peaks ascribable to γ-gliadins in the RP-HPLC chromatogram. This result could be explained with the high identity shared by *Gli-γ3a* and *Gli-γ4a* genes encoding almost identical polypeptides that could hardly be separated; in addition, the existence of an extra cysteine residue in the Gli-γ1b could allow this protein to enter intermolecular aggregates (see below), which would be discarded with the insoluble protein fraction. Due to its very low expression in the final stages of seed ripening, it is also feasible that the amount of Gli-γ1b protein in mature seeds is below the detection level. It is, therefore, reasonable to assume that the number of full-length genes identified in the cv. Svevo is consistent with the number of γ-gliadins estimated through the analysis of the soluble protein fraction. 

The γ-gliadin gene family of the cv. Svevo also included two pseudogenes. The gliadin proteins contain a high percentage of glutamine residues encoded by CAA and CAG codons that can be mutated into stop codons through C→T transitions of the first codon base. This phenomenon has already been reported and is particularly relevant for α-gliadins that have a high rate of pseudogenization, with nearly half of the genes identified as pseudogenes [38]. In cv. Svevo the insertion of a premature stop codon also determined the pseudogenization of all the genes encoding δ-gliadins. Due to the structural organization, which is similar to that of γ-gliadins, some authors initially classified these proteins as novel γ-gliadins [28] and this also explains their automatic annotation as γ-gliadins in the Svevo genome. However, these proteins share low identity with the typical γ-gliadins (39–47%), whereas they share high identity (54–95%) and the same chromosomal position with the δ-gliadins already identified in the hexaploidy cv. Chinese Spring [30]. All the δ-gliadins genes identified in the durum wheat cv. Svevo are pseudogenes. Although pseudogenization of all the δ-gliadin genes has also been found in the durum wheat cvs. Strongfield and Cappelli (Table S4), the existence of durum wheat genotypes with functional δ-gliadins cannot be excluded. Indeed, the analysis of δ-gliadins genes from different bread wheat cvs. revealed that, except for the orthologous of the Gli-δ2a* gene, which in all the bread wheat cvs. analyzed is present as pseudogene, the orthologous of the Gli-δ1*a and Gli-δ1b* genes exist both as pseudo- and full-length genes (Table S4).

### 4.2. Characteristics of the Svevo γ-Gliadins

Gliadins, together with other gluten proteins, play important roles in the technological and nutritional properties of wheat flour. Except for Gli-γ1b, all the γ-gliadins in the cv. Svevo presented an even number of cysteine residues involved in the formation of intrachain disulfide bonds. These bonds are responsible for the folded structure of γ-gliadins and determine the type of noncovalent protein–protein interactions responsible for the extensibility of the gluten network [39]. Gli-γ1b had an additional cysteine residue apart from the conserved ones, giving this γ-gliadin nine total cysteine residues. The observation that in durum wheat the only γ-gliadin with an additional cysteine residue was encoded by a gene on the B genome is in line with previous findings that all the *Aegilops* species had genes that encode γ-gliadins with nine cysteines [36]. As already hypothesized [36,40], it is feasible that this nine-cysteine γ-gliadin has a function in the formation of the gluten network through its binding to HMW glutenins or other gluten proteins. Consistently, direct experimental proofs for the existence of an interchain bond between γ-gliadins and LMW subunits have been obtained by sequence analysis of peptides from enzymatic digests of gluten proteins [41,42]. Moreover, Ferrante et al. [43] isolated a gene encoding a γ-gliadin with nine cysteines from the durum wheat cultivar Lira biotype 45 and, through an integrated approach involving heterologous expression, 2-DE, RP-HPLC and MS, demonstrated that it was expressed in planta and that the corresponding protein was incorporated in the glutenin fraction.

As regards the CD epitope content, our analysis showed that the γ-gliadins from the two genomes encode a large number of different immunogenic peptides. As already reported, these exceed the number of identified immunogenic peptides in the α-gliadins [10] and there is increasing evidence about their relevance in stimulating the intestinal T cell response in CD patients. Camarca et al. [14] reported that the 33-mer of the α-gliadins was recognized by gliadin reactive T cell lines only in 50% of the cohort of CD patients, whereas γ-gliadin peptides were recognized by 78% of CD patients. Moreover, the authors observed that the T cell reactivity towards α-gliadins was directed against a few immunodominant peptides, whereas the reactivity towards γ-gliadins was more heterogeneous and directed against a large panel of immunogenic peptides [14]. These peptides spanned the region from the amino acid residue 78 and 236 that in the γ-gliadins from the durum wheat cv. Svevo corresponded to the region in which almost all the epitopes are clustered. In line with this observation, Dørum et al. [8] found that more than 80% of all peptides pulled down with six different monoclonal antibodies generated from CD lesions contained multiple repeats of γ-gliadin T cell epitopes, with the DQ2.5-glia-γ4c/DQ8-glia-γ1a (QQPQQPFPQ) and the DQ2.5-glia-γ5 (QQPFPQQPQ) epitopes being the most frequent (up to 84% and 60% of the peptides, respectively). Interestingly, peptides generated from the central region of the γ-gliadin proteins also contain multiple repeats of the 7-mer motif (QPQQPFP) recognized by the antigen receptors of the B cells of CD patients [8]. The coexistence in the same γ-gliadin peptides of T cell and B cell epitopes in multiple repeats may allow the gluten-specific B cells to bind and display a multiplicity of T cell epitopes simultaneously, thus inducing a strong antibody response. The γ-gliadins from the A and B genome clustered separately and this difference was also reflected on the occurrence of CD epitopes. Indeed, γ-gliadins encoded by genes in the B genome of the durum wheat cv. Svevo showed the highest presence of CD epitopes compared to the A genome. If the highest toxicity of γ-gliadins in the B genome will be confirmed by in vivo experiments, lowering the level of these proteins could be a valuable goal to be pursued in future breeding programs aimed at obtaining new durum wheat lines with reduced CD toxicity. In this regard it should be emphasized that the very low variability of γ-gliadins within the durum wheat species makes the traditional crossing between durum wheat lines an approach that can hardly be applied and points to genetic engineering as the most suitable way for obtaining new durum wheat lines which are healthier for people with CD. Consistently, promising wheat lines with altered γ-gliadin profiles have been obtained by RNAi [44] and CRISPR/Cas9 [45] approaches. 

### 4.3. Expression of Durum Wheat γ-Gliadins during Grain Development 

Based on our findings it can be assumed that during the grain development of the cv. Svevo the full-length *γ*-gliadin genes are all transcribed and have their proteins accumulated in the mature grains. Seed ripening was characterized by strong changes in the expression of the γ-gliadin genes, which shared a similar transcriptional pattern throughout the grain filling period. Significant transcript levels were detected at milk stage (15 DAA) that peaked at early dough stage (25 DAA) and then fell to very low levels at maturity (40–47 DAA). The transcriptional profiling in developing wheat seeds has been deeply investigated by using cDNA microarrays and evidence has been reported that the maximum rate of storage protein transcript accumulation occurred between 7 and 14 DAA and peaked at 21 DAA [46]. As specifically regards γ-gliadins, a pattern like that observed in the present study has also been observed in two bread wheat lines, although in this latter case the primer pairs used amplified groups of γ-gliadin genes and did not provide information on the single genes within each group [47]. As far as we know, the present study is the first report that describes the transcriptional pattern for each member of the entire γ-gliadin gene family during wheat grain development by qRT-PCR analysis. The availability of this tool for γ-gliadin transcript analysis will be useful whenever a gene editing approach would be used against one specific or more than one sequence of the γ-type, to verify the effect of editing on target gene transcription and on the transcription of the other members of the family. In fact, using this assay based on qRT-PCR it is very easy and quick to realize and allow for easy verification of the success of gene editing. Moreover, as these highly specific primer pairs can distinguish single genes, they can be used as SCAR (sequence characterized amplified regions) functional markers simply by performing PCR reactions, for a precision durum breeding program to select or counter-select for particular γ-gliadin genes according to their function in either plants, for human health, or both. Although γ-gliadin genes showed the same expression pattern, significant differences in the transcript levels were observed among individual genes that were sometimes more than 60-fold. Differential expression levels within the γ-gliadin gene family have been also observed in the bread wheat cv. Chinese Spring [35]. Expression divergence of prolamin genes is a common phenomenon in wheat [30,35] and other cereal species [48]. Genomic and functional genomics information obtained in the last 10 years indicate that the expression of prolamin genes is regulated by complex interactions between several *cis*- and *trans*-acting factors and that differences in these interactions may be responsible for variations in the expression of individual glutenin and gliadin genes [49].

All the gliadin fractions including γ-gliadins gradually accumulated from 15 DAA until maturity. This is expected since a progressive accumulation of the different storage protein fractions occurs from flowering until maturity [50]. In particular, a strong increase was observed from 15 DAA, when seed accumulates more water than dry matter, to 25–30 DAA, when seed water loss occurs accompanied by increasing protein and starch deposition [50]. Our findings indicate that γ-gliadins are abundant in durum wheat, comprising 34% of the total gliadin fraction in harvest seeds. So, the abundance, together with the multiple repeats of toxic epitopes typical of the γ-gliadin proteins, make their encoding genes a suitable target for advanced molecular breeding approaches aimed at downregulating or mutating these CD-toxic proteins.

## 5. Conclusions

Overall, the present study represents a comprehensive analysis of the γ-gliadin gene family in the durum wheat cv. Svevo. Our effort to sequence and manually annotate these and the surrounding genes provided an accurate identification of the γ-gliadin gene family and its position in the durum wheat genome. The results gained from this study will serve as a solid foundation that will facilitate proteomic studies on the role of durum wheat γ-gliadins in semolina functionality and human health, as well as future breeding programs aimed at the selection of new durum wheat genotypes with reduced immunogenic potential for human consumption.

## Figures and Tables

**Table 1:** Comparison between automatic and manual annotation of the regions in the A and B genome of the durum wheat cv. Svevo that carry the γ-gliadin genes.

Automatic Annotation						Manual Annotation					
Chr	Gene ID	Start	Stop	Size (bp)	Annotation	Start	Stop	Size (bp)	Annotation	Gene/ Pseudogene	Note
1A	TRITD1Av1G002070	4,613,285	4,614,202	918	γ-gliadin	4,613,285	4,614,202	918	δ-gliadin (Gli-δ1a*)	pseudogene	TAG at bp 247
1A	N.A. ^a^	—	—	—	—	4,631,881	4,632,772	892	δ-gliadin (Gli-δ2a*)	pseudogene	TAA at bp 163
1A	TRITD1Av1G002120	4,687,840	4,688,874	1035	γ-gliadin	4,687,840	4,688,874	966 ^b^	γ-gliadin (*Gli-γ1a*)	full-length gene	
1A	N.A.	—	—	—	—	4,744,130	4,7449,69	840	γ-gliadin (Gli-γ2a*)	pseudogene	TAG at bp 115
1A	TRITD1Av1G002200	4,871,918	4,880,492	8575	γ-gliadin	4,871,918	4,872,775	858	γ-gliadin (*Gli-γ3a*)	full-length gene	
1A						4,879,635	4,880,492	858	γ-gliadin (*Gli-γ4a*)	full-length gene	
1A	N.A.	—	—	—	—	4,887,801	4,888,553	753	avenin-like (Av-1a*)	pseudogene	TAG at bp 169
1A	TRITD1Av1G002230	4,891,405	4,892,007	603	γ-gliadin	4,891,405	4,892,007	603	avenin-like (*Av-2a*)	full-length gene	
1B	TRITD1Bv1G001870	4,313,476	4,519,819	206,344	γ-gliadin	4,313,476	4,314,384	909	γ-gliadin (*Gli-γ1b*)	full-length gene	
						4,341,737	4,342,690	954	δ-gliadin (Gli-δ1b*)	pseudogene	TAG at bp 277
						4,355,195	4,356,088	894	γ-gliadin (*Gli-γ2b*)	full-length gene	
						4,481,997	4,482,992	996	γ-gliadin (*Gli-γ3b*)	full-length gene	
						4,514,026	4,515,024	999	γ-gliadin (Gli-γ4b*)	pseudogene	TAA at bp 196
						4,518,944	4,519,819	876	γ-gliadin (*Gli-γ5b*)	full-length gene	
1B	N.A.	—	—	—	—	4,528,580	4,529,044	465	avenin-like (Av-1b*)	pseudogene	TAG at bp 79 Truncated at bp 465
1B	TRITD1Bv1G001950	4,543,310	4,543,921	612	γ-gliadin	4,543,310	4,543,921	612	avenin-like (*Av-2b*)	full-length gene	

^a^ N.A. not annotated. ^b^ This size was derived from sequencing of the TRITD1Av1G002120 gene and was found to be lower than that deduced from the gene start and stop positions in the genome browser.

**Table 2:** Percentage of identity among the nucleotide sequences of the γ- and δ-gliadin genes identified in the durum wheat cv. Svevo (section above diagonal) and among their deduced amino acid sequences (section below diagonal).

	Gli-γ1a	Gli-γ2a*	Gli-γ3a	Gli-γ4a	Gli-γ1b	Gli-γ2b	Gli-γ3b	Gli-γ4b*	Gli-γ5b	Gli-δ1a*	Gli-δ2a*	Gli-δ1b*
**Gli-γ1a**	**100**	79.6	82.4	82.2	83.7	83.2	80.1	79.6	77.5	57.8	59.6	59.6
Gli-γ2a*	76.4	**100**	89.5	89.6	83.7	85.5	74.6	73.6	80.9	59.0	59.8	60.0
**Gli-γ3a**	78.2	88.5	**100**	99.3	86.5	87.9	76.3	76.4	85.4	60.4	59.6	61.4
**Gli-γ4a**	77.9	88.9	98.9	**100**	86.6	87.9	76.5	76.5	85.5	60.4	59.7	61.1
**Gli-γ1b**	76.4	78.9	82.5	82.5	**100**	93.2	80.2	76.7	80.0	57.1	57.4	60.0
**Gli-γ2b**	76.9	80.5	83.2	82.5	88.4	**100**	78.6	75.9	81.4	57.2	57.7	51.6
**Gli-γ3b**	72.2	69.0	71.0	71.3	73.2	71.3	**100**	86.5	80.1	59.4	59.2	59.7
Gli-γ4b*	73.3	67.9	70.9	70.9	68.7	67.5	81.9	**100**	80.6	58.6	57.5	59.0
**Gli-γ5b**	73.0	78.4	81.8	82.1	75.5	76.3	75.5	76.6	**100**	62.2	60.9	61.8
Gli-δ1a*	42.9	43.3	43.9	45.4	42.4	42.1	43.9	43.5	47.0	**100**	82.9	84.7
Gli-δ2a*	40.1	40.7	40.7	41.0	41.0	41.1	41.6	39.2	43.7	74.4	**100**	78.5
Gli-δ1b*	45.7	45.6	46.6	46.3	42.2	44.0	45.2	44.1	46.3	80.7	69.8	**100**

**Table 3:** Characteristics of the γ-gliadin proteins encoded by the seven full-length genes identified in the durum wheat cv. Svevo.

Protein	Length (aa)	Number of Cysteines	Predicted MW (Da)	Predicted Pi
Gli-γ1a	321	8	36607	8.16
Gli-γ3a	285	8	32666	8.70
Gli-γ4a	285	8	32678	8.70
Gli-γ1b	302	9	34302	6.62
Gli-γ2b	296	8	33652	8.16
Gli-γ3b	328	8	37446	8.17
Gli-γ5b	291	8	32994	8.48

**Table 4:** CD epitopes in the seven γ-gliadin proteins encoded by the full-length genes identified in the durum wheat cv. Svevo.

HLA	Epitope	Sequence *	Gli-γ1a	Gli-γ3a	Gli-γ4a	Gli-γ1b	Gli-γ2b	Gli-γ3b	Gli-γ5b
DQ2.5	DQ2.5-glia-γ1	PQQSFPQQQ		1	1	1	1		1
	DQ2.5-glia-γ2	IQPQQPAQL	1			1	1		1
	DQ2.5-glia-γ3	QQPQQPYPQ							1
	DQ2.5-glia-γ4b	PQPQQQFPQ				1	1	1	
	DQ2.5 glia-γ4c	QQPQQPFPQ	3	4	4	4	3	6	2
	DQ2.5-glia-γ5	QQPFPQQPQ	1					2	
	DQ2.5-glia-ω1	PFPQPQQPF				1			
DQ8	DQ8-glia-γ1a	QQPQQPFPQ	3	4	4	4	3	6	2
	DQ8-glia-γ1b	QQPQQPYPQ							1
DQ8.5	DQ8.5-glia-γ1	PQQSFPQQQ		1	1	1	1		1

* According to Shewry and Tatam [3], deaminated glutamine residues are highlighted in bold, whereas other glutamine residues, potential targets of tissue transglutaminase, are underlined.

## Data Availability

The sequence of the *Gli-γ1a* gene is available at NCBI GenBank database under the accession number MZ399711.

## Supplementary Materials

The following are available online at https://www.mdpi.com/article/10.3390/genes12111743/s1, Figure S1: Sequences of the γ-gliadin, δ-gliadin and avenin-like genes reported in Table 1 and their deduced translation products, Figure S2: Alignment of the γ-gliadin (a) and δ-gliadin (b) gene sequences from the durum wheat cv. Svevo, Figure S3: Alignment of the amino acid sequences deduced from the pseudogenes encoding γ-gliadins (a) and δ-gliadins (b), Figure S4: Alignment of the amino acid sequences deduced from the γ-gliadin genes of the cv. Svevo and other durum wheat cvs., Table S1: Primer pairs and optimized conditions used in the qRT-PCR, and size of the amplified products, Table S2: Manual annotation of the regions from the homeologous A and B genome of the durum wheat cv. Svevo harboring the γ-gliadin genes, Table S3: Percentage of glutamine, proline and essential amino acids in the γ-gliadin proteins deduced from the seven full-length genes identified in the durum wheat cv. Svevo, Table S4: Classification of δ-gliadin genes, identified in different durum and bread wheat cvs., orthoulogous to the Svevo *Gli-δ1a**, *Gli-δ2a** and *Gli-δ1b** genes.

## References

[1] Shewry P.R., Halford N.G. (2002). Cereal seed storage proteins: Structures, properties and role in grain utilization. J. Exp. Bot..

[2] Hausch F., Shan L., Santiago N.A., Gray G.M., Khosla C. (2002). Intestinal digestive resistance of immunodominant gliadin peptides. Am. J. Physiol. Liver Physiol..

[3] Shewry P.R., Tatham A.S. (2016). Improving wheat to remove celiac epitopes but retain functionality. J. Cereal Sci..

[4] Stepniak D., Wiesner M., De Ru A.H., Moustakas A.K., Drijfhout J.W., Papadopoulos G.K., Van Veelen P.A., Koning F. (2008). Large-scale characterization of natural ligands explains the unique gluten-binding properties of HLA-DQ2. J. Immunol..

[5] Adelman D.C., Murray J., Wu T.-T., Mäki M., Green P.H., Kelly C.P. (2018). Measuring Change in Small Intestinal Histology in Patients With Celiac Disease. Am. J. Gastroenterol..

[6] Sollid L.M., Qiao S.-W., Anderson R.P., Gianfrani C., Koning F. (2012). Nomenclature and listing of celiac disease relevant gluten T-cell epitopes restricted by HLA-DQ molecules. Immunogenetics.

[7] Iversen R., Roy B., Stamnæs J., Høydahl L.S., Hnida K., Neumann R.S., Korponay-Szabó I.R., Lundin K.E.A., Sollid L.M. (2019). Efficient T cell–B cell collaboration guides autoantibody epitope bias and onset of celiac disease. Proc. Natl. Acad. Sci. USA.

[8] Dørum S., Steinsbø Ø., Bergseng E., Arntzen M.Ø., de Souza G.A., Sollid L.M. (2016). Gluten-specific antibodies of celiac disease gut plasma cells recognize long proteolytic fragments that typically harbor T-cell epitopes. Sci. Rep..

[9] Shewry P.R., Halford N.G., Lafiandra D. (2003). Genetics of Wheat Gluten Proteins. Adv. Genet..

[10] Wang D.W., Li D., Wang J., Zhao Y., Wang Z., Yue G., Liu X., Qin H., Zhang K., Dong L. (2017). Genome-wide analysis of complex wheat gliadins; the dominant carriers of celi-ac disease epitopes. Sci. Rep..

[11] Qiao S.-W., Bergseng E., Molberg Ø., Xia J., Fleckenstein B., Khosla C., Sollid L.M. (2004). Antigen Presentation to Celiac Lesion-Derived T Cells of a 33-Mer Gliadin Peptide Naturally Formed by Gastrointestinal Digestion. J. Immunol..

[12] Qi P.-F., Wei Y.-M., Ouellet T., Chen Q., Tan X., Zheng Y.-L. (2009). The γ-gliadin multigene family in common wheat (*Triticum aestivum*) and its closely related species. BMC Genom..

[13] Vader W., Kooy Y., van Veelen P., de Ru A., Harris D., Benckhuijsen W., Peña S., Mearin L., Drijfhout J.W., Koning F. (2002). The gluten response in children with celiac disease is directed toward multiple gliadin and glutenin peptides. Gastroenterology.

[14] Camarca A., Anderson R.P., Mamone G., Fierro O., Facchiano A., Costantini S., Zanzi D., Sidney J., Auricchio S., Sette A. (2009). Intestinal T Cell Responses to Gluten Peptides Are Largely Heterogeneous: Implications for a Peptide-Based Therapy in Celiac Disease. J. Immunol..

[15] Kumar B.V.M., Rao U.J.S.P., Prabhasankar P. (2017). Immunogenicity characterization of hexaploid and tetraploid wheat varieties related to celiac disease and wheat allergy. Food Agric. Immunol..

[16] Molberg Ø., Uhlen A.K., Jensen T., Flæte N.S., Fleckenstein B., Arentz–Hansen H., Raki M., Lundin K.E., Sollid L.M. (2005). Mapping of gluten T-cell epitopes in the bread wheat ancestors: Implications for celiac disease. Gastroenterology.

[17] Maccaferri M., Harris N.S., Twardziok S.O., Pasam R.K., Gundlach H., Spannagl M., Ormanbekova D., Lux T., Prade V.M., Milner S.G. (2019). Durum wheat genome highlights past domestication signatures and future improvement targets. Nat. Genet..

[18] Intranet of Durum Wheat Genome Data. https://www.interomics.eu/durum-wheat-genome-intranet.

[19] FGENESH. http://www.softberry.com/berry.phtml?topic=fgenesh&group=programs&subgroup=gfind.

[20] WHEAT URGI. https://wheat-urgi.versailles.inra.fr/.

[21] ExPASy Bioinformatics Resource Portal. http://www.expasy.org.

[22] Gimenez M.J., Pistón F., Atienza S.G. (2010). Identification of suitable reference genes for normalization of qPCR data in comparative transcriptomics analyses in the Triticeae. Planta.

[23] Marcotuli I., Colasuonno P., Blanco A., Gadaleta A., Ilaria M., Pasqualina C., Antonio B., Agata G. (2018). Expression analysis of cellulose synthase-like genes in durum wheat. Sci. Rep..

[24] Bustin S.A., Benes V., Garson J.A., Hellemans J., Huggett J., Kubista M., Mueller R., Nolan T., Pfaffl M.W., Shipley G.L. (2009). The MIQE guidelines: Min-imum information for publication of quantitative real-time PCR experiments. Clin. Chem..

[25] Lafiandra D., Kasarda D.D. (1985). One- and two-dimensional (Two-pH) polyacrilamide gel electrophoresis in a single gel: Separation of wheat proteins. Cereal Chem..

[26] Mejías J., Lu X., Osorio C.E., Ullman J.L., Von Wettstein D., Rustgi S. (2014). Analysis of Wheat Prolamins, the Causative Agents of Celiac Sprue, Using Reversed Phase High Performance Liquid Chromatography (RP-HPLC) and Matrix-Assisted Laser Desorption Ionization Time of Flight Mass Spectrometry (MALDI-TOF-MS). Nutrients.

[27] Anderson O.D., Dong L., Huo N., Gu Y.Q. (2012). A New Class of Wheat Gliadin Genes and Proteins. PLoS ONE.

[28] Wan Y., Shewry P.R., Hawkesford M. (2012). A novel family of γ-gliadin genes are highly regulated by nitrogen supply in developing wheat grain. J. Exp. Bot..

[29] Dong L., Huo N., Wang Y., Deal K., Wang D., Hu T., Dvorak J., Anderson O.D., Luo M.C., Gu Y.Q. (2016). Rapid evolutionary dynamics in a 2.8-Mb chromosomal region containing mul-tiple prolamin and resistance gene families in Aegilops tauschii. Plant J..

[30] Huo N., Zhang S., Zhu T., Dong L., Wang Y., Mohr T., Hu T., Liu Z., Dvorak J., Luo M.-C. (2018). Gene Duplication and Evolution Dynamics in the Homeologous Regions Harboring Multiple Prolamin and Resistance Gene Families in Hexaploid Wheat. Front. Plant Sci..

[31] Anderson O.D., Torres V., Hsia C.C. (2001). The wheat γ-gliadin genes: Characterization of ten new sequences and further understanding of γ-gliadin gene family structure. Theor. Appl. Genet..

[32] Scheets K., Rafalski J., Hedgcoth C., Söll D.G. (1985). Heptapeptide repeat structure of a wheat γ-gliadin. Plant Sci. Lett..

[33] Jang Y.-R., Cho K., Kim S., Sim J.-R., Lee S.-B., Kim B.-G., Gu Y.Q., Altenbach S.B., Lim S.-H., Goo T.-W. (2020). Comparison of MALDI-TOF-MS and RP-HPLC as Rapid Screening Methods for Wheat Lines with Altered Gliadin Compositions. Front. Plant Sci..

[34] Wang S., Shen X., Ge P., Li J., Subburaj S., Li X., Zeller F.J., Hsam S.L., Yan Y. (2012). Molecular characterization and dynamic expression patterns of two types of γ-gliadin genes from Ae-gilops and Triticum species. Theor. Appl. Genet..

[35] Anderson O.D., Huo N., Gu Y.Q. (2013). The gene space in wheat: The complete γ-gliadin gene family from the wheat cultivar Chinese Spring. Funct. Integr. Genom..

[36] Goryunova S.V., Salentijn E.M., Chikida N.N., Kochieva E.Z., van der Meer I.M., Gilissen L.J., Smulders M.J. (2012). Expansion of the Gamma-gliadin gene family in Aegilops and Triticum. BMC Evol. Biol..

[37] De Santis M.A., Cunsolo V., Giuliani M.M., Di Francesco A., Saletti R., Foti S., Flagella Z. (2020). Gluten proteome comparison among durum wheat genotypes with different release date. J. Cereal Sci..

[38] Huo N., Zhu T., Altenbach S., Dong L., Wang Y., Mohr T., Liu Z., Dvorak J., Luo M.-C., Gu Y.Q. (2018). Dynamic Evolution of α-Gliadin Prolamin Gene Family in Homeologous Genomes of Hexaploid Wheat. Sci. Rep..

[39] Hamer R.J., van Vliet T., Shewry P.R., Tatham A.S. (2000). Understanding the structure and properties of gluten: An overview. Wheat Gluten, Proceedings of the 7th International Workshop Gluten 2000, Bristol, UK, 2–6 April 2000.

[40] Shewry P., Tatham A. (1997). Disulphide Bonds in Wheat Gluten Proteins. J. Cereal Sci..

[41] Köhler P., Belitz H.D., Wieser H. (1993). Disulphide bonds in wheat gluten: Further cystine peptides from high molecular weight (HMW) and low molecular weight (LMW) subunits of glutenin and from γ-gliadins. Eur. Food Res. Technol..

[42] Lutz E., Wieser H., Koehler P. (2012). Identification of Disulfide Bonds in Wheat Gluten Proteins by Means of Mass Spectrometry/Electron Transfer Dissociation. J. Agric. Food Chem..

[43] Ferrante P., Masci S., D’Ovidio R., Lafiandra D., Volpi C., Mattei B. (2006). A proteomic approach to verify in vivo expression of a novel γ-gliadin containing an extra cysteine residue. Proteomics.

[44] Gil-Humanes J., Pistón F., Giménez M.J., Martín A., Barro F. (2012). The introgression of RNAi silencing of γ-gliadins into commercial lines of bread wheat changes the mixing and technological properties of the dough. PLoS ONE.

[45] Jouanin A., Schaart J.G., Boyd L.A., Cockram J., Leigh F., Bates R., Wallington E.J., Visser R.G.F., Smulders M.J.M. (2019). Outlook for coeliac disease patients: Towards bread wheat with hypoimmunogenic gluten by gene editing of α- and γ-gliadin gene families. BMC Plant Biol..

[46] Laudencia-Chingcuanco D.L., Stamova B.S., You F.M., Lazo G.R., Beckles D.M., Anderson O.D. (2007). Transcriptional profiling of wheat caryopsis development using cDNA microarrays. Plant Mol. Biol..

[47] Pistón F., Dorado G., Martin A., Barro F. (2006). Cloning of nine γ-gliadin mRNAs (cDNAs) from wheat and the molecular characterization of comparative transcript levels of γ-gliadin sub-classes. J. Cereal Sci..

[48] Miclaus M., Xu J.-H., Messing J. (2011). Differential gene expression and epiregulation of Alpha zein gene copies in maize haplotypes. PLoS Genet..

[49] Wang D., Li F., Cao S., Zhang K. (2020). Genomic and functional genomics analyses of gluten proteins and prospect for simultaneous improvement of end-use and health-related traits in wheat. Theor. Appl. Genet..

[50] Aussenac T., Rhazi L., Fahad S., Basir A. (2018). Storage proteins accumulation and aggregation in developing wheat grains. Global Wheat Production.

