# Supplementary material for: Genome-Wide Identification, Characterization and Expression Pattern Analysis of the γ-Gliadin Gene Family in the Durum Wheat (Triticum durum Desf.) Cultivar Svevo
Source: Genes (Basel). 2021 Oct 29;12(11):1743. doi: 10.3390/genes12111743 (PMC8621147; doi:10.3390/genes12111743)
Supplement: Supplementary file 1 [file genes-12-01743-s001.zip › Supplemetary materials/Figure S1.pdf]

Figure S1. Sequences of the γ-gliadin, δ-gliadin and avenin-like genes reported in Table 1 and their deduced translation products. The stop codons are highlighted in red.

Gli-γ1a

```
      M  K  T  L   L  I  L   T  I  I   A  V  A  L   T  T  T   T  A  N   I  Q  V  D   P  S  G   Q  V  Q   W  P  Q  Q
  1  ATGAAGACCT TACTCATCCT GACAATCATT GCGGTGGCAC TAACTACCAC CACCGCCAAT ATACAGGTCG ACCCTAGTGG CCAAGTACAA TGGCCACAAC
     · Q  Q  P   F  S  Q   P  Q  Q  P   F  S  Q   Q  P  Q   Q  I  F  P   Q  P  Q   Q  T  F   P  H  Q  P   Q  Q  A ·
101  AACAACAACC ATTCTCCCAG CCCCAACAAC CATTCTCCCA ACAACCACAA CAAATTTTTC CCCAACCCCA ACAAACATTC CCCCATCAAC CACAACAAGC
     · F  P  Q   P  Q  Q  T   F  P  H   Q  P  Q   Q  Q  F  P   Q  P  Q   Q  P  Q   Q  Q  F  P   Q  P  Q   Q  P  Q
201  ATTTCCCCAA CCCCAACAAA CATTCCCCCA TCAACCACAA CAACAATTTC CCCAGCCCCA GCAACCACAA CAACAATTTC CCCAGCCCCA ACAACCACAA
      Q  P  F  P   Q  Q  P   Q  Q  Q   F  P  R  P   Q  Q  P   Q  Q  P   F  P  Q  P   Q  Q  P   Q  L  P   F  P  Q  Q
301  CAACCATTTC CCCAGCAACC ACAACAACAA TTTCCCCGGC CCCAACAACC ACAACAACCA TTTCCCCAGC CCCAACAACC CCAACTACCA TTTCCGCAAC
     · P  Q  Q   P  F  P   Q  P  Q  Q   P  Q  Q   P  F  S   Q  L  Q  Q   P  Q  Q   P  L  P   Q  P  Q  Q   S  Q  Q
401  AACCACAACA ACCATTCCCC CAGCCTCAAC AACCCCAACA ACCATTTTCC CAGTTACAGC AACCACAACA ACCTTTACCC CAGCCCCAAC AATCGCAACA
     · P  F  S   Q  Q  Q  Q   S  L  I   Q  P  Y   L  Q  Q  Q   M  N  P   C  K  N   Y  L  L  Q   Q  C  N   P  V  S
501  ACCATTCTCC CAGCAACAAC AATCATTGAT TCAGCCATAC CTACAACAAC AGATGAACCC CTGCAAGAAT TACCTCTTGC AGCAATGCAA CCCTGTGTCA
      L  V  S  S   L  V  S   M  I  L   P  R  S  D   C  Q  V   M  R  Q   Q  C  C  Q   Q  L  A   Q  I  P   Q  Q  L  Q
601  TTGGTGTCAT CCCTCGTGTC AATGATCTTG CCACGAAGTG ATTGCCAGGT GATGCGGCAA CAATGTTGCC AACAACTAGC ACAGATTCCT CAGCAGCTCC
     · C  A  A   I  H  G   V  V  H  S   I  I  M   Q  Q  E   Q  Q  Q  Q   Q  G  I   Q  I  M   R  P  L  F   Q  L  I
701  AGTGCGCAGC CATCCATGGT GTCGTGCATT CCATCATCAT GCAGCAAGAA CAACAACAAC AACAAGGCAT ACAGATCATG CGGCCACTTT TCCAGCTCAT
     · Q  G  Q   G  I  I  Q   P  Q  Q   P  A  Q   L  E  V  I   R  S  L   V  L  G   T  L  P  T   M  C  N   V  F  V
801  CCAGGGTCAG GGCATCATCC AACCTCAACA ACCAGCTCAA TTGGAGGTGA TCAGGTCATT GGTATTGGGA ACTCTTCCAA CCATGTGCAA TGTATTTGTT
      P  P  E  C   S  T  T   K  A  P   F  A  S  I   V  A  D   I  G  G   Q
901  CCACCTGAGT GCTCCACCAC CAAGGCACCA TTTGCCAGCA TAGTTGCCGA CATTGGTGGC CAATGA
```

*Gli-γ2a**

```
      M  K  T  L   L  I  L   T  I  F   A  A  A  L   T  I  A   T  A  N   I  Q  V  D   P  S  G   Q  V  Q   W  P  Q  Q
  1  ATGAAGACCT TACTCATCCT CACAATCTTT GCAGCGGCTC TAACCATCGC CACCGCCAAT ATACAGGTCG ACCCTAGCGG CCAAGTACAA TGGCCACAAC
     · Q  P  F   P
101  AACAACCATT CCCCTAGCCC CAACCATTCT CCCAGCAACC ACAACAAGCG TTTCCCCAAC CCCAACAAAC ATTCCCCCTT CAACCACAGC AAGTATTTCC
201  CCAACCACAA CAACAATTTC CCCAGCCCCA GCAACCACAA CAACCATTTC CCCAGCCCCA ACAACCCCAA CAACCATTCC CCCAGCCTCA ACAACCCCAA
301  CAACCATTTC CCCAGTCACA GCAACCACAA CAACCTTTTC CCCAGCCCCA ACAACAATTC CCGCAGCCCC AACAACCGCA ACAATCATTC CCCCAGCAGC
401  AACAACTGTT GATTCAGCCA TATCTACAAC AACAGATGAA CCCCTGCAAG AATTACCTCT TGCAGCAATG CAACCCCGTG TCATTAGTGT CGTCCCTCGT
501  GTCAATGATC TTGCCACGAA GTGATTGCCA GGTGATGCAG CAACAATGTT GCCAACAACT AGCACAGATT CCTCGTCAGC TCCAGTGCAC AGCCATCCAT
601  AGCGTGGTGC ATGCCATCAT CATGCAGCAA GAACAACAAG GCATACAGAT CCTGCGACCA CTGTTTCAGC TTGTCCAAGG ACAGGGCATC ATCCAACCTC
701  AACAACCAGC TCAATATGAG GTGATCAGGT CATTGGTATT GAGAACCCTT CCAAACATGT GCAACGTGTA TGTCCGACCT GACTGCTCCA CCATCAACGC
801  ACCATTTGCC AGCATAGTTG CCGGCATTGG TGGCCAATGA
```

Gli-γ3a

M K T L F I L T I L A M A T T I A T A N M Q V D P S G Q V Q W P Q Q
1 ATGAAGACCT TATTCATCCT AACAATCCTT GCGATGGCAA CAACTATCGC CACCGCGAAT ATGCAGGTCG ACCCCAGCGG CCAAGTACAA TGGCCACAAC
· Q P F R Q P Q Q P F Y Q Q P Q Q T F P Q P Q Q T F P H Q P Q Q Q F
101 AACAACCATT CCGCCAGCCC CAACAACCAT TCTACCAGCA ACCACAACAA ACATTTCCCC AACCCCAACA AACATTCCCC CATCAACCAC AACAACAATT
· P Q P Q Q P Q Q Q F P Q P Q Q P Q Q P F P Q P Q Q A Q L P F P Q Q
201 TCCCCAGCCC CAGCAACCAC AACAACAATT TCCGCAGCCC CAGCAACCAC AACAACCATT TCCCCAACCC CAACAAGCCC AACTACCATT TCCTCAACAA
P Q Q P F P Q P Q Q P Q Q P F P Q S Q Q P Q Q P F P Q P Q Q P Q Q S
301 CCACAACAAC CATTCCCCCA GCCTCAACAA CCCCAACAAC CATTTCCCCA GTCACAGCAA CCACAACAAC CTTTTCCCCA GCCCCAACAA CCGCAACAAT
· F P Q Q Q Q P L I Q P Y L Q Q Q M N P C K N Y L L Q Q C N P V S L
401 CATTCCCCCA GCAACAACAA CCGTTGATTC AGCCATATCT ACAACAACAG ATGAACCCTT GCAAGAATTA CCTCTTACAG CAATGCAACC CTGTGTCATT
· V S S L V S M I L P R S D C Q V M Q Q Q C C Q Q L A Q I P R Q L Q
501 GGTGTCATCC CTCGTGTCAA TGATCTTGCC ACGAAGTGAT TGCCAGGTGA TGCAGCAACA ATGTTGCCAA CAACTAGCAC AGATTCCTCG CCAGCTCCAG
C A A I H S V V H S I V M Q Q E Q Q Q G I Q I L R P L F Q L I Q G Q
601 TGTGCAGCCA TCCATAGCGT CGTGCATTCC ATCGTCATGC AGCAAGAACA ACAACAAGGC ATACAGATCC TCCGGCCACT GTTTCAGCTC ATCCAAGGTC
· G I I Q P Q Q P A Q Y E V I R S L V L R T L P N M C N V Y V R P D
701 AGGGCATCAT CCAACCTCAA CAACCAGCTC AATATGAGGT GATCAGGTCA TTGGTATTGA GAACCCTTCC AAACATGTGC AACGTGTATG TCCGACCTGA
· C S T I N A P F A S I V A G I S G Q
801 CTGCTCCACC ATCAACGCAC CATTTGCCAG CATAGTCGCC GGCATCAGTG GACAATGA

Gli-γ4a

M K T L F I L T I L A M A T T I A T A N M Q V D P S G Q V Q W P Q Q
1 ATGAAGACCT TATTCATCCT AACAATCCTT GCGATGGCAA CAACTATCGC CACCGCGAAT ATGCAGGTCG ACCCCAGCGG CCAAGTACAA TGGCCACAAC
· Q P F R Q P Q Q P F Y Q Q P Q Q T F P Q P Q Q I F P H Q P Q Q Q F
101 AACAACCATT CCGCCAGCCC CAACAACCAT TCTACCAGCA ACCACAACAA ACATTTCCCC AACCCCAACA AATATTCCCC CATCAACCAC AACAACAATT
· P Q P Q Q P Q Q Q F P Q P Q Q P Q Q P F P Q P Q Q A Q L P F P Q Q
201 TCCCCAGCCC CAACAACCAC AACAACAATT TCCGCAGCCC CAGCAACCAC AACAACCATT TCCCCAGCCC CAACAAGCCC AACTACCATT TCCTCAACAA
P Q Q P F P Q P Q Q P Q Q P F P Q S Q Q P Q Q P F P Q P Q Q P Q Q S
301 CCACAACAAC CATTCCCCCA GCCTCAACAA CCCCAACAAC CATTTCCCCA GTCACAGCAA CCACAACAAC CTTTTCCCCA GCCCCAACAA CCGCAACAAT
· F P Q Q Q Q P L I Q P Y L Q Q Q M N P C K N Y L L Q Q C N P V S L
401 CATTCCCCCA GCAACAACAA CCGTTGATTC AGCCATATCT ACAACAACAG ATGAACCCTT GCAAGAATTA CCTCTTACAG CAATGCAACC CTGTGTCATT
· V S S L V S M I L P R S D C Q V M Q Q Q C C Q Q L A Q I P R Q L Q
501 GGTGTCATCC CTCGTGTCAA TGATCTTGCC ACGAAGTGAT TGCCAGGTGA TGCAGCAACA ATGTTGCCAA CAACTAGCAC AGATTCCTCG CCAGCTCCAG
C A A I H S V V H S I I M Q Q E Q Q Q G I Q I L R P L F Q L V Q G Q
601 TGTGCAGCCA TCCATAGCGT CGTGCATTCC ATCATCATGC AGCAAGAACA ACAACAAGGC ATACAGATCC TCCGGCCACT GTTTCAGCTC GTCCAAGGTC
· G I I Q P Q Q P A Q Y E V I R S L V L R T L P N M C N V Y V R P D
701 AGGGCATCAT CCAACCTCAA CAACCAGCTC AATATGAGGT GATCAGGTCA TTGGTATTGA GAACCCTTCC AAACATGTGC AACGTGTATG TCCGACCTGA
· C S T I N A P F A S I V A G I S G Q
801 CTGCTCCACC ATCAACGCAC CATTTGCTAG CATAGTCGCC GGCATCAGTG GACAATGA

Gli-γ1b

```
     M  K  T  L   L  I  L   T  I  L   A  M  A  T   T  I  A   T  A  N   M  Q  V  D   P  S  G   Q  V  Q   W  P  Q  Q
  1 ATGAAGACCT TACTCATCCT AACAATCCTT GCGATGGCAA CAACCATCGC CACCGCCAAT ATGCAAGTCG ACCCCAGCGG CCAAGTACAA TGGCCACAAC
     ·  Q  P  F   P  Q  P   Q  Q  P  F   C  E  Q   P  Q  R   T  I  P  Q   P  H  Q   T  F  H   H  Q  P  Q   Q  T  F
101 AACAACCATT CCCCCAGCCC CAACAACCAT TCTGCGAGCA ACCACAACGA ACTATTCCCC AACCCCATCA AACATTCCAC CATCAACCAC AACAAACATT
     · P  Q  P   E  Q  T  Y   P  H  Q   P  Q  Q   Q  F  P  Q   T  Q  Q   P  Q  Q   P  F  P  Q   P  Q  Q   T  F  P
201 TCCCCAACCC GAACAAACAT ACCCCCATCA ACCACAACAA CAATTTCCCC AGACCCAACA ACCACAACAA CCATTTCCCC AGCCCCAACA AACATTCCCC
     Q  Q  P  Q   L  P  F   P  Q  Q   P  Q  Q  P   F  P  Q   P  Q  Q   P  Q  Q  P   F  P  Q   S  Q  Q   P  Q  Q  P
301 CAACAACCCC AACTACCATT TCCCCAACAA CCCCAACAAC CATTCCCCCA GCCTCAGCAA CCCCAACAAC CATTTCCCCA GTCACAACAA CCACAACAAC
     ·  F  P  Q   P  Q  Q   Q  F  P  Q   P  Q  Q   P  Q  Q   S  F  P  Q   Q  Q  Q   P  A  I   Q  S  F  L   Q  Q  Q
401 CTTTTCCCCA GCCCCAACAA CAATTTCCGC AGCCCCAACA ACCACAACAA TCATTCCCCC AACAACAACA ACCGGCGATT CAGTCATTTC TACAACAACA
     · M  N  P   C  K  N  F   L  L  Q   Q  C  N   H  V  S  L   V  S  S   L  V  S   I  I  L  P   R  S  D   C  Q  V
501 GATGAACCCC TGCAAGAATT TCCTCTTGCA GCAATGCAAC CATGTGTCAT TGGTGTCATC TCTCGTGTCA ATAATTTTGC CACGAAGTGA TTGCCAGGTG
     M  Q  Q  Q   C  C  Q   Q  L  A   Q  I  P  Q   Q  L  Q   C  A  A   I  H  S  V   A  H  S   I  I  M   Q  Q  E  Q
601 ATGCAGCAAC AATGTTGCCA ACAACTAGCA CAAATTCCTC AACAGCTCCA GTGCGCAGCC ATCCACAGCG TCGCGCATTC CATCATCATG CAACAAGAAC
     ·  Q  Q  G   V  P  I   L  R  P  L   F  Q  L   A  Q  G   L  G  I  I   Q  P  Q   Q  P  A   Q  L  E  G   I  R  S
701 AACAACAAGG CGTGCCGATC CTGCGGCCAC TATTTCAGCT CGCCCAGGGT CTGGGTATCA TCCAACCTCA ACAACCAGCT CAATTGGAGG GGATCAGGTC
     · L  V  L   K  T  L  P   T  M  C   N  V  Y   V  P  P  D   C  S  T   I  N  V   P  Y  A  N   I  D  A   G  I  G
801 ATTGGTATTG AAAACTCTTC CAACCATGTG CAACGTGTAT GTGCCACCTG ACTGCTCCAC CATCAACGTA CCATATGCCA ACATAGACGC TGGCATTGGT
     G  Q
901 GGCCAATGA
```

Gli-γ2b

```
     M  K  T  L   L  I  L   T  I  L   A  M  A  T   I  I  A   T  A  N   M  Q  V  D   P  S  S   R  V  Q   W  P  Q  E
  1 ATGAAGACCT TACTCATCCT AACAATCCTT GCGATGGCAA CAATAATCGC CACTGCCAAT ATGCAGGTCG ACCCTAGCAG CCGAGTACAA TGGCCACAAG
     ·  Q  P  S   P  Q  S   Q  Q  P  F   S  Q  Q   P  Q  Q   I  F  P  Q   P  Q  Q   T  L  P   H  Q  P  Q   Q  A  F
101 AACAACCATC CCCCCAGTCC CAACAACCAT TCTCCCAGCA ACCACAACAA ATATTTCCCC AACCCCAACA AACATTGCCC CATCAACCAC AACAAGCATT
     · P  Q  P   Q  Q  T  F   P  H  R   P  Q  Q   Q  F  P  Q   P  Q  Q   P  Q  Q   P  F  P  Q   P  Q  Q   P  Q  L
201 TCCCCAACCT CAACAAACAT TCCCCCATCG ACCACAACAA CAATTTCCCC AGCCCCAGCA ACCACAACAA CCATTTCCTC AGCCCCAACA ACCCCAACTA
     P  F  P  Q   Q  T  Q   Q  P  F   P  Q  P  Q   Q  P  Q   Q  P  F   P  Q  S  Q   Q  P  Q   Q  P  F   P  Q  P  Q
301 CCATTTCCCC AACAAACACA ACAACCATTC CCCCAGCCTC AACAACCCCA ACAACCATTT CCCCAGTCAC AGCAACCACA ACAACCTTTT CCCCAGCCCC
     ·  Q  Q  F   P  Q  P   Q  Q  P  Q   Q  S  F   P  Q  Q   Q  Q  W  M   I  Q  S   F  L  Q   Q  Q  M  N   P  C  K
401 AACAACAATT TCCGCAGCCC CAACAACCAC AACAATCATT CCCCCAACAA CAACAATGGA TGATTCAGTC ATTTCTACAA CAACAGATGA ACCCCTGCAA
     · N  F  L   L  Q  Q  C   N  P  V   S  L  V   S  S  L  V   S  I  I   L  P  R   S  D  C  Q   L  M  Q   Q  Q  C
501 GAATTTCCTC TTGCAGCAAT GCAACCCTGT GTCATTGGTG TCATCTCTCG TGTCAATAAT CTTGCCACGA AGTGATTGCC AGCTGATGCA GCAACAATGT
     C  Q  Q  L   A  Q  I   P  Q  Q   L  Q  C  A   A  I  H   S  V  A   H  S  I  V   M  Q  Q   E  Q  Q   R  G  V  Q
601 TGCCAACAAC TAGCACAAAT TCCTCAACAG CTCCAGTGCG CAGCCATCCA CAGCGTCGCG CATTCCATCG TCATGCAGCA AGAACAACAA CGAGGCGTGC
     ·  I  L  R   P  L  F   Q  L  A  Q   G  L  G   I  I  Q   P  Q  Q  P   A  Q  L   E  G  I   R  S  L  V   L  K  T
701 AGATCCTGCG GCCACTATTT CAGCTCGCCC AGGGTCTGGG TATCATCCAA CCTCAACAAC CAGCTCAATT GGAGGGGATC AGGTCATTGG TATTGAAAAC
     · L  P  T   M  C  N  V   Y  V  P   P  D  C   S  T  I  N   V  P  Y   A  S  I   D  A  V  I   G  G  Q
801 TCTTCCAACC ATGTGCAATG TGTATGTCCC ACCTGACTGC TCCACCATCA ACGTGCCATA TGCCAGCATA GACGCTGTCA TTGGTGGCCA ATGA
```

Gli-γ3b

```
     M  K  T  L   L  I  L   T  I  L   A  M  A  I   T  I  S   T  A  N   M  Q  V  D   P  S  G   Q  V  Q   W  P  Q  Q
  1 ATGAAGACCT TACTCATCCT GACGATCCTT GCGATGGCAA TAACCATCAG CACCGCCAAT ATGCAGGTCG ACCCTAGTGG CCAAGTACAA TGGCCACAAC
     · Q  L  V   P  Q  P   Q  Q  P  L   S  Q  Q   P  Q  Q   A  F  P  Q   P  Q  Q   T  F  P   H  Q  P  Q   Q  Q  V
101 AACAACTAGT CCCCCAACCC CAACAGCCAT TATCCCAGCA ACCGCAACAA GCATTTCCCC AACCCCAACA AACATTTCCC CATCAACCAC AACAACAAGT
     · P  Q  P   Q  Q  P  Q   Q  P  F   L  Q  P   Q  Q  A  F   P  Q  Q   P  Q  Q   P  F  P  Q   T  Q  Q   P  Q  Q
201 TCCCCAGCCT CAGCAACCAC AACAACCATT TCTCCAGCCC CAACAAGCAT TCCCCCAACA ACCACAACAA CCATTCCCTC AGACTCAACA ACCACAACAA

     P  F  P  Q   Q  P  Q   Q  P  F   P  Q  T  Q   Q  P  Q   Q  P  F   P  Q  Q  P   Q  Q  P   F  P  Q   T  Q  Q  P
301 CCATTTCCCC AGCAACCACA ACAACCATTC CCCCAGACTC AACAACCACA ACAACCATTT CCCCAGCAAC CACAACAACC ATTCCCCCAG ACTCAACAAC
     · Q  Q  P   F  P  Q   F  Q  Q  P   H  Q  P   F  P  Q   P  Q  Q  Q   F  P  Q   P  Q  Q   P  Q  Q  S   F  L  Q
401 CACAACAACC ATTTCCCCAG TTCCAGCAAC CACACCAACC TTTTCCCCAG CCCCAACAAC AATTCCCGCA GCCCCAACAA CCGCAACAAT CATTCCTCCA
     · Q  Q  R   P  F  I  Q   P  S  L   Q  Q  R   L  N  P  C   K  N  I   L  L  Q   Q  C  K  P   A  S  L   V  S  S
501 GCAACAACGA CCATTCATTC AGCCATCTCT ACAACAACGT TTGAACCCAT GCAAGAATAT CCTCTTGCAA CAATGCAAAC CTGCGTCATT GGTGTCATCC
     L  W  S  I   I  W  P   Q  S  D   C  Q  V  M   Q  Q  Q   C  C  Q   E  L  A  Q   I  P  Q   Q  L  Q   C  A  A  I
601 CTCTGGTCGA TAATCTGGCC ACAAAGCGAT TGCCAAGTGA TGCAGCAACA ATGCTGCCAA GAACTAGCAC AGATTCCTCA GCAGCTCCAG TGCGCAGCCA
     · H  S  V   V  H  S   I  I  M  Q   Q  Q  Q   Q  Q  Q   Q  Q  Q  Q   Q  Q  Q   G  M  H   I  L  L  T   L  S  Q
701 TCCATAGCGT CGTGCATTCC ATCATCATGC AGCAGCAACA ACAACAACAA CAACAACAAC AACAACAACA AGGCATGCAT ATCCTGCTGA CACTATCTCA
     · Q  Q  Q   L  G  Q  G   T  L  V   Q  G  Q   G  I  I  Q   P  Q  Q   L  A  Q   L  E  A  I   R  S  L   V  L  Q
801 ACAACAACAG TTGGGTCAAG GTACTCTCGT CCAAGGCCAG GGCATCATCC AACCTCAACA ACTAGCTCAA TTGGAGGCGA TCAGGTCATT GGTGTTGCAA
     T  L  P  T   M  C  N   V  Y  V   P  P  E  C   S  I  I   R  A  P   F  A  S  I   V  A  G   I  G  G   Q>
901 ACTCTTCCAA CCATGTGCAA CGTGTATGTC CCACCTGAGT GCTCCATCAT CAGGGCACCA TTTGCCAGCA TAGTCGCGGG GATTGGTGGC CAATGA
```

Gli-γ4b*

```
     M  K  T  L   L  I  L   T  I  L   A  M  A  V   T  I  G   T  A  N   M  Q  V  G   P  S  G   Q  V  Q   W  P  Q  Q
  1 ATGAAGACCT TACTCATCCT AACAATCCTT GCGATGGCAG TAACCATCGG CACCGCCAAT ATGCAGGTCG GCCCTAGCGG CCAAGTACAA TGGCCACAAC
     · Q  P  V   L  L  P   Q  Q  P  F   S  Q  Q   P  Q  Q   T  F  P  Q   P  Q  Q   T  F  P   H  Q  P  Q   Q>
101 AACAACCAGT CCTGCTGCCT CAGCAACCAT TCTCCCAGCA ACCACAACAA ACATTTCCCC AACCCCAACA AACATTCCCC CATCAACCAC AACAATAATT
201 TCCCCAGCCT CAACAACCAC AACAACAATT TCTCCAGCCA CAACAACCAT TCCCCCAACA ACCACAACAA CCATATCCCC AACAACCACA ACAACCATTC
301 CCCCAGACTC AACAACCCCA ACAACTATTT CCCCAGTCCC AGCAACCACA ACAACCATAT CCCCAGCAAC CACAACAACC ATTCCCCCAG ACTCAACAAC
401 CCCAACAACA ATTTCCCCAA TCCCAGCAAC CACAACAACC ATTTCCCCAA CCCCAACAAC TGCAACAATC ATTCCCCCAG CAACAACCAT CGTTCATTCA
501 GCCATCTCTA CAACAACTGC TGAACCCATG CAAGAATTTA CTCTTGCAGC AATGTAGACC TGTGTCATTG GTCTCATCCC TCTGGTCAAT GATCTGGCCA
601 CAAAGCGCTT GCCAAGTGAT GAGGAAACAA TGCTGCCAAC AACTAGCACA GATTCCTCAG CAGCTCCAGT GTGCAGCCAT CCATAGCGTC GTGCATTCCA
701 TCAGCATGTA GCAAGAACAA CAACAACAAC AACAACAACA ACAACAACAA CAACAACAAC AACAACAACA ACAAGGCATG CGTATCCTGC TGCCACTATA
801 TCAGCAACAA CAGGTGGGTC AAGGTACTCT CGTCCAGGGC CAGGGCATCA TCCAACCCCA ACAACCAGCT CAATTGGAGG CGATCAGGTC ATTGGTGTTG
901 CAAACTCTTC CAACCATGTG CAACGTGTAT GTCCCACCTG AGTGCTCTAT CATCAAGGCA CCATTTGCCA GCATAGTCAC CGGAATTGGT GGCCAATGA
```

Gli-γ5b

```
      M  K  T  L  L  I  L  T  I  L  A  M  A  I  T  I  A  T  A  N  M  Q  V  D  P  S  G  Q  V  Q  W  P  Q  Q
  1   ATGAAGACCT TACTCATCCT GACAATCCTT GCGATGGCAA TAACCATCGC CACCGCCAAT ATGCAGGTCG ACCCTAGCGG CCAAGTACAA TGGCCGCAAC
      · Q  P  F  L  Q  P  H  Q  P  F  S  Q  Q  P  Q  Q  I  F  P  Q  P  Q  Q  T  F  P  H  Q  P  Q  Q  Q  F
101   AACAACCATT CCTGCAGCCT CACCAACCAT TCTCCCAGCA ACCACAACAA ATATTTCCCC AACCCCAACA AACATTCCCC CATCAACCAC AACAACAATT
      · P  Q  P  Q  Q  P  Q  Q  Q  F  L  Q  P  R  Q  P  F  P  Q  Q  P  Q  Q  P  Y  P  Q  Q  P  Q  Q  P  F
201   TCCCCAGCCT CAGCAACCAC AACAACAATT TCTCCAGCCC CGACAACCAT TCCCCCAACA ACCACAACAA CCATATCCCC AGCAACCACA ACAACCGTTC
      P  Q  T  Q  Q  P  Q  Q  P  F  P  Q  S  K  Q  P  Q  Q  P  F  P  Q  P  Q  Q  P  Q  Q  S  F  P  Q  Q  Q
301   CCCCAGACTC AACAACCCCA ACAACCATTT CCCCAGTCCA AGCAACCACA ACAACCTTTT CCCCAGCCCC AACAACCGCA ACAATCATTC CCCCAACAAC
      · P  S  L  I  Q  Q  S  L  Q  Q  Q  L  N  P  C  K  N  F  L  L  Q  Q  C  K  P  V  S  L  V  S  S  L  W
401   AACCATCATT GATTCAACAA TCTCTACAAC AACAGTTGAA CCCATGCAAG AATTTCCTCT TGCAGCAATG CAAACCTGTG TCCTTGGTGT CATCCCTCTG
      · S  I  I  L  P  P  S  D  C  Q  V  M  R  Q  Q  C  C  Q  Q  L  A  Q  I  P  Q  Q  L  Q  C  A  A  I  H
501   GTCAATCATC TTGCCACCAA GCGATTGCCA GGTGATGCGG CAACAATGTT GTCAACAACT AGCACAAATT CCTCAGCAAC TCCAGTGTGC AGCCATCCAT
      S  V  V  H  S  I  I  M  Q  Q  E  Q  Q  E  Q  L  Q  G  V  Q  I  L  V  P  L  S  Q  Q  Q  Q  V  G  Q  G
601   AGCGTCGTGC ATTCCATCAT CATGCAGCAA GAACAACAAG AACAACTACA GGGTGTGCAA ATCCTGGTGC CACTGTCTCA ACAGCAACAG GTGGGTCAAG
      · I  L  V  Q  G  Q  G  I  I  Q  P  Q  Q  P  A  Q  L  E  V  I  R  S  L  V  L  Q  T  L  P  T  M  C  N
701   GTATTCTCGT CCAGGGTCAA GGCATCATCC AACCTCAACA ACCAGCTCAA TTGGAGGTGA TCAGGTCATT GGTGTTGCAA ACTCTTCCAA CCATGTGCAA
      · V  Y  V  P  P  Y  C  S  T  I  R  A  P  F  A  S  I  V  A  S  I  G  G  Q
801   CGTGTATGTC CCACCTTACT GCTCCACCAT CAGGGCACCA TTTGCTAGCA TAGTCGCCAG CATTGGTGGC CAATGA
```

*Gli-δ1a**

```
     M  K  I  F   L  V  F   A  L  L   V  V  S  T   I  I  T   T  A  T   V  Q  L  D   P  S  I   H  V  Q   E  R  P  Q
  1 ATGAAGATCT TCTTGGTCTT TGCCCTCCTC GTTGTATCAA CGATCATCAC CACCGCGACC GTGCAGCTCG ACCCTAGCAT CCATGTACAA GAAAGGCCAC
     ·  Q  S  F   P  Q  Q   Q  P  L  N   Q  Q  Q   P  F  P   L  Q  E  P   Q  Q  P   L  F  Q   Q  Q  Q  P   Y  P  Q
101 AACAATCATT TCCACAGCAG CAACCACTTA ATCAACAACA ACCATTCCCG CTGCAAGAGC CACAACAACC ACTATTCCAG CAACAACAAC CGTATCCACA
     · Q  S  L   P  Q  Q  Q   L  P  Q   Q  H  L   F  P
201 ACAGTCACTT CCCCAACAAC AACTTCCCCA GCAACATTTA TTTCCGTAGC AACCGCCGCA ACAACAATTT CCACAGCAGA TGCCACTTCC GTATCAACAA
301 CAAATATTCC CGCAACAACA ACAACCCCCA CAACAACAAC CATTTTACCA ATATCAACAA CCATTAACAC AACAACCATA CCCGCAAGAG CAACCATTGC
401 CACAACAACA ACCTTCTGTG GAGGAAAACC AACAATTGAA CTTGTGCAAG GAGTTCCTGC TGCAGCAGTG CAACCCGGAG GAGAAACTGT CATTACTGCA
501 GTCAGTGATC CCGTTCCTCC GACCAAAGAC CTCGCAACAG AACAACTGCC AGTTGAAGCG GCAACAATGT TGTCGACAAC TTGCACATAT CAGCGAGCCG
601 TCCCGATGCC CGACCATCCA CAACATTGTG CACGCCATCA TTATGCAACA ACAACAACAA CAACAACAAC AACAACAACA TGTGGATAGA GGTTTTGTCC
701 AGCCTCAACC ACAACAGTTG GGCCAGGGAA TGCCCATGCA GCCTCAACAT CAATTGGGCC AGGGCTTAAG CCTACCTCAA CAACTAGCCC AGTTCAAGTT
801 GGTTAGGTTA CTTGTGATTC AGACCTTGCC TATGTTATGC AATGTGCATG TCCCATCTGA TTGCTACACC ATTAGTGCAC CATTTGGTGG CATCACTGCC
901 TACAACAGTG GACAATGA
```

*Gli-δ2a**

```
     M  K  I  F   L  V  F   A  L  L   V  A  T  T   I  S  T   T  G  T   A  Q  L  D   P  S  V   H  D  L   E  R  P  H
  1 ATGAAGATCT TCTTGGTCTT TGCCCTCCTG GTTGCCACAA CGATCAGCAC CACCGGCACT GCGCAGCTCG ACCCTAGCGT CCATGACTTA GAAAGGCCAC
     ·  Q  S  F   P  Q  Q   Q  P  L  P   P  L  Q   P  F  P   P  Q  E  P
101 ACCAATCATT TCCACAACAG CAGCCACTTC CCCCGCTACA ACCATTCCCG CCGCAAGAGC CATAACAACC ATTTCCGTTG CAGCAACCAC AACACCTGCA
201 ACAACAACCA CTTCCCCAAC AACATCTATT TCCGCAGCAA CCGCCACAAC AACAATTTCC ACAGCAACAA CAAACATTCC CGCAACCTCA TCAACAACCA
301 CAATTCCCGA AACAACAACC ATTTTACCAA TATCAACAAC CATTTACACA ACAACCATAA CCGAAAGAGC AACCATTGGC ACAGCAACAA CCTTGTGTAG
401 AGGAAAGACA ACGACTGAAC TTGTGCAAGG AATTCCTCTC GCAGCAGTGC AACCCGGAGG AGAAGGTGTC GTCACTCCAT TCGGTGATCC CGTTCCTCTG
501 ACCAAAGACC TCGCAACAGA ATAGCTGCCA GTTGAAGCGA CAAGAATGTT GTCGCCAACT TGCACATATC CGTGAGCAGT CTTGATGCCC GGCCATCCAA
601 AGCCCTGTGC ATGCCATCAT TGTGCAACAA CAACAACGAC GACAACAACA ACAGGTTGAT AGAGGTTTCG GCCAGCCTCA ACCCACCACA GTTGGGCCAG
701 GGAATGCCCA TGCAACCTCA ATAGCCACTG GGCCAGGGCT TTATCCTACC TCAGCAACTA GCCCAGTTTA AGTTGGTTAG GTCACTTGCG ATTCAGACCC
801 TGCCTATGTT ATGCAACGTG CATGTCCCGT CTGATTGCTA CACCATTACT GCATATTTTG GTAGCATGAC TGCCTGCAGC TGTGGACAAT GA
```

*Gli-δ1b**

```
     M  K  I  F   L  V  F   A  L  L   V  V  S  T   T  I  T   T  A  I   V  Q  L  D   P  S  V   H  V  Q   E  R  P  Q
  1 ATGAAGATCT TCTTGGTCTT TGCCCTCCTC GTTGTATCAA CGACCATCAC CACCGCGATC GTGCAGCTCG ACCCTAGTGT CCATGTCCAA GAAAGGCCAC
     ·  Q  S  F   P  Q  Q   Q  P  L  T   Q  Q  Q   P  F  P   L  Q  E  P   Q  Q  P   L  F  P   Q  K  E  P   Q  Q  P
101 AACAATCATT TCCGCAGCAG CAACCACTTA CCCAGCAACA ACCATTCCCG CTGCAAGAAC CACAACAACC ACTATTCCCG CAAAAAGAGC CACAACAACC
     · F  L  L   Q  Q  P  Q   P  Q  E   Q  Q  P   Y  P  Q  Q   P  L  L   Q  Q  Q   L  P
201 ATTTTTGCTG CAGCAACCAC AACCCCAGGA ACAACAACCA TATCCACAAC AGCCACTTCT CCAACAACAA CTTCCCTAGC AACATCTATT CCCGCAGCAG
301 CCACCACAAC AACAATTTCC GCAGCAGATG CCACTTCCAT ATGAACAACA AACATTCCCG CTACAACAGC AACGGCAACC ACAATTCCCG CAACCACAAC
401 CATTTCCCCA ATATCAACAA CCATTAACAC AACAACCATA CCCGCAAGAG CAACCATTGC CACAACAACA ACCTTCTATG GAGGAAAAAC AACAATTGAA
501 CGTGTGCAAG GAGTTCCTCC TGCAACAGTG CAACCCGGAG GAGAAACTGT CATTACTCCA GTCAGTGATC CCGTTCCTCC GACCAAAGAC CTCGCAACAG
601 AATAGCTGCC AGTTGAAGCG TCTACAATGT TGTCAACAAC TTGCACATAT CAATGAACCG TCCCGATGCC CGGCCATCCA CAACATTGTG CACGCCATCG
701 TCGTTCAACA ACAACATGTG GATAGAGGTT TCGGCCAGCC TCAACCACAA CAGTTGGGCC AGGGAATGCC CATGCAGCCT CAATATCAAT TGGGCCAGGG
801 CTTTATCCTA CCTCAACAAC TAGCCCAGTT CAAGTTGGTT AGGTTACTTG TGATTCAGAC CCTGCCTATG TTATGCAACG TGCATGTCCC ATCTGATTGC
901 TACACCACTA GTGCACCATC TGGTAGCATG ACTGCCTTGA ACGGTGGACA GTGA
```

Av-la*

```
     M  K  I  F   L  A  L   A  F  L   A  A  M  A   T  I  A   I  V  T   T  D  M  K   L  P  Q   L  Q  P   Q  K  S  F
  1 ATGAAGATCT TCCTTGCGCT TGCCTTTCTT GCTGCTATGG CTACCATCGC CATAGTCACC ACCGATATGA AACTACCCCA GTTGCAACCA CAAAAATCAT
     · S  Q  Q   Q  P  F   P  Q  P  Q   L  L  F   R  E  E   Q  S  A  P   Q  Q
101 TTTCACAACA ACAACCCTTT CCACAACCAC AACTACTCTT TCGAGAGGAG CAATCGGCTC CACAACAATA GCCATTTCCA CCACAACAAT TACCATTTGG
201 GCAACAACAA TTTCCACAAC CACCACCCTA TCCCCCACCA CAACAACCAC TCTATCCCCA ACCACAACAA CAACCATTCC CACAACAACA TGAACAACCA
301 GCTCAGGAAC AACCATTTGT ACAACAATTG AACCCCATGT AGGGATGTAT TCCTACAACA GTGTAATCCA GTGACAACTT TATCATTTCT CCAGTCAGAG
401 GTGGTGCAAC AAAGCAGATG TCAGGTGTTA AGGAAGCAAT GTTGTCAGCA ACTGTCATGT ATTCCCAAGC AATCACAGTG CCATGCCATC CACAATGTTG
501 CGCACGCCAT TATTTTGCAA CAACGACAAC AATTGGGAGA AAGGTACTTT GGTCCAACCT CAACAACAAC TACTAGGTCG AGGTTCCATC CAACCTCAAC
601 AACATCGGTT GAATCAGGGT TGGATCGACA CAATAAGGAC ATGGGTGCTT CAGACTCTAC CGACAATGTG CGACATCCAC GTCACACTGT ACTGCTCTAT
701 CACCTCATCA GCATAGCAAC GTCACTATAT ATACTAGCAT GAGTGGCTAA TGA
```

Av-2a

```
     M  K  K  F   L  V  L   A  L  I   V  V  A  A   T  S  T   A  A  E   P  F  T  A   F  R  A   A  W  E   P  H  H  P
  1 ATGAAGAAAT TCCTCGTCCT TGCCCTCATT GTTGTCGCGG CGACCTCCAC GGCCGCCGAA CCCTTTACTG CATTCCGTGC TGCTTGGGAG CCGCACCATC
     · S  S  P   E  Q  Q   P  T  P  Q   P  Q  E   Q  P  V   P  H  Q  K   L  N  P   C  R  D   A  L  L  Q   Q  C  S
101 CATCATCTCC AGAGCAGCAA CCAACTCCAC AGCCACAAGA ACAACCAGTT CCACATCAAA AGTTGAACCC ATGCAGGGAT GCCCTCCTAC AACAGTGTAG
     · P  V  A   D  M  S  F   L  R  S   Q  A  V   Q  P  R  S   C  L  V   M  W  E   Q  C  C  Q   Q  L  K   A  I  P
201 CCCAGTTGCT GATATGTCGT TCCTCCGGTC ACAGGCGGTG CAACCTAGGA GCTGCTTGGT AATGTGGGAG CAGTGTTGTC AGCAACTAAA AGCGATCCCC
     K  Q  S  R   C  E  A   I  H  N   V  V  H  A   I  I  L   Q  Q  Q   Q  Q  L  V   Q  G  I   S  T  Q   P  Q  Q  Q
301 AAGCAGTCAA GATGTGAGGC CATCCACAAC GTCGTGCACG CCATCATTTT GCAACAACAA CAACAATTGG TACAAGGTAT TTCCACCCAA CCTCAGCAGC
     · Q  Q  Q   G  Q  Q   Q  Q  G  Q   G  S  S   Q  P  Q   Q  Q  T  Q   L  D  Q   G  W  I   A  V  I  G   T  W  V
401 AACAACAACA AGGTCAACAA CAACAAGGTC AGGGTTCTAG CCAGCCACAA CAACAAACAC AACTGGATCA GGGTTGGATT GCGGTGATAG GGACATGGGT
     · I  Q  T   I  P  A  M   C  D  V   H  V  P   P  Y  C  Y   T  T  I   S  P  S   I  D  V  T   T  G  M   G  G  Y
501 GATTCAGACC ATTCCGGCAA TGTGCGACGT GCATGTTCCA CCATACTGCT ACACCACCAT ATCACCATCC ATTGACGTCA CTACCGGCAT GGGCGGCTAC
    TGA
```

Av-1b*

```
     M  K  I  F   L  A  L   A  F  L   A  T  M  A   T  I  T   I  V  T   S  D  V  T   L  P
  1 ATGAAGATCT TCCTCGCGCT TGCCTTTCTT GCCACTATGG CTACCATCAC CATAGTCACC TCCGATGTGA CACTTCCCTA GTTGCAGCCA CAAAAATCAT
101 TTTCACAGCA ACCACAACAA CAACCACCCT TTCCACAACC ACAACAACTA CCATTTCGAG AGGAGCGATC AACTCCACAA CAACACCCAT TTCCACCACA
201 ACAGTTACCA TTTGGGCAAC AACTATTTCC ACAACAACCA CCCTATCCCC CACCACAACA ACCACCCTAT CCCCAACCGC AACAACAACC ATTCCCACAA
301 CAACATGAAC AACCAGCTCA GGAACAACAA CCATTTGTAC AACAACAATT GAACCCATGT AGGGATGTCC TCGTACAACA GTGTAATCCA GTGGCAGCTT
401 TGTCATTTCT CCAGTCAGAG GTGGTGCAAC AGAGCAGATG CCAGGTGTTA AGGAAGCAAT GTTGT
```

Av-2b

```
      M  K  K  F   L  V  L   A  L  I   V  I  A  A   T  T  T   A  A  E   P  F  T  A   F  R  I   A  S  E   P  Q  H  P
  1  ATGAAGAAAT TCCTCGTCCT TGCCCTCATT GTCATCGCGG CGACCACCAC GGCCGCCGAA CCCTTTACTG CATTCCGTAT TGCTTCGGAG CCGCAACATC
      ·  S  S  P   E  Q  Q   P  T  L  Q   P  Q  E   H  P  V   P  H  Q  K   L  N  P   C  R  D   A  L  L  H   Q  C  S
101  CATCATCTCC AGAGCAGCAA CCAACTCTGC AGCCACAAGA ACATCCAGTT CCACATCAAA AGTTGAACCC TTGCAGGGAT GCCCTCCTAC ACCAGTGTAG
      · P  V  A   D  M  S  F   L  R  S   Q  V  V   Q  H  S  S   C  L  V   M  W  E   Q  C  C  Q   Q  L  K   A  I  P
201  CCCAGTAGCT GATATGTCGT TCCTCCGGTC ACAAGTGGTG CAACATAGCA GCTGCTTGGT AATGTGGGAG CAGTGTTGTC AGCAACTAAA AGCGATCCCC
      K  Q  S  R   C  E  A   I  H  N   V  V  H  A   I  I  L   Q  Q  Q   K  Q  L  V   Q  G  T   S  T  Q   P  Q  Q  Q
301  AAGCAGTCAA GATGTGAGGC CATCCACAAC GTCGTGCACG CCATTATTTT GCAACAGCAA AAACAATTGG TACAAGGTAC TTCAACCCAA CCTCAGCAGC
      ·  Q  Q  Q   G  Q  Q   E  H  G  Q   G  S  S   Q  P  Q   H  Q  Q  Q   Q  Q  Q   L  D  Q   G  W  I  V   A  I  G
401  AGCAACAACA AGGTCAACAA GAACATGGTC AGGGTTCTAG CCAGCCACAA CACCAACAAC AACAACAACA ACTGGATCAG GGTTGGATCG TGGCGATAGG
      · T  W  V   I  Q  T  V   P  A  M   C  D  V   H  V  P  P   Y  C  Y   T  T  I   S  S  S  S   D  V  T   T  G  M
501  GACATGGGTG ATTCAGACCG TTCCGGCAAT GTGCGACGTG CATGTTCCAC CATACTGCTA CACCACCATA TCATCATCGA GTGACGTCAC TACCGGAATG
      G  G  Y
601  GGCGGCTACTGA
```
