# Supplementary material for: Genome-Wide Identification, Characterization and Expression Pattern Analysis of the γ-Gliadin Gene Family in the Durum Wheat (Triticum durum Desf.) Cultivar Svevo
Source: Genes (Basel). 2021 Oct 29;12(11):1743. doi: 10.3390/genes12111743 (PMC8621147; doi:10.3390/genes12111743)
Supplement: Supplementary file 1 [file genes-12-01743-s001.zip › Supplemetary materials/Figure S2.pdf]

Figure S2. Alignment of the γ-gliadin **(a)** and δ-gliadin **(b)** gene sequences from the durum wheat cv. Svevo. The premature stop codons are boxed in red, the TGC codon that encodes the extra cysteine in the *Gli-γ1b* is boxed in blue, the region of Domain IV rich in glutamine codons is boxed in green.

(a)

```
         1                                                                                                100
Gli-γ1a  ATGAAGACCTTACTCATCCTGACAATCATTGCGGTGGCACTAACTACCACCACCGCCAATATACAGGTCGACCCTAGTGGCCAAGTACAATGGCCACAAC
Gli-γ2a* ATGAAGACCTTACTCATCCTCACAATCTTTGCAGCGGCTCTAACCATCGCCACCGCCAATATACAGGTCGACCCTAGCGGCCAAGTACAATGGCCACAAC
Gli-γ3a  ATGAAGACCTTATTCATCCTAACAATCCTTGCGATGGCAACAACTATCGCCACCGCGAATATGCAGGTCGACCCCAGCGGCCAAGTACAATGGCCACAAC
Gli-γ4a  ATGAAGACCTTATTCATCCTAACAATCCTTGCGATGGCAACAACTATCGCCACCGCGAATATGCAGGTCGACCCCAGCGGCCAAGTACAATGGCCACAAC
Gli-γ1b  ATGAAGACCTTACTCATCCTAACAATCCTTGCGATGGCAACAACCATCGCCACCGCCAATATGCAAGTCGACCCCAGCGGCCAAGTACAATGGCCACAAC
Gli-γ2b  ATGAAGACCTTACTCATCCTAACAATCCTTGCGATGGCAACAATAATCGCCACTGCCAATATGCAGGTCGACCCTAGCAGCCGAGTACAATGGCCACAAG
Gli-γ3b  ATGAAGACCTTACTCATCCTGACGATCCTTGCGATGGCAATAACCATCAGCACCGCCAATATGCAGGTCGACCCTAGTGGCCAAGTACAATGGCCACAAC
Gli-γ4b* ATGAAGACCTTACTCATCCTAACAATCCTTGCGATGGCAGTAACCATCGGCACCGCCAATATGCAGGTCGGCCCTAGCGGCCAAGTACAATGGCCACAAC
Gli-γ5b  ATGAAGACCTTACTCATCCTGACAATCCTTGCGATGGCAATAACCATCGCCACCGCCAATATGCAGGTCGACCCTAGCGGCCAAGTACAATGGCCGCAAC
         101                                                                                              200
Gli-γ1a  AACAACAACCATTCTCCCAGCCCCAACAACCATTCTCCCAACAACCACAACAAATTTTTCCCCAACCCCAACAAACATTCCCCCATCAACCACAACAAGC
Gli-γ2a* AACAAC---CATTCCCCTAGCCCCAAC---CATTCTCCCAGCAACCACAACAAG---------------------------------------------C
Gli-γ3a  AACAAC---CATTCCGCCAGCCCCAACAACCATTCTACCAGCAACCACAACAAA---------------------------------------------C
Gli-γ4a  AACAAC---CATTCCGCCAGCCCCAACAACCATTCTACCAGCAACCACAACAAA---------------------------------------------C
Gli-γ1b  AACAAC---CATTCCCCCAGCCCCAACAACCATTCTGCGAGCAACCACAACGAACTATTCCCCAACCCCATCAAACATTCCACCATCAACCACAACAAAC
Gli-γ2b  AACAAC---CATCCCCCCAGTCCCAACAACCATTCTCCCAGCAACCACAACAAATATTTCCCCAACCCCAACAAACATTGCCCCATCAACCACAACAAGC
Gli-γ3b  AACAAC---TAGTCCCCCAACCCCAACAGCCATTATCCCAGCAACCGCAACAAG---------------------------------------------C
Gli-γ4b* AACAAC---CAGTCCTGCTGCCTCAGCAACCATTCTCCCAGCAACCACAACAAA---------------------------------------------C
Gli-γ5b  AACAAC---CATTCCTGCAGCCTCACCAACCATTCTCCCAGCAACCACAACAAA---------------------------------------------T
         201                                                                                              300
Gli-γ1a  ATTTCCCCAACCCCAACAAACATTCCCCCATCAACCACAACAACAATTTCCCCAGCCCCAGCAACCACAACAACAATTTCCCCAGCCCCAACAACCACAA
Gli-γ2a* GTTTCCCCAACCCCAACAAACATTCCCCCTTCAACCACAGCAAGTATTTCCCCA---------ACCACAACAACAATTTCCCCAGCCCCAGCAACCACAA
Gli-γ3a  ATTTCCCCAACCCCAACAAACATTCCCCCATCAACCACAACAACAATTTCCCCAGCCCCAGCAACCACAACAACAATTTCCGCAGCCCCAGCAACCACAA
Gli-γ4a  ATTTCCCCAACCCCAACAAATATTCCCCCATCAACCACAACAACAATTTCCCCAGCCCCAACAACCACAACAACAATTTCCGCAGCCCCAGCAACCACAA
Gli-γ1b  ATTTCCCCAACCCGAACAAACATACCCCCATCAACCACAACAACAATTTCCCCAGACCCAACAACCACAACAACCATTTCCCCAGCCCCAACAAACATTC
Gli-γ2b  ATTTCCCCAACCTCAACAAACATTCCCCCATCGACCACAACAACAATTTCCCCAGCCCCAGCAACCACAACAACCATTTCCTCAGCCCCAACAA------
Gli-γ3b  ATTTCCCCAACCCCAACAAACATTTCCCCATCAACCACAACAACAAGTTCCCCAGCCTCAGCAACCACAACAACCATTTCTCCAGCCCCAACAAGCATTC
Gli-γ4b* ATTTCCCCAACCCCAACAAACATTCCCCCATCAACCACAACAATAATTTCCCCAGCCTCAACAACCACAACAACAATTTCTCCAGCCACAACAACCATTC
Gli-γ5b  ATTTCCCCAACCCCAACAAACATTCCCCCATCAACCACAACAACAATTTCCCCAGCCTCAGCAACCACAACAACAATTTCTCCAGCCCCGACAACCATTC
```

```
         301                                                                                              400
Gli-γ1a  CAACCATTTCCCCAGCAACCACAACAACAATTTCCCCGGC---------------CCCAACAACCACAACAACCATTTCCCCAGCCCCAACAACCCCAAC
Gli-γ2a* CAACCATTTCCCCAGC---------------------------------------------------------------------CCCAACAACCCCAAC
Gli-γ3a  CAACCATTTCCCCAAC---------------------------------------------------------------------CCCAACAAGCCCAAC
Gli-γ4a  CAACCATTTCCCCAGC---------------------------------------------------------------------CCCAACAAGCCCAAC
Gli-γ1b  CCCCAACAACCCCAACTACCATTTC------------------------------------------------------------CCCAACAACCCCAAC
Gli-γ2b  ---------CCCCAACTACCATTTC------------------------------------------------------------CCCAACAAACACAAC
Gli-γ3b  CCCCAACAACCACAACAACCATTCCCTCAGACTCAACAACCACAACAACCATTTCCCCAGCAACCACAACAACCATTCCCCCAGACTCAACAACCACAAC
Gli-γ4b* CCCCAACAACCACAACAACCATATCCCCAACAACCACAACAACCATTCCCCCAGACTCAACAACCCCAACAACTATTTCCCCAGTCCCAGCAACCACAAC
Gli-γ5b  CCCCAACAACCACAACAACCATATCCCCA-----------------------------------------------------------------------
         401                                                                                              500
Gli-γ1a  TACCATTTCC------GCAACAACCACAACAACCATTCCCCCAGCCTCAACAACCCCAACAACCATTTTCCCAGTTACAGCAACCACAACAACCTTTACC
Gli-γ2a* AACCATTCCCCCAGCCTCAACAACCCCAACAACCATTTCCCCAGTCACAGCAACCACAACAACCTTTTCCCCAG---------CCCCAACAACAATTCCC
Gli-γ3a  TACCATTTCC------TCAACAACCACAACAACCATTCCCCCAGCCTCAACAACCCCAACAACCATTTCCCCAGTCACAGCAACCACAACAACCTTTTCC
Gli-γ4a  TACCATTTCC------TCAACAACCACAACAACCATTCCCCCAGCCTCAACAACCCCAACAACCATTTCCCCAGTCACAGCAACCACAACAACCTTTTCC
Gli-γ1b  AACCATTCCCCCAGCCTCAGCAACCCCAACAACCATTTCCCCAGTCACAACAACCACAACAACCTTTTCCCCAG---------CCCCAACAACAATTTCC
Gli-γ2b  AACCATTCCCCCAGCCTCAACAACCCCAACAACCATTTCCCCAGTCACAGCAACCACAACAACCTTTTCCCCAG---------CCCCAACAACAATTTCC
Gli-γ3b  AACCATTTCCC------CAGCAACCACAACAACCATTCCCCCAGACTCAACAACCACAACAACCATTTCCCCAGTTCCAGCAACCACACCAACCTTTTCC
Gli-γ4b* AACCATATCCC------CAGCAACCACAACAACCATTCCCCCAGACTCAACAACCCCAACAACAATTTCCCCAATCCCAGCAACCACAACAACCATTTCC
Gli-γ5b  -------------------GCAACCACAACAACCGTTCCCCCAGACTCAACAACCCCAACAACCATTTCCCCAGTCCAAGCAACCACAACAACCTTTTCC
         501                                                                                              600
Gli-γ1a  CCAGCCCCAACAAT---------------------CGCAACAACCATTCTCCCAGCAACAACAATCATTGATTCAGCCATACCTACAACAACAGATGAAC
Gli-γ2a* GCAGCCCCAACAAC---------------------CGCAACAATCATTCCCCCAGCAGCAACAACTGTTGATTCAGCCATATCTACAACAACAGATGAAC
Gli-γ3a  CCAGCCCCAACAAC---------------------CGCAACAATCATTCCCCCAGCAACAACAACCGTTGATTCAGCCATATCTACAACAACAGATGAAC
Gli-γ4a  CCAGCCCCAACAAC---------------------CGCAACAATCATTCCCCCAGCAACAACAACCGTTGATTCAGCCATATCTACAACAACAGATGAAC
Gli-γ1b  GCAGCCCCAACAAC---------------------CACAACAATCATTCCCCCAACAACAACAACCGGCGATTCAGTCATTTCTACAACAACAGATGAAC
Gli-γ2b  GCAGCCCCAACAAC---------------------CACAACAATCATTCCCCCAACAACAACAATGGATGATTCAGTCATTTCTACAACAACAGATGAAC
Gli-γ3b  CCAGCCCCAACAACAATTCCCGCAGCCCCAACAACCGCAACAATCATTCCTCCAGCAACAACGACCATTCATTCAGCCATCTCTACAACAACGTTTGAAC
Gli-γ4b* CCAACCCCAACAAC---------------------TGCAACAATCATTCCCCCAGCAACAACCATCGTTCATTCAGCCATCTCTACAACAACTGCTGAAC
Gli-γ5b  CCAGCCCCAACAAC---------------------CGCAACAATCATTCCCCCAACAACAACCATCATTGATTCAACAATCTCTACAACAACAGTTGAAC
         601                                                                                              700
Gli-γ1a  CCCTGCAAGAATTACCTCTTGCAGCAATGCAACCCTGTGTCATTGGTGTCATCCCTCGTGTCAATGATCTTGCCACGAAGTGATTGCCAGGTGATGCGGC
Gli-γ2a* CCCTGCAAGAATTACCTCTTGCAGCAATGCAACCCCGTGTCATTAGTGTCGTCCCTCGTGTCAATGATCTTGCCACGAAGTGATTGCCAGGTGATGCAGC
Gli-γ3a  CCTTGCAAGAATTACCTCTTACAGCAATGCAACCCTGTGTCATTGGTGTCATCCCTCGTGTCAATGATCTTGCCACGAAGTGATTGCCAGGTGATGCAGC
Gli-γ4a  CCTTGCAAGAATTACCTCTTACAGCAATGCAACCCTGTGTCATTGGTGTCATCCCTCGTGTCAATGATCTTGCCACGAAGTGATTGCCAGGTGATGCAGC
Gli-γ1b  CCCTGCAAGAATTTCCTCTTGCAGCAATGCAACCATGTGTCATTGGTGTCATCTCTCGTGTCAATAATTTTGCCACGAAGTGATTGCCAGGTGATGCAGC
Gli-γ2b  CCCTGCAAGAATTTCCTCTTGCAGCAATGCAACCCTGTGTCATTGGTGTCATCTCTCGTGTCAATAATCTTGCCACGAAGTGATTGCCAGCTGATGCAGC
Gli-γ3b  CCATGCAAGAATATCCTCTTGCAACAATGCAAACCTGCGTCATTGGTGTCATCCCTCTGGTCGATAATCTGGCCACAAAGCGATTGCCAAGTGATGCAGC
Gli-γ4b* CCATGCAAGAATTTACTCTTGCAGCAATGTAGACCTGTGTCATTGGTCTCATCCCTCTGGTCAATGATCTGGCCACAAAGCGCTTGCCAAGTGATGAGGA
Gli-γ5b  CCATGCAAGAATTTCCTCTTGCAGCAATGCAAACCTGTGTCCTTGGTGTCATCCCTCTGGTCAATCATCTTGCCACCAAGCGATTGCCAGGTGATGCGGC
```

```
            701                                                                                              800
Gli-γ1a     AACAATGTTGCCAACAACTAGCACAGATTCCTCAGCAGCTCCAGTGCGCAGCCATCCATGGTGTCGTGCATTCCATCATCATGCAGCAAGAACAACAACA
Gli-γ2a*    AACAATGTTGCCAACAACTAGCACAGATTCCTCGTCAGCTCCAGTGCACAGCCATCCATAGCGTGGTGCATGCCATCATCATGCAGCAAGAACAAC---A
Gli-γ3a     AACAATGTTGCCAACAACTAGCACAGATTCCTCGCCAGCTCCAGTGTGCAGCCATCCATAGCGTCGTGCATTCCATCGTCATGCAGCAAGAACAACAACA
Gli-γ4a     AACAATGTTGCCAACAACTAGCACAGATTCCTCGCCAGCTCCAGTGTGCAGCCATCCATAGCGTCGTGCATTCCATCATCATGCAGCAAGAACAACAACA
Gli-γ1b     AACAATGTTGCCAACAACTAGCACAAATTCCTCAACAGCTCCAGTGCGCAGCCATCCACAGCGTCGCGCATTCCATCATCATGCAACAAGAACAACAACA
Gli-γ2b     AACAATGTTGCCAACAACTAGCACAAATTCCTCAACAGCTCCAGTGCGCAGCCATCCACAGCGTCGCGCATTCCATCGTCATGCAGCAAGAACAACAACG
Gli-γ3b     AACAATGCTGCCAAGAACTAGCACAGATTCCTCAGCAGCTCCAGTGCGCAGCCATCCATAGCGTCGTGCATTCCATCATCATGCAGCAGCAACAACAACA
Gli-γ4b*    AACAATGCTGCCAACAACTAGCACAGATTCCTCAGCAGCTCCAGTGTGCAGCCATCCATAGCGTCGTGCATTCCATCAGCATGTAGCAAGAACAACAACA
Gli-γ5b     AACAATGTTGTCAACAACTAGCACAAATTCCTCAGCAACTCCAGTGTGCAGCCATCCATAGCGTCGTGCATTCCATCATCATGCAGCAAGAACAACAAGA
            801                                                                                              900
Gli-γ1a     ACAACAA------------------------------------------GGCATACAGATCATGCGGCACTTTTCCAGC--------------------
Gli-γ2a*    A------------------------------------------------GGCATACAGATCCTGCGACCACTGTTTCAGC--------------------
Gli-γ3a     A------------------------------------------------GGCATACAGATCCTCCGGCCACTGTTTCAGC--------------------
Gli-γ4a     A------------------------------------------------GGCATACAGATCCTCCGGCCACTGTTTCAGC--------------------
Gli-γ1b     A------------------------------------------------GGCGTGCCGATCCTGCGGCCACTATTTCAGC--------------------
Gli-γ2b     A------------------------------------------------GGCGTGCAGATCCTGCGGCCACTATTTCAGC--------------------
Gli-γ3b     ACAACAACAACAACAACAACAACAA------------------------GGCATGCATATCCTGCTGACACTATCTCAACAACAACAGTTGGGTCAAGGT
Gli-γ4b*    ACAACAACAACAACAACAACAACAACAACAACAACAACAACAACAACAAGGCATGCGTATCCTGCTGCCACTATATCAGCAACAACAGGTGGGTCAAGGT
Gli-γ5b     ACAACTACAG---------------------------------------GGTGTGCAAATCCTGGTGCCACTGTCTCAACAGCAACAGGTGGGTCAAGGT
            901                                                                                             1000
Gli-γ1a     ----TCATCCAGGGTCAGGGCATCATCCAACCTCAACAACCAGCTCAATTGGAGGTGATCAGGTCATTGGTATTGGGAACTCTTCCAACCATGTGCAATG
Gli-γ2a*    ----TTGTCCAAGGACAGGGCATCATCCAACCTCAACAACCAGCTCAATATGAGGTGATCAGGTCATTGGTATTGAGAACCCTTCCAAACATGTGCAACG
Gli-γ3a     ----TCATCCAAGGTCAGGGCATCATCCAACCTCAACAACCAGCTCAATATGAGGTGATCAGGTCATTGGTATTGAGAACCCTTCCAAACATGTGCAACG
Gli-γ4a     ----TCGTCCAAGGTCAGGGCATCATCCAACCTCAACAACCAGCTCAATATGAGGTGATCAGGTCATTGGTATTGAGAACCCTTCCAAACATGTGCAACG
Gli-γ1b     ----TCGCCCAGGGTCTGGGTATCATCCAACCTCAACAACCAGCTCAATTGGAGGGGATCAGGTCATTGGTATTGAAAACTCTTCCAACCATGTGCAACG
Gli-γ2b     ----TCGCCCAGGGTCTGGGTATCATCCAACCTCAACAACCAGCTCAATTGGAGGGGATCAGGTCATTGGTATTGAAAACTCTTCCAACCATGTGCAATG
Gli-γ3b     ACTCTCGTCCAAGGCCAGGGCATCATCCAACCTCAACAACTAGCTCAATTGGAGGCGATCAGGTCATTGGTGTTGCAAACTCTTCCAACCATGTGCAACG
Gli-γ4b*    ACTCTCGTCCAGGGCCAGGGCATCATCCAACCCCAACAACCAGCTCAATTGGAGGCGATCAGGTCATTGGTGTTGCAAACTCTTCCAACCATGTGCAACG
Gli-γ5b     ATTCTCGTCCAGGGTCAAGGCATCATCCAACCTCAACAACCAGCTCAATTGGAGGTGATCAGGTCATTGGTGTTGCAAACTCTTCCAACCATGTGCAACG
            1001                                                                  1074
Gli-γ1a     TATTTGTTCCACCTGAGTGCTCCACCACCAAGGCACCATTTGCCAGCATAGTTGCCGACATTGGTGGCCAATGA
Gli-γ2a*    TGTATGTCCGACCTGACTGCTCCACCATCAACGCACCATTTGCCAGCATAGTTGCCGGCATTGGTGGCCAATGA
Gli-γ3a     TGTATGTCCGACCTGACTGCTCCACCATCAACGCACCATTTGCCAGCATAGTCGCCGGCATCAGTGGACAATGA
Gli-γ4a     TGTATGTCCGACCTGACTGCTCCACCATCAACGCACCATTTGCTAGCATAGTCGCCGGCATCAGTGGACAATGA
Gli-γ1b     TGTATGTGCCACCTGACTGCTCCACCATCAACGTACCATATGCCAACATAGACGCTGGCATTGGTGGCCAATGA
Gli-γ2b     TGTATGTCCCACCTGACTGCTCCACCATCAACGTGCCATATGCCAGCATAGACGCTGTCATTGGTGGCCAATGA
Gli-γ3b     TGTATGTCCCACCTGAGTGCTCCATCATCAGGGCACCATTTGCCAGCATAGTCGCGGGGATTGGTGGCCAATGA
Gli-γ4b*    TGTATGTCCCACCTGAGTGCTCTATCATCAAGGCACCATTTGCCAGCATAGTCACCGGAATTGGTGGCCAATGA
Gli-γ5b     TGTATGTCCCACCTTACTGCTCCACCATCAGGGCACCATTTGCTAGCATAGTCGCCAGCATTGGTGGCCAATGA
```

(b)

```
         1                                                                                                  100
Gli-δ1a* ATGAAGATCTTCTTGGTCTTTGCCCTCCTCGTTGTATCAACGATCATCACCACCGCGACCGTGCAGCTCGACCCTAGCATCCATGTACAAGAAAGGCCAC
Gli-δ1b* ATGAAGATCTTCTTGGTCTTTGCCCTCCTCGTTGTATCAACGACCATCACCACCGCGATCGTGCAGCTCGACCCTAGTGTCCATGTCCAAGAAAGGCCAC
Gli-δ2a* ATGAAGATCTTCTTGGTCTTTGCCCTCCTGGTTGCCACAACGATCAGCACCACCGGCACTGCGCAGCTCGACCCTAGCGTCCATGACTTAGAAAGGCCAC
         101                                                                                                200
Gli-δ1a* AACAATCATTTCCACAGCAGCAACCACTTAATCAACAACAACCATTCCCGCTGCAAGAGCCACAACAACCACTATTCCAGCAA-----------------
Gli-δ1b* AACAATCATTTCCGCAGCAGCAACCACTTACCCAGCAACAACCATTCCCGCTGCAAGAACCACAACAACCACTATTCCCGCAAAAAGAGCCACAACAACC
Gli-δ2a* ACCAATCATTTCCACAACAGCAGCCACTTCCCCCGCTACAACCATTCCCGCCGCAAGAGCCATAACAACCA---TTTCCGTTG-----------------
         201                                                                                                300
Gli-δ1a* -------------------------------CAACAACCGTATCCACAACAGTCACTTCCCCAACAACAACTTCCCCAGCAACATTTATTTCCGTAGCAA
Gli-δ1b* ATTTTTGCTGCAGCAACCACAACCCCAGGAACAACAACCATATCCACAACAGCCACTTCTCCAACAACAACTTCCCTAGCAACATCTATTCCCGCAGCAG
Gli-δ2a* -------------------------------------CAGCAACCACAACAC---CTGCAACAACAACCACTTCCCCAACAACATCTATTTCCGCAGCAA
         301                                                                                                400
Gli-δ1a* CCGCCGCAACAACAATTTCCACAGCAGATGCCACTTCCGTATCAACAACAAATATTCCCGCAACAACAACAACC------------CCCACAACAACAAC
Gli-δ1b* CCACCACAACAACAATTTCCGCAGCAGATGCCACTTCCATATGAACAACAAACATTCCCGCTACAACAGCAACGGCAACCACAATTCCCGCAACCACAAC
Gli-δ2a* CCGCCACAACAACAATTTCCACAGCA------------------ACAACAAACATTCCCGCAACCTCATCAACA---ACCACAATTCCCGAAACAACAAC
         401                                                                                                500
Gli-δ1a* CATTTTACCAATATCAACAACCATTAACACAACAACCATACCCGCAAGAGCAACCATTGCCACAACAACAACCTTCTGTGGAGGAAAACCAACAATTGAA
Gli-δ1b* CATTTCCCCAATATCAACAACCATTAACACAACAACCATACCCGCAAGAGCAACCATTGCCACAACAACAACCTTCTATGGAGGAAAAACAACAATTGAA
Gli-δ2a* CATTTTACCAATATCAACAACCATTTACACAACAACCATAACCGAAAGAGCAACCATTGGCACAGCAACAACCTTGTGTAGAGGAAAGACAACGACTGAA
         501                                                                                                600
Gli-δ1a* CTTGTGCAAGGAGTTCCTGCTGCAGCAGTGCAACCCGGAGGAGAAACTGTCATTACTGCAGTCAGTGATCCCGTTCCTCCGACCAAAGACCTCGCAACAG
Gli-δ1b* CGTGTGCAAGGAGTTCCTCCTGCAACAGTGCAACCCGGAGGAGAAACTGTCATTACTCCAGTCAGTGATCCCGTTCCTCCGACCAAAGACCTCGCAACAG
Gli-δ2a* CTTGTGCAAGGAATTCCTCTCGCAGCAGTGCAACCCGGAGGAGAAGGTGTCGTCACTCCATTCGGTGATCCCGTTCCTCTGACCAAAGACCTCGCAACAG
         601                                                                                                700
Gli-δ1a* AACAACTGCCAGTTGAAGCGGCAACAATGTTGTCGACAACTTGCACATATCAGCGAGCCGTCCCGATGCCCGACCATCCACAACATTGTGCACGCCATCA
Gli-δ1b* AATAGCTGCCAGTTGAAGCGTCTACAATGTTGTCAACAACTTGCACATATCAATGAACCGTCCCGATGCCCGGCCATCCACAACATTGTGCACGCCATCG
Gli-δ2a* AATAGCTGCCAGTTGAAGCGACAAGAATGTTGTCGCCAACTTGCACATATCCGTGAGCAGTCTTGATGCCCGGCCATCCAAAGCCCTGTGCATGCCATCA
         701                                                                                                800
Gli-δ1a* TTATGCAACAACAACAACAACAACAACAACAACAACAACATGTGGATAGAGGTTTTGTCCAGCCTCAACC-ACAACAGTTGGCCAGGGAATGCCCATGC
Gli-δ1b* TCGTTCAACAACAACA------------------------TGTGGATAGAGGTTTCGGCCAGCCTCAACC-ACAACAGTTGGCCAGGGAATGCCCATGC
Gli-δ2a* TTGTGCAACAACAACAACGACGACAACAACAACA------GGTTGATAGAGGTTTCGGCCAGCCTCAACCCACCACAGTTGGCCAGGGAATGCCCATGC
         801                                                                                                900
Gli-δ1a* AGCCTCAACATCAATTGGGCCAGGGCTTAAGCCTACCTCAACAACTAGCCCAGTTCAAGTTGGTTAGGTTACTTGTGATTCAGACCTTGCCTATGTTATG
Gli-δ1b* AGCCTCAATATCAATTGGGCCAGGGCTTTATCCTACCTCAACAACTAGCCCAGTTCAAGTTGGTTAGGTTACTTGTGATTCAGACCCTGCCTATGTTATG
Gli-δ2a* AACCTCAATAGCCACTGGGCCAGGGCTTTATCCTACCTCAGCAACTAGCCCAGTTTAAGTTGGTTAGGTCACTTGCGATTCAGACCCTGCCTATGTTATG
         901                                                                           979
Gli-δ1a* CAATGTGCATGTCCCATCTGATTGCTACACCATTAGTGCACCATTTGGTGGCATCACTGCCTACAACAGTGGACAATGA
Gli-δ1b* CAACGTGCATGTCCCATCTGATTGCTACACCACTAGTGCACCATCTGGTAGCATGACTGCCTTGAACGGTGGACAGTGA
Gli-δ2a* CAACGTGCATGTCCCGTCTGATTGCTACACCATTACTGCATATTTTGGTAGCATGACTGCCTGCAGCTGTGGACAATGA
```
