# Supplementary material for: Genome-Wide Identification, Characterization and Expression Pattern Analysis of the γ-Gliadin Gene Family in the Durum Wheat (Triticum durum Desf.) Cultivar Svevo
Source: Genes (Basel). 2021 Oct 29;12(11):1743. doi: 10.3390/genes12111743 (PMC8621147; doi:10.3390/genes12111743)
Supplement: Supplementary file 1 [file genes-12-01743-s001.zip › Supplemetary materials/Figure S3.pdf]

Figure S3. Alignment of the amino acid sequences deduced from the pseudogenes encoding γ-gliadins **(a)** and δ-gliadins **(b)**. Roman numerals indicate conserved domains as assigned by Anderson and coworkers [31]. The eight conserved cysteine residues are indicated with an asterisk, the first premature stop codon is indicated with a red circle. The heptapeptide motif PQQPFPQ is highlighted in bold and underlined. The alignments were performed using Vector NTI Suite software (version 9.0; Thermo Fisher Scientific, Waltham, MA, USA).

(a)

```
                            1           S           I                         II                            100
A. longissima (FJ006648)    MKTLLILTILAMAITIATANMQVDPSGQVQWPQQQPFPQPHQPFSQQPQQIFPQPQQTFPHQPQQQFPQPQQPQQQFLQPQQP---FP--QQPQQPYPQQ
   T. aestivum (γ-B5*)      MKTLLILTILVMAVTIGTANMQVDPSGQVQWPQQQPVLLPQQPFSQQPQQTFPQPQQTFPHQPQQQFPQPQQPQQQFLQPQQP---FP--QQPQQPYPQQ
   T. durum (Gli-γ4b*)      MKTLLILTILAMAVTIGTANMQVGPSGQVQWPQQQPVLLPQQPFSQQPQQTFPQPQQTFPHQPQQ●FPQPQQPQQQFLQPQQP---FP--QQPQQPYPQQ
   T. aestivum (γ-A2*)      MKTLLILTIFAAALTIATANIQVDPSGQVQWPQQQPFSQ-PQPFSQQPQQAFPQPQQTSPLQPQ●VFPQPQQPQQQFPQPQQPQQPFP-PQQPQLPFPQQ
   T. durum (Gli-γ2a*)      MKTLLILTIFAAALTIATANIQVDPSGQVQWPQQQPFP●-PQPFSQQPQQAFPQPQQTFPLQPQQVFPQPQQ---QFPQPQQPQQPFPQPQQP-------
T. monococcum (FJ006629)    MKTLLILTIFAAALTIATANIQVAPSGQVQWPQQQPFPQ-PQPFSQQPQQAFPQPQQTFPLQPHQVFPQPQQPQQQFPQPQQPQQPFPQPQQPQLPF---

                            101                               II                                 *      *       200
A. longissima (FJ006648)    PQQPFPQIQQPQQPFPQSKQPQQPFPQ-----------PQQQFPQPQQPQ----------QSFPQQQPSLIQQSLQQQLNPCKNFLLQQCKPVSLVSSLW
   T. aestivum (γ-B5*)      PQQPFPQTQQPQQLFPQSQQPQQPYPQQPQQPFPQTQQPQQQFPQSQQPQ●PFPQPQQPQQSFPQQQPSFIQPSLQQQLNPCKNLLLQQCRPVSLVSSLW
   T. durum (Gli-γ4b*)      PQQPFPQTQQPQQLFPQSQQPQQPYPQQPQQPFPQTQQPQQQFPQSQQPQQPFPQPQQLQQSFPQQQPSFIQPSLQQLLNPCKNLLLQQCRPVSLVSSLW
   T. aestivum (γ-A2*)      PQQPFPQPHQPQQPFPQSQQPQQPFPQ-----------PQQQFPQPQQKQ----------QSFPQQQQPLIQPYLQQQMNPCKNYLLQQCNPVSLVSSLV
   T. durum (Gli-γ2a*)      -QQPFPQPQQPQQPFPQSQQPQQPFPQ-----------PQQQFPQPQQPQ----------QSFPQQQQLLIQPYLQQQMNPCKNYLLQQCNPVSLVSSLV
T. monococcum (FJ006629)    PQQPFPQPQQPQQPFPQSQQPQQPFLQ-----------PQQQFRQPQQPQ----------QSFPQQQQPLIQPYLQQQMNPCKNYLLQQCNPVSLVSSLV

                            201      *     ** III     *                          IV                            300
A. longissima (FJ006648)    SIILPRSDCQVMRQQCCQQLAQIPQQLQCAAIHSVVHSIIMQQEQQEQLQ-------------------------SGQILVPLSQQQQVGQGILVQGQGI
   T. aestivum (γ-B5*)      SMIWPQSACQVMRQQCCQQLAQIPQQLQCAAIHSVVHSISM-QEQQQQQQQQQQQQQQQQQQQQQQQQQQQQQQQGMRILLPLYQQQQVGQGTLVQGQGI
   T. durum (Gli-γ4b*)      SMIWPQSACQVMRKQCCQQLAQIPQQLQCAAIHSVVHSISM-QEQQQQQQQQQQQQQQQQQ------------GMRILLPLYQQQQVGQGTLVQGQGI
   T. aestivum (γ-A2*)      SMILPRSDCQVMQQQCCQQLAQIPRQLQCTAIHSVVHAIIMQQEQQ-----------------------------GIQILRPLFQ--------LVQGQGI
   T. durum (Gli-γ2a*)      SMILPRSDCQVMQQQCCQQLAQIPRQLQCTAIHSVVHAIIMQQEQQ-----------------------------GIQILRPLFQ--------LVQGQGI
T. monococcum (FJ006629)    SMILPRSDCQVMQQQCCQQLAQIPRQLQCTAIHSVVHAIIMQQEQQ-----------------------------GIQILRPLFQ--------LVQGQGI

                            301                      * V    *          349
A. longissima (FJ006648)    IQPQQPAQLEVIRSLVLQTLPTMCNMYVPPYCSTIRAPFASIVAGIGGQ
   T. aestivum (γ-B5*)      IQPQQPAQLEAIRSLVLQTLPTMCNVYVPPECSIIKAPFASIVTGIGGQ
   T. durum (Gli-γ4b*)      IQPQQPAQLEAIRSLVLQTLPTMCNVYVPPECSIIKAPFASIVTGIGGQ
   T. aestivum (γ-A2*)      IQPQQPAQYEVIRSLVLRTLPNMCNMYVRPNCSTINAPFASIVAGIGGQ
   T. durum (Gli-γ2a*)      IQPQQPAQYEVIRSLVLRTLPNMCNVYVRPDCSTINAPFASIVAGIGGQ
T. monococcum (FJ006629)    IQPQQPAQYEVIRSLVLRTLPNMCNVYVRPDCSTINAPFASIVAGIGGQ
```

(b)

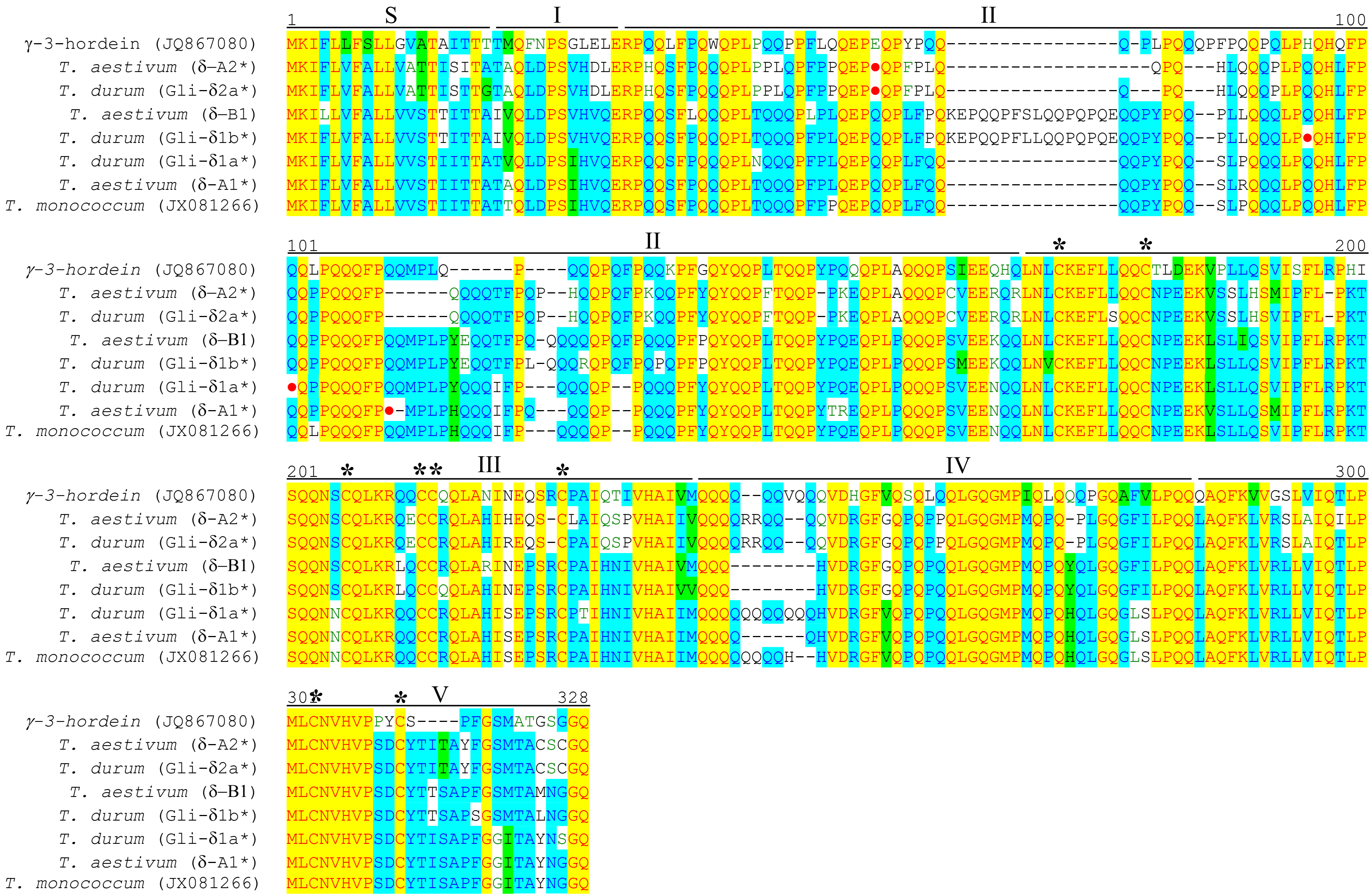

1 S I II 100
γ-3-hordein (JQ867080) MKIFLLFSLLGVATAITTTMQFNPSGLELERPQQLFPQWQPLPQQPPFLQQEPEQPYPQQ----------------Q-PLPQQQPFPQQPQLPHQHQFP
T. aestivum (δ-A2*) MKIFLVFALLVATTISITATAQLDPSVHDLERPHQSFPQQQPLPPLQPFPPQEP●QPFPLQ--------------------QPQ---HLQQQPLPQQHLFP
T. durum (Gli-δ2a*) MKIFLVFALLVATTISTTGTAQLDPSVHDLERPHQSFPQQQPLPPLQPFPPQEP●QPFPLQ----------------Q---PQ---HLQQQPLPQQHLFP
T. aestivum (δ-B1) MKILLVFALLVVSTTITTAIVQLDPSVHVQERPQQSFLQQQPLTQQQPLPLQEPQQPLFPQKEPQQPFSLQQPQPQEQQPYPQQ--PLLQQQLPQQHLFP
T. durum (Gli-δ1b*) MKIFLVFALLVVSTTITTAIVQLDPSVHVQERPQQSFPQQQPLTQQQPFPLQEPQQPLFPQKEPQQPFLLQQPQPQEQQPYPQQ--PLLQQQLP●QHLFP
T. durum (Gli-δ1a*) MKIFLVFALLVVSTIITTATVQLDPSIHVQERPQQSFPQQQPLNQQQPFPLQEPQQPLFQQ----------------QQPYPQQ--SLPQQQLPQQHLFP
T. aestivum (δ-A1*) MKIFLVFALLVVSTIITTATAQLDPSIHVQERPQQSFPQQQPLTQQQPFPLQEPQQPLFQQ----------------QQPYPQQ--SLRQQQLPQQHLFP
T. monococcum (JX081266) MKIFLVFALLVVSTIITTATTQLDPSIHVQERPQQSFPQQQPLTQQQPFPPQEPQQPLFQQ----------------QQPYPQQ--SLPQQQLPQQHLFP
101 II 200
γ-3-hordein (JQ867080) QQLPQQQFPQQMPLQ------P----QQQPQFPQQKPFGQYQQPLTQQPYPQQQPLAQQQPSIEEQHQLNLCKEFLLQQCTLDEKVPLLQSVISFLRPHI
T. aestivum (δ-A2*) QQPPQQQFP------QQQQTFPQP--HQQPQFPKQQPFYQYQQPFTQQP-PKEQPLAQQQPCVEERQRLNLCKEFLLQQCNPEEKVSSLHSMIPFL-PKT
T. durum (Gli-δ2a*) QQPPQQQFP------QQQQTFPQP--HQQPQFPKQQPFYQYQQPFTQQP-PKEQPLAQQQPCVEERQRLNLCKEFLSQQCNPEEKVSSLHSVIPFL-PKT
T. aestivum (δ-B1) QQPPQQQFPQQMPLPYEQQTFPQ-QQQQQPQFPQQQPFPQYQQPLTQQPYPQEQPLPQQQPSVEEKQQLNLCKEFLLQQCNPEEKLSLIQSVIPFLRPKT
T. durum (Gli-δ1b*) QQPPQQQFPQQMPLPYEQQTFPL-QQQRQPQFPQPQPFPQYQQPLTQQPYPQEQPLPQQQPSMEEKQQLNVCKEFLLQQCNPEEKLSLLQSVIPFLRPKT
T. durum (Gli-δ1a*) ●QPPQQQFPQQMPLPYQQQIFP---QQQQP--PQQQPFYQYQQPLTQQPYPQEQPLPQQQPSVEENQQLNLCKEFLLQQCNPEEKLSLLQSVIPFLRPKT
T. aestivum (δ-A1*) QQPPQQQFP●-MPLPHQQQIFPQ---QQQP--PQQQPFYQYQQPLTQQPYTREQPLPQQQPSVEENQQLNLCKEFLLQQCNPEEKVSLLQSMIPFLRPKT
T. monococcum (JX081266) QQLPQQQFPQQMPLPHQQQIFP---QQQQP--PQQQPFYQYQQPLTQQPYPQEQPLPQQQPSVEENQQLNLCKEFLLQQCNPEEKLSLLQSVIPFLRPKT
201 III IV 300
γ-3-hordein (JQ867080) SQQNSCQLKRQQCCQQLANINEQSRCPAIQTIVHAIVMQQQQ--QQVQQQVDHGFVQSQLQQLGQGMPIQLQQQPGQAFVLPQQQAQFKVVGSLVIQTLP
T. aestivum (δ-A2*) SQQNSCQLKRQECCRQLAHIHEQS-CLAIQSPVHAIIVQQQQRRQQ--QQVDRGFGQPQPPQLGQGMPMQPQ-PLGQGFILPQQLAQFKLVRSLAIQILP
T. durum (Gli-δ2a*) SQQNSCQLKRQECCRQLAHIREQS-CPAIQSPVHAIIVQQQQRRQQ--QQVDRGFGQPQPPQLGQGMPMQPQ-PLGQGFILPQQLAQFKLVRSLAIQTLP
T. aestivum (δ-B1) SQQNSCQLKRLQCCRQLARINEPSRCPAIHNIVHAIVMQQQ---------HVDRGFGQPQPQQLGQGMPMQPQYQLGQGFILPQQLAQFKLVRLLVIQTLP
T. durum (Gli-δ1b*) SQQNSCQLKRLQCCQQLAHINEPSRCPAIHNIVHAIVVQQQ---------HVDRGFGQPQPQQLGQGMPMQPQYQLGQGFILPQQLAQFKLVRLLVIQTLP
T. durum (Gli-δ1a*) SQQNNCQLKRQQCCRQLAHISEPSRCPTIHNIVHAIIMQQQQQQQQQQQHVDRGFVQPQPQQLGQGMPMQPQHQLGQGLSLPQQLAQFKLVRLLVIQTLP
T. aestivum (δ-A1*) SQQNNCQLKRQQCCRQLAHISEPSRCPAIHNIVHAIIMQQQQ------QHVDRGFVQPQPQQLGQGMPMQPQHQLGQGLSLPQQLAQFKLVRLLVIQTLP
T. monococcum (JX081266) SQQNNCQLKRQQCCRQLAHISEPSRCPAIHNIVHAIIMQQQQQQQQH--HVDRGFVQPQPQQLGQGMPMQPQHQLGQGLSLPQQLAQFKLVRLLVIQTLP
301 V 328
γ-3-hordein (JQ867080) MLCNVHVPPYCS----PFGSMATGSGGQ
T. aestivum (δ-A2*) MLCNVHVPSDCYTITAYFGSMTACSCGQ
T. durum (Gli-δ2a*) MLCNVHVPSDCYTITAYFGSMTACSCGQ
T. aestivum (δ-B1) MLCNVHVPSDCYTTSAPFGSMTAMNGGQ
T. durum (Gli-δ1b*) MLCNVHVPSDCYTTSAPSGSMTALNGGQ
T. durum (Gli-δ1a*) MLCNVHVPSDCYTISAPFGGITAYNSGQ
T. aestivum (δ-A1*) MLCNVHVPSDCYTISAPFGGITAYNGGQ
T. monococcum (JX081266) MLCNVHVPSDCYTISAPFGGITAYNGGQ
