# Supplementary material for: Genome-Wide Identification, Characterization and Expression Pattern Analysis of the γ-Gliadin Gene Family in the Durum Wheat (Triticum durum Desf.) Cultivar Svevo
Source: Genes (Basel). 2021 Oct 29;12(11):1743. doi: 10.3390/genes12111743 (PMC8621147; doi:10.3390/genes12111743)
Supplement: Supplementary file 1 [file genes-12-01743-s001.zip › Supplemetary materials/Figure S4.pdf]

Figure S4. Alignment of the amino acid sequences deduced from the γ-gliadin genes of the cv. Svevo and other durum wheat cvs. The γ-gliadin gene sequences from the different durum wheat cvs. were retrieved from the GenBank database. The Roman numerals indicate conserved domains as assigned by Anderson and coworkers [31]. The first premature stop codon is indicated with a red circle, the eight conserved cysteine residues are indicated with black asterisks, the extra cysteine residue is boxed in red. The heptapeptide motif PQQPFPQ is highlighted in bold and underlined. The alignments were performed using Vector NTI Suite software (version 9.0; Thermo Fisher Scientific, Waltham, MA, USA).

```
                          S                I                          II
                       1                                                                                                  100
AJ389699_cv._Astodur   ----------------GTANMQVGPSGQVQWPQQ-QPVLLPQQPFSQQPQQTFPQPQQTFPHQPQQ•FPQPQQPQQQFLQPQQPFPQQPQQ---------
AJ389700_cv._Duilio    ----------------GTANMQVGPSGQVQWPQQ-QPVLLPQQPFSQQPQQTFPQPQQTFPHQPQQ•FPQPQQPQQQFLQPQQPFPQQPQQ---------
AJ389701_cv._DT433     ----------------GTANMQVGPSGQVQWPQQ-QPVLLPQQPFSQQPQQTFPQPQQTFPHQPQQ•FPQPQQPQQQFLQPQQPFPQQPQQ---------
Svevo (Gli-γ4b*)       MKTLLILTILAMAVTIGTANMQVGPSGQVQWPQQ-QPVLLPQQPFSQQPQQTFPQPQQTFPHQPQQ•FPQPQQPQQQFLQPQQPFPQQPQQ---------
AJ389703_cv._Rugby     ----------------GTANMQVDPSGQVQWPQQ-QPVLLPQQPFSQQPQQTFPQPQQTFPHQPQQQFPQPQQPQQQFLQPQQPFPQQPQQ---------
AJ389704_cv._Wascana   ------------------ANMQVDPSGQVQWPQQ-QPVLLPQQPFSQQPQQTFPQPQQTFPHQPQQQFPQPQQPQQQFLQPQQPFPQQPQQ---------
Svevo (Gli-γ3b)        MKTLLILTILAMAITISTANMQVDPSGQVQWPQQ-QLVPQPQQPLSQQPQQAFPQPQQTFPHQPQQQVPQPQQPQQPFLQPQQAFPQQPQQPFPQTQQPQ
AY338391_cv._TD92      ------------------MNIQVDPSSQVQWPQQ-QPFLQPHQPFSQQPQQIFPQPQQTFPHQPQQQFPQPQ-------QPQRQFLQ-------------
AY338394_cv._TD1A23    ------------------MNIQVDPSSQVQWPQQ-QPFLQPHQPFSQQPQQIFP---------------------------------------------
AY338393_cv._TD1A23    ------------------MNIQVDPSSQVQWPQQ-QLVPQPQQPLSQQPQQAFPQPQQTFPHQPQQQVPQPQ-------QPQQPFLQ-------------
FJ006578_cv._AS2255    MKTLLILTILAMAITIATANMQVDPSGQVQWPQQ-QPFLQPYQPFSQQPQQIFPQPQQTFPHQPQQQFPQPQ-------QPQQQFLQ-------------
FJ006585_cv._AS2255    MKTLLILTILAMAITIATANMQVDPSGQVQWPQQ-QPFLQPYQPFSQQPQQIFPQPQQTFPHQPQQQFPQPQ-------QPQQQFLQ-------------
FJ006588_cv._AS2255    MKTLLILTILAMAITIATANMQVDPSGQVQWPQQ-QPFLQPYQPFSQQPQQIFPQPQQTFPHQPQQQFPQPQ-------QPQQQFLQ-------------
Svevo (Gli-γ5b)        MKTLLILTILAMAITIATANMQVDPSGQVQWPQQ-QPFLQPHQPFSQQPQQIFPQPQQTFPHQPQQQFPQPQ-------QPQQQFLQ-------------
AY338392_cv._TD92      ------------------MNIQVDPSSQVQWPQQ-QPFLQPHQPFSQQPQQIFP---------------------------------------------
Svevo (Gli-γ1a)        MKTLLILTIIAVALTTTANIQVDPSGQVQWPQQQQPFSQPQQPFSQQPQQIFPQPQQTFPHQPQQAFPQPQQTFP--HQPQQQFPQ-------------
FJ006577_cv._AS2255    MKTLLILTILAMATTIATANMQVDPSGQVQWPQQ-QPFPQPQQPFCQQPQRTIPQPHQTFHHQPQQTFPQPQQTYP--HQPQQQFPQ-------------
Svevo (Gli-γ1b)        MKTLLILTILAMATTIATANMQVDPSGQVQWPQQ-QPFPQPQQPFCEQPQRTIPQPHQTFHHQPQQTFPQPEQTYP--HQPQQQFPQ-------------
X77963_cv._pTD9        --------------------MQVDPSGQVQWPQQ-QPFPQPQQPFCEQPQRTIPQPHQTFHHQPQQTFPQPEQTYP--HQPQQQFPQ-------------
X53412_cv._Langdon     MKTLLILTILAMATTIATANMQVDPSGQVQWPQQ-QPFPQPQQPFCEQPQRTIPQPHQTFHHQPQQTFPQPEQTYP--HQPQQQFPQ-------------
Svevo (Gli-γ2b)        MKTLLILTILAMATIIATANMQVDPSSRVQWPQE-QPSPQSQQPFSQQPQQIFPQPQQTLPHQPQQAFPQPQQTFP--HRPQQQFPQ-------------
FJ006580_cv._AS2255    MKTLFILTILAMATTIATANMQVDPSGQVQWPQQ-QPFR---------------QPQQPFYQQPQQTFPQPQ-------Q---TFPR-------------
Svevo (Gli-γ2a*)       MKTLLILTIFAAALTIATANIQVDPSGQVQWPQQ-QPFP•PQ-PFSQQPQQAFPQPQQTFPLQPQQVFPQPQ-------Q---QFPQ-------------
FJ006586_cv._AS2255    MKTLLILTILAMAVTIGTANMQVDPSGQVQWPQQ-QPVLLPQQPFSQQPQQTFPQPQQTFPHQPQQQFPQPQ-------QPQQQFLQ-------------
FJ006581_cv._AS2255    MKTLFILTILAMATTIATANMQVDPSGQVQWPQQ-QPFRQPQQPFYQQPQQTFPQPQQTFPHQPQQQFPQPQ-------QPQQQFPQ-------------
FJ006582_cv._AS2255    MKTLFILTILAMATTIATANMQVDPSGQVQWPQQ-QPFRQPQQPFYQQPQQTFPQPQQTFPHQPQQQFPQPQ-------QPQQQFPQ-------------
FJ006584_cv._AS2255    MKTLFILTILAMATTIATANMQVDPSGQVQWPQQ-QPFRQPQQPFYQQPQQTFPQPQQTFPHQPQQQFPQPQ-------QPQQQFPQ-------------
Svevo (Gli-γ3a)        MKTLFILTILAMATTIATANMQVDPSGQVQWPQQ-QPFRQPQQPFYQQPQQTFPQPQQTFPHQPQQQFPQPQ-------QPQQQFPQ-------------
FJ006583_cv._AS2255    MKTLFILTILAMATTIATANMQVDPSGQVQWPQQ-QPFRQPQQPFHQQPQQTFPQPQQIFPHQPQQQFPQPQ-------QPQQQFPQ-------------
FJ006587_cv._AS2255    MKTLFILTILAMATTIATANMQVDPSGQVQWPQQ-QPFRQPQQPFYQQPQQTFPQPQQIFPHQPQQQFPQPQ-------QPQQQFPQ-------------
FJ006579_cv._AS2255    MKTLFILTILAMATTIATANMQVDPSGQVQWPQQ-QPFRQPQQPFYQQPQQTFPQPQQIFPHQPQQQFPQPQ-------QPQQQFPQ-------------
Svevo (Gli-γ4a)        MKTLFILTILAMATTIATANMQVDPSGQVQWPQQ-QPFRQPQQPFYQQPQQTFPQPQQIFPHQPQQQFPQPQ-------QPQQQFPQ-------------
```

II

```
                          101                                                                                              200
AJ389699_cv._Astodur      -PYPQQPQQPFPQTQQPQQLFPQSQQPQQPYPQQPQQPFPQTQQPQQQFPQSQQP--------QQPFPQPQQ--------------------LQQSFPQQ
AJ389700_cv._Duilio       -PYPQQPQQPFPQTQQPQQLFPQSQQPQQPYPQQPQQPFPQTQQPQQQFPQSQQP--------QQPFPQPQQ--------------------LQQSFPQQ
AJ389701_cv._DT433        -PYPQQPQQPFPQTQQPQQLFPQSQQPQQPYPQQPQQPFPQTQQPQQQFPQSQQP--------QQPFPQPQQ--------------------LQQSFPQQ
Svevo (Gli-γ4b*)          -PYPQQPQQPFPQTQQPQQLFPQSQQPQQPYPQQPQQPFPQTQQPQQQFPQSQQP--------QQPFPQPQQ--------------------LQQSFPQQ
AJ389703_cv._Rugby        -PYPHQPQQPFPQTQQPQQLFPQSQQPQQPYPQQPQQPFPQTQQPQQQFPQSQQP--------QQPFPQPQQ--------------------PQQSFLQQ
AJ389704_cv._Wascana      -PYPHQPQQPFPQTQQPQQLFPQSQQPQQPYPQQPQQPFPQTQQPQQQFPQSQQP--------QQPFPQPQQ--------------------PQQSFLQQ
Svevo (Gli-γ3b)           QPFPQQPQQPFPQTQ----------QPQQPFPQQPQQPFPQTQQPQQPFPQFQQP--------HQPFPQPQQQ-------------FPQPQQPQQSFLQQ
AY338391_cv._TD92         ------PRQPFPQ------------QPQQPYPQQPQQPFPQTQQPQQPFPQSKQP--------QQPFPQPQQ--------------------PQQSFPQQ
AY338394_cv._TD1A23       -------------------------QPQQPYPQQPQQPFPQTQQPQQPFPQSKQP--------QQPFPQPQQ--------------------PQQSFPQQ
AY338393_cv._TD1A23       ------PRQPFPQ------------QPQQPYPQQPQQPFPQTQQPQQPFPQSKQP--------QQPFPQPQQ--------------------PQQSFPQQ
FJ006578_cv._AS2255       ------PRQPFPQ------------QPQQPYPQQPQQPFPQTQQPQQPFPQSKQP--------QQPFPQPQQ--------------------PQQSFPRQ
FJ006585_cv._AS2255       ------PRQPFPQ------------QPQQPYPQQPQQPFPQTQQPQQSFPQSKQP--------QQPFPQPQQ--------------------PQQSFPQQ
FJ006588_cv._AS2255       ------PRQPFPQ------------QPQQSYPQQPQQPFPQTQQPQQPFPQSKQP--------QQPFPQPQQ--------------------PQQSFPQQ
Svevo (Gli-γ5b)           ------PRQPFPQ------------QPQQPYPQQPQQPFPQTQQPQQPFPQSKQP--------QQPFPQPQQ--------------------PQQSFPQQ
AY338392_cv._TD92         -------------------------QPQQTFPHQPQQPFPQSQQPQQPFP---QP--------QQQFPQPQQ--------------------PQQSFPQQ
Svevo (Gli-γ1a)           ---PQQPQQQFPQPQ----------QPQQPFPQQPQQQFPRPQQPQQPFPQPQQPQLPFPQQPQQPFPQPQQPQQPFSQLQQPQQPLPQPQQSQQPFSQQ
FJ006577_cv._AS2255       ---TQQPQQPFPQPQQTF-----PQQPQLPFPQQPQQPFPQPQQPQQPFPQSQQP--------QQPFPQPQQQ-------------FPQPQQPQQSFPQQ
Svevo (Gli-γ1b)           ---TQQPQQPFPQPQQTF-----PQQPQLPFPQQPQQPFPQPQQPQQPFPQSQQP--------QQPFPQPQQQ-------------FPQPQQPQQSFPQQ
X77963_cv._pTD9           ---TQQPQQPFPQPQQTF-----PQQPQLPFPQQPQQPFPQPQQPQQPFPQSQQP--------QQPFPQPQQQ-------------FPQPQQPQQSFPQQ
X53412_cv._Langdon        ---TQQPQQPFPQPQQTF-----PQQPQLPFPQQPQQPFPQPQQPQQPFPQSQQP--------QQPFPQPQQQ-------------FPQPQQPQQSFPQQ
Svevo (Gli-γ2b)           ---PQQPQQPFPQPQ----------QPQLPFPQQTQQPFPQPQQPQQPFPQSQQP--------QQPFPQPQQQ-------------FPQPQQPQQSFPQQ
FJ006580_cv._AS2255       -----QPQLPFPQ--------------------QPQQPFPQPQQPQQPFPQSQQP--------QQPFPQPQQQ-------------FPQPQQPQQSFPQQ
Svevo (Gli-γ2a*)          ---PQQPQQPFPQPQ------------------QPQQPFPQPQQPQQPFPQSQQP--------QQPFPQPQQQ-------------FPQPQQPQQSFPQQ
FJ006586_cv._AS2255       ------PQQPFPQ------------QPQQPYPQQPQQPFPQTQQPQQPFPQSQQP--------QQPFPQPQQ--------------------PQQSFPQQ
FJ006581_cv._AS2255       ---PQQPQQPFPQPQ----------QAQLPFPQQPQQPFPQPQQPQQPFPQSQQP--------QQPFPQPQQ--------------------PQQSFPQQ
FJ006582_cv._AS2255       ---PQQPQQPFPQPQ----------QAQLPFPQQPQQPFPQPQQPQQPFPQSQQP--------QQPFPQPQQ--------------------PQQSFPQQ
FJ006584_cv._AS2255       ---PQQPQQPFPQPQ----------QAQLPFPQQPQQPFPQPQQPQQPFPQSQQP--------QQPFPQPQQ--------------------PQQSFPQQ
Svevo (Gli-γ3a)           ---PQQPQQPFPQPQ----------QAQLPFPQQPQQPFPQPQQPQQPFPQSQQP--------QQPFPQPQQ--------------------PQQSFPQQ
FJ006583_cv._AS2255       ---PQQPQQPFPQPQ----------QAQLPFPQQPQQPFPQPQQPQQPFPQSQQP--------QQPFPQPQQ--------------------PQQSFPQQ
FJ006587_cv._AS2255       ---PQQPQQPFPQPQ----------QAQLPFPQQPQQPFPQPQQPQQPFPQSQQP--------QQPFPQPQQ--------------------PQQSFPQQ
FJ006579_cv._AS2255       ---PQQPQQPFPQPQ----------QAQLPFPQQPQQPFPQPQQPQQPSPQSQQP--------QQPFPQPQQ--------------------PQQSFPQQ
Svevo (Gli-γ4a)           ---PQQPQQPFPQPQ----------QAQLPFPQQPQQPFPQPQQPQQPFPQSQQP--------QQPFPQPQQ--------------------PQQSFPQQ
```

```
                                                                 III                                   IV
                          201           *         *            *      **            *                     300
AJ389699_cv._Astodur      QPSFIQPSLQQLLNPCKNLLLQQCRPVSLVSSLWSMIWPQSACQVMRKQCCQQLAQIPQQLQCAAIHSVVHSISM-QEQQQQQQQQQQQQQQQQQQQGMR
AJ389700_cv._Duilio       QPSFIQPSLQQLLNPCKNLLLQQCRPVSLVSSLWSMIWPQSACQVMRKQCCQQLAQIPQQLQCAAIHSVVHSISM-QEQQQQQQQQQQQQQQQQQQQGMR
AJ389701_cv._DT433        QPSFIQPSLQQLLNPCKNLLLQQCRPVSLVSSLWSMIWPQSACQVMRKQCCQQLAQIPQQLQCAAIHSVVHSISM-QEQQQQQQQQQQQQQQQQQQ----
Svevo (Gli-γ4b*)          QPSFIQPSLQQLLNPCKNLLLQQCRPVSLVSSLWSMIWPQSACQVMRKQCCQQLAQIPQQLQCAAIHSVVHSISM-QEQQQQQQQQQQQQQQQQQQQGMR
AJ389703_cv._Rugby        QPSFTQPSLQQQLNPCKNLLLQQCRPVSLVSSLWSMIWPQSDCQVMRQQCCQQLAQIPQQLQCAAIHSVVHSISM-QEQQQQQQQQQ----------GMR
AJ389704_cv._Wascana      QPSFTQPSLQQQLNPCKNLLLQQCRPVSLVSSLWSMIWPQSDCQVMRQQCCQQLAQIPQQLQCAAIHSVVHSISM-QEQQQQQQQQQ----------GMR
Svevo (Gli-γ3b)           QRPFIQPSLQQRLNPCKNILLQQCKPASLVSSLWSIIWPQSDCQVMQQQCCQELAQIPQQLQCAAIHSVVHSIIMQQQQQQQQQQQQ---------QGMH
AY338391_cv._TD92         QPSLIQQSLQQQLNPCKNFLLQQCKPVSLVSSLWSIILPPSDCQVMRQQCCQQLAQIPQQLQCAAIHSVVHSIIMQQEQQEQL-------------QGVQ
AY338394_cv._TD1A23       QPSLIQQSLQQQLNPCKNFLLQQCKPVSLVSSLWSIILPPSDCQVMRQQCCQQLAQIPQQLQCAAIHSVVHSIIMQQEQQEQL-------------QGVQ
AY338393_cv._TD1A23       QPSLIQQSLQQQLNPCKNFLLQQCKPVSLASSLWSIILPPSDCQVMRQQCCQQLAQIPQQLQCAAIHSVVHSIIMQQEQQEQL-------------QGVQ
FJ006578_cv._AS2255       QPSLIQQSLQQQLNPCKNFLLQQCKPVSLVSSLWSIILPPSDCQVMRQQCCQQLAQIPQQLQCAAIHSVMYSIIMQQEQQEQL-------------QGVQ
FJ006585_cv._AS2255       QPSLIQQSLQQQLNPCKNFLLQQCKPVSLVSSLWSIILPPSDCQVMRQQCCQQLAQIPQQLQCAAIHSVMHSIIMQQEQQEQL-------------QGVQ
FJ006588_cv._AS2255       QPSLIQQSLQQQLNPCKNFLLQQCKPVSLVSSLWSIILPPSDCQVVRQQCCQQLAQIPQQLQCAAIHSVMHSIIMQQEQQEQL-------------QGVQ
Svevo (Gli-γ5b)           QPSLIQQSLQQQLNPCKNFLLQQCKPVSLVSSLWSIILPPSDCQVMRQQCCQQLAQIPQQLQCAAIHSVVHSIIMQQEQQEQL-------------QGVQ
AY338392_cv._TD92         QQLLIQPYLQQQMNPCKNYLLQQCNPVSLVSSLVSMILPRSDCQVMRQQCCQQLAQIPQQLQCAAIHSVVHSIIMQQEQQEQL-------------QGVQ
Svevo (Gli-γ1a)           QQSLIQPYLQQQMNPCKNYLLQQCNPVSLVSSLVSMILPRSDCQVMRQQCCQQLAQIPQQLQCAAIHGVVHSIIMQQEQQQ---------------QGIQ
FJ006577_cv._AS2255       QQPAIQSFLQQQMNPCKNFLLQQCNHVSLVSSLVSIILPRSDCQVMQQQCCQQLAQIPQQLQCAAIHSVAHSIIMQQEQQQ----------------GVP
Svevo (Gli-γ1b)           QQPAIQSFLQQQMNPCKNFLLQQCNHVSLVSSLVSIILPRSDCQVMQQQCCQQLAQIPQQLQCAAIHSVAHSIIMQQEQQQ----------------GVP
X77963_cv._pTD9           QQPAIQSFLQQQMNPCKNFLLQQCNHVSLVSSLVSIILPRSDCQVMQQQCCQQLAQIPQQLQCAAIHSVAHSIIMQQEQQQ----------------GVP
X53412_cv._Langdon        QQPAIQSFLQQQMNPCKNFLLQQCNHVSLVSSLVSIILPRSDCQVMQQQCCQQLAQIPQQLQCAAIHSVAHSIIMQQEQQQ----------------GVP
Svevo (Gli-γ2b)           QQWMIQSFLQQQMNPCKNFLLQQCNPVSLVSSLVSIILPRSDCQLMQQQCCQQLAQIPQQLQCAAIHSVAHSIVMQQEQQR----------------GVQ
FJ006580_cv._AS2255       QQPAIQSFLQQQMNPCKNFLLQQCNHVSLVSSLVSIILPRSDCQVMQQQCCQQLAQIPRQLQCTAIHSVVHAIIMQQEQQ-----------------GIQ
Svevo (Gli-g2a*)          QQLLIQPYLQQQMNPCKNYLLQQCNPVSLVSSLVSMILPRSDCQVMQQQCCQQLAQIPRQLQCTAIHSVVHAIIMQQEQQ-----------------GIQ
FJ006586_cv._AS2255       QQPLIQPYLQQQMNPCKNYLLQQCNPVSLVSSLVSMILPRSDCQVMQQQCCQQLAQIPRQLQCAAIHSVVHSIIMQQEQQQ----------------GIQ
FJ006581_cv._AS2255       QQPLIQPYLQQQMNPCKNYLLQQCNPVSLVSSLVSMILPRSDCQVMQQQCCQQLAQIPRQLQCAAIHSVVHSIVMQQEQQQ----------------GIQ
FJ006582_cv._AS2255       QQPLIQPYLQQQMNPCKNYLLQQCNPVSLVSSLASMILPRSDCQVMQQQCCQQLAQIPRQLQCAAIHSVVHSIVMQQEQQQ----------------GIQ
FJ006584_cv._AS2255       QQPLIQPYLQQQMNPCKNYLLQQCNPVSLVSSLVSMILPRSDCQVMQQQCCQQLAQIPRQLQCAAIHSVVHSIVMQQEQQQ----------------GIQ
Svevo (Gli-γ3a)           QQPLIQPYLQQQMNPCKNYLLQQCNPVSLVSSLVSMILPRSDCQVMQQQCCQQLAQIPRQLQCAAIHSVVHSIVMQQEQQQ----------------GIQ
FJ006583_cv._AS2255       QQPLIQPYLQQQMNPCKNYLLQQCNPVSLVSSLVSMILPRSDCQVMQQQCCQQLAQIPRQLQCAAIHSVVHSIIMQQEQQQ----------------GIQ
FJ006587_cv._AS2255       QQPLIQPYLQQQMNPCKNYLLQQCNPVSLVSSLVSMILPRSDCQVMQQQCCQQLAQIPRQLQCAAIHSVVHSIIMQQEQQQ----------------GIQ
FJ006579_cv._AS2255       QQPLIQPYLQQQMNPCKNYLLQQCNPVSLVSSLVSMILPRSDCQVMQQQCCQQLAQIPRQLQCAAIHSVVHSIIMQQEQQQ----------------GIQ
Svevo (Gli-γ4a)           QQPLIQPYLQQQMNPCKNYLLQQCNPVSLVSSLVSMILPRSDCQVMQQQCCQQLAQIPRQLQCAAIHSVVHSIIMQQEQQQ----------------GIQ
```

```
                                 V
                                  *       *
                          301                                                                     373
AJ389699_cv._Astodur      ILLPLYQQQQVGQGTLVQGQGIIQPQQPAQLEAIRSLVLQTLPTMCNVYVPPECSIIKAPL------------
AJ389700_cv._Duilio       ILLPLYQQQQVGQGTLVQGQGIIQPQQPAQLEAIRSLVLQTLPTMCNVYVPPECSIIKAPL------------
AJ389701_cv._DT433        -------------------------------------------------------------------------
Svevo (Gli-γ4b*)          ILLPLYQQQQVGQGTLVQGQGIIQPQQPAQLEAIRSLVLQTLPTMCNVYVPPECSIIKAPFASIVTGIGGQ--
AJ389703_cv._Rugby        ILL----------------------------------------------------------------------
AJ389704_cv._Wascana      ILLPLYQQQQVGQGTLVQGQGIIQPQHPAQLEAIRSLVLQTLPTMCNVYVPPECSIIKAPL------------
Svevo (Gli-γ3b)           ILLTLSQQQQLGQGTLVQGQGIIQPQQLAQLEAIRSLVLQTLPTMCNVYVPPECSIIRAPFASIVAGIGGQ--
AY338391_cv._TD92         ILVPLSQQQQVGQGILVQGQGIIQPQQPAQLEVIRSLVLQTLPTMCNVYVPPYCSTIRAPFASIVAGIGGQYR
AY338394_cv._TD1A23       ILVPLSQQQQVGQGILVQGQGIIQPQQPAQLEVIRSLVLQTLPTMCNVYVPPYCSTIRAPFASIVAGIGGQYR
AY338393_cv._TD1A23       ILVPLSQQQQVGQGILVQGQGIIQPQQPAQLEVIRSLVLQTLPTMCNVYVPPYCSTIRAPFASIVAGIGGQYR
FJ006578_cv._AS2255       ILVPLSQQQQVDQGILVQGQGIIQPQQPAQLEVIRSLVLQTLPTMCNVYVPPYCSTIRAPFASIVASIGGQ--
FJ006585_cv._AS2255       ILVPLSQQQQVDQGILVQGQGIIQPQQPAQLEVIRSLVLQTLPTMCNVYVPPYCSTIRAPFASIVASIGGQ--
FJ006588_cv._AS2255       ILVPLSQQQQVDQGILVQGQGIIQPQQPAQLEVIRSLVLQTLPTMCNVYVPPYCSTIRAPFASIVASIGGQ--
Svevo (Gli-γ5b)           ILVPLSQQQQVGQGILVQGQGIIQPQQPAQLEVIRSLVLQTLPTMCNVYVPPYCSTIRAPFASIVASIGGQ--
AY338392_cv._TD92         ILVPLSQQQQVGQGILVQGQGIIQPQQPAQLEVIRSLVLQTLPTMCNVYVPPYCSTIRAPFASIVAGIGGQYR
Svevo (Gli-γ1a)           IMRPLFQ--------LIQGQGIIQPQQPAQLEVIRSLVLGTLPTMCNVFVPPECSTTKAPFASIVADIGGQ--
FJ006577_cv._AS2255       ILRPLFQ--------LAQGLGIIQPQQPAQLEGIRSLVLKTLPTMCNVYVPPDCSTINVPYANIDAGIGGQ--
Svevo (Gli-γ1b)           ILRPLFQ--------LAQGLGIIQPQQPAQLEGIRSLVLKTLPTMCNVYVPPDCSTINVPYANIDAGIGGQ--
X77963_cv._pTD9           ILRPLFQ--------LAQGLGIIQPQQPAQLEGIRSLVLKTLPTMCNVYVPPDCSTINVPYANIDAGIGGQ--
X53412_cv._Langdon        ILRPLFQ--------LAQGLGIIQPQQPAQLEGIRSLVLKTLPTMCNVY------------------------
Svevo (Gli-γ2b)           ILRPLFQ--------LAQGLGIIQPQQPAQLEGIRSLVLKTLPTMCNVYVPPDCSTINVPYASIDAVIGGQ--
FJ006580_cv._AS2255       ILRPLFQ--------LVQGQGIIQPQQPAQYEVIRSLVLRTLPNMCNVYVRPDCSTINAPFASIVAGIGGQ--
Svevo (Gli-γ2a*)          ILRPLFQ--------LVQGQGIIQPQQPAQYEVIRSLVLRTLPNMCNVYVRPDCSTINAPFASIVAGIGGQ--
FJ006586_cv._AS2255       ILRPLFQ--------LVQGQGIIQPQQPAQYEVIRSLVLRTLPNMCNVYVRPDCSTINAPFASIVAGISGQ--
FJ006581_cv._AS2255       ILRPLFQ--------LIQGQGIIQPQQPAQYEVIRSLVLRTLPNMCNVYVRPDCSTINAPFASIVAGISGQ--
FJ006582_cv._AS2255       ILRPLFQ--------LIQGQGTIQPQQPAQYEVIRSLVLRTLPNMCNVYVRPDCSTINAPFASIVAGISGQ--
FJ006584_cv._AS2255       ILRPLFQ--------LIQGQGIIQPQQPAQYEVIRSLVLRTLPNMCNVYVRPDCSTINAPFASIVAGISGQ--
Svevo (Gli-γ3a)           ILRPLFQ--------LIQGQGIIQPQQPAQYEVIRSLVLRTLPNMCNVYVRPDCSTINAPFASIVAGISGQ--
FJ006583_cv._AS2255       ILRPLFQ--------LVQGQGIIQPQQPAQYEVIRSLVLRTLPNMCNVYVRPDCSTINAPFASIVAGISGQ--
FJ006587_cv._AS2255       ILRPLFQ--------LVQGQGIIQPQQPAQYEVIRSLVLRTLPNMCNVYVRPDCSTINAPFASIVAGISGQ--
FJ006579_cv._AS2255       ILRPLFQ--------LVQGQGIIQPQQPAQYEVIRSLVLRTLPNMCNVYVRPDCSTINAPFASIVAGISGQ--
Svevo (Gli-γ4a)           ILRPLFQ--------LVQGQGIIQPQQPAQYEVIRSLVLRTLPNMCNVYVRPDCSTINAPFASIVAGISGQ--
```
