# Supplementary material for: Genome-Wide Identification, Characterization and Expression Pattern Analysis of the γ-Gliadin Gene Family in the Durum Wheat (Triticum durum Desf.) Cultivar Svevo
Source: Genes (Basel). 2021 Oct 29;12(11):1743. doi: 10.3390/genes12111743 (PMC8621147; doi:10.3390/genes12111743)
Supplement: Supplementary file 1 [file genes-12-01743-s001.zip › Supplemetary materials/Table S1.pdf]

Table S1. Primer pairs and optimized conditions used in the qRT-PCR, and size of the amplified products.

Gene	Primer orientation	Primer sequence (5'-3')	Product size (bp)	Ta	Tm	E	R^2
Gli-γ1a	Fw	ATCATGCGGCCACTTTTC	139	60	83.2	2.10	0.997
	Rev	AGCACTCAGGTGGAACAAATACA					
Gli-γ3a+Gli-γ4a	Fw	CAACTAGCACAGATTCCTCGC	145	62	85.0	2.00	0.996
	Rev	GTTGGATGATGCCCTGACCT					
Gli-γ1b	Fw	CGAGCAACCACAACGAACTA	79	60	79.8	2.01	0.974
	Rev	TTCGGGTTGGGGAAATGT					
Gli-γ2b	Fw	GTCGACCCTAGCAGCCG	79	60	83.2	1.93	0.991
	Rev	GTGGTTGCTGGGAGAATGGT					
Gli-γ3b	Fw	CTCCAGCAACAACGACCA	195	60	84.0	2.01	0.998
	Rev	CTGGAGCTGCTGAGGAATCT					
Gli-γ5b	Fw	GTGTCATCCCTCTGGTCAATC	249	62	85.3	1.89	0.997
	Rev	AGGTTGGATGATGCCTTGA					
CDC	Fw	CAGCTGCTGACTGAGATGGA	227	60	80.5	2.03	0.995
	Rev	ATGTCTGGCCTGTTGGTAGC					
RLI	Fw	TTGAGCAACTCATGGACCAG	242	60	77.8	2.19	0.996
	Rev	GCTTTCCAAGGCACAAACAT					
ADP-RF	Fw	TCTCATGGTTGGTCTCGATG	276	60	82.8	2.07	0.987
	Rev	GGATGGTGGTGACGATCTCT					
