# Supplementary material for: Genome-Wide Identification, Characterization and Expression Pattern Analysis of the γ-Gliadin Gene Family in the Durum Wheat (Triticum durum Desf.) Cultivar Svevo
Source: Genes (Basel). 2021 Oct 29;12(11):1743. doi: 10.3390/genes12111743 (PMC8621147; doi:10.3390/genes12111743)
Supplement: Supplementary file 1 [file genes-12-01743-s001.zip › Supplemetary materials/Table S2.pdf]

Table S2. Manual annotation of the regions from the homeologous A and B genome of the durum wheat cv. Svevo harboring the γ-gliadin genes. The ancestral genes are highlighted in orange.

A genome			B genome			Rice
**Annotation**	**Start**	**End**	**Annotation**	**Start**	**End**	
**E3 ubiquitin-protein ligase**	**4110013**	**4115442**	**E3 ubiquitin-protein ligase**	**4058971**	**4066512**	**LOC_Os05g01230**
**Mei2-like protein**	**4118433**	**4125560**	**Mei2-like protein**	**4069522**	**4076464**	**LOC_Os05g01240**
**Charged multivesicular body protein**	**4323044**	**4325693**	**Charged multivesicular body protein**	**4189942**	**4192587**	**LOC_Os05g01250**
Putative LRR receptor-like serine/threonine-protein kinase	4327378	4330809	Putative LRR receptor-like serine/threonine-protein kinase	4193737	4197062	
F-box/LRR-repeat protein 14-like	4333931	4335661	B3 domain-containing protein family	4198192	4198479	
Putative LRR receptor-like serine/threonine-protein kinase	4374088	4375420	F-box/LRR-repeat protein	4215102	4218006	
Putative LRR receptor-like serine/threonine-protein kinase	4379443	4381974	Wall-associated receptor kinase	4234355	4252395	
Protein SUPPRESSOR OF GENE SILENCING 3	4392564	4396390	Putative LRR receptor-like protein kinase family protein	4262215	4264249	
Uncharacterized protein	4435606	4436067	Putative LRR receptor-like protein kinase family protein	4264292	4265965	
Putative LRR receptor-like serine/threonine-protein kinase	4441777	4444301	Putative LRR receptor-like protein kinase family protein	4274743	4278215	
Putative LRR receptor-like serine/threonine-protein kinase	4477765	4479939	Putative LRR receptor-like protein kinase family protein	4299647	4301050	
Ankyrin-1 like	4494815	4498583				
F-box/LRR-repeat protein 14-like	4508576	4510033				
Putative LRR receptor-like serine/threonine-protein kinase	4526143	4529573				
Putative LRR receptor-like serine/threonine-protein kinase	4538905	4541830				
Carboxyl-terminal peptidase, putative (DUF239)	4545278	4548303				
Putative LRR receptor-like serine/threonine-protein kinase	4572368	4575403				

**A genome**			**B genome**			**Rice**
Putative LRR receptor-like serine/threonine-protein kinase	4593717	4597257				
Putative LRR receptor-like serine/threonine-protein kinase	4604918	4608389				
δ-gliadin (*Gli-δ1a**)	4613285	4614202	γ-gliadin (*Gli-γ1b*)	4313476	4314384	
δ-gliadin (*Gli-δ2a**)	4631881	4632772	δ-gliadin (*Gli-δ1b**)	4341737	4342690	
γ-gliadin (*Gli-γ1a*)	4687840	4688874	γ-gliadin (*Gli-γ2b*)	4355195	4356088	
γ-gliadin (*Gli-γ2a**)	4744130	4744969	γ-gliadin (*Gli-γ3b*)	4481997	4482992	
γ-gliadin (*Gli-γ3a*)	4871918	4872775	γ-gliadin (*Gli-γ4b**)	4514026	4515024	
γ-gliadin (*Gli-γ4a*)	4879635	4880492	γ-gliadin (*Gli-γ5b*)	4518944	4519819	
**Cyclophilin-like protein**	**4883974**	**4886532**	**Cyclophilin-like protein**	**4524929**	**4527581**	**LOC_Os05g01270**
Avenin-like (*Av-1a**)	4887801	4888553	Avenin-like (*Av-1b**)	4528580	4529044	
Avenin-like (*Av-2a*)	4891405	4892007	Avenin-like (*Av-2b*)	4543310	4543921	
**Protein of unknown function (DUF3339)**	**4892935**	**4893153**	**Protein of unknown function (DUF3339)**	**4545088**	**4545306**	**LOC_Os05g01280**
