# Supplementary material for: Genome-Wide Identification, Characterization and Expression Pattern Analysis of the γ-Gliadin Gene Family in the Durum Wheat (Triticum durum Desf.) Cultivar Svevo
Source: Genes (Basel). 2021 Oct 29;12(11):1743. doi: 10.3390/genes12111743 (PMC8621147; doi:10.3390/genes12111743)
Supplement: Supplementary file 1 [file genes-12-01743-s001.zip › Supplemetary materials/Table S3.pdf]

Table S3. Percentage of glutamine, proline and essential amino acids in the γ-gliadin proteins deduced from the seven full-length genes identified in the durum wheat cv. Svevo. Percentages were calculated using Expasy ProtParam tool [21].

	Gln	Pro	His	Ile	Leu	Lys	Met	Thr	Phe	Trp	Val	Total essential amino acids
**Gli-γ1a**	32.7	17.1	1.2	5.9	6.9	0.9	2.2	3.7	5.6	0.3	4.4	31.1
**Gli-γ3a**	30.5	16.1	1.1	6.0	6.7	0.7	2.8	3.2	5.3	0.4	4.9	31.1
**Gli-γ4a**	30.5	16.1	1.1	6.3	6.7	0.7	2.8	2.8	5.3	0.4	4.9	31.0
**Gli-γ1b**	29.1	16.6	2.3	6.0	7.0	1.0	2.3	4.6	5.3	0.3	4.0	32.8
**Gli-γ2b**	29.4	16.2	1.4	6.1	7.8	1.0	2.4	3.4	5.1	0.7	4.4	32.3
**Gli-γ3b**	34.1	15.9	1.5	6.1	7.9	0.9	2.1	3.7	4.9	0.9	3.7	31.7
**Gli-γ5b**	30.2	15.1	1.4	6.9	8.2	1.4	2.1	3.1	4.5	0.7	5.5	33.8
