# Supplementary material for: Genome-Wide Identification, Characterization and Expression Pattern Analysis of the γ-Gliadin Gene Family in the Durum Wheat (Triticum durum Desf.) Cultivar Svevo
Source: Genes (Basel). 2021 Oct 29;12(11):1743. doi: 10.3390/genes12111743 (PMC8621147; doi:10.3390/genes12111743)
Supplement: Supplementary file 1 [file genes-12-01743-s001.zip › Supplemetary materials/Table S4.pdf]

Table S4. Classification of δ-gliadin genes, identified in different durum and bread wheat cvs., orthoulogous to the Svevo *Gli-δ1a**, *Gli-δ2a** and *Gli-δ1b** genes. The gene sequences of bread wheat cvs. were retrieved from the Wheat URGI database (https://wheat-urgi.versailles.inra.fr/), whereas those of durum wheat cvs. other than Svevo were retrieved from the Ensembl Plants database (https://plants.ensembl.org/index.html).

**Cultivar**	**δ-gliadin genes**					
	Gli-δ1a*		***Gli-δ2a****		***Gli-δ1b****	
	**Gene**	**Pseudogene**	**Gene**	**Pseudogene**	**Gene**	**Pseudogene**
Durum wheat cvs.						
Svevo		✓		✓		✓
Cappelli		✓		✓		✓
Strongfield		✓		✓		✓
Bread wheat cvs.						
Chinese Spring		✓		✓	✓	
Cadenza		✓		✓		✓
Claire		✓		✓		✓
Jagger		✓		✓	✓	
Julius		✓		✓		✓
Lancer		✓		✓		✓
Landmark	✓			✓		✓
Mace	✓			✓	✓	
Paragon		✓		✓		✓
Robigus	✓			✓		✓
Stanley		✓		✓		✓
Weebill		✓		✓	✓	
